# Diketopiperazine-Based, Flexible Tadalafil Analogues: Synthesis, Crystal Structures and Biological Activity Profile

**DOI:** 10.3390/molecules26040794

**Published:** 2021-02-03

**Authors:** Adam Mieczkowski, Elżbieta Speina, Damian Trzybiński, Maria Winiewska-Szajewska, Patrycja Wińska, Ewelina M. Borsuk, Małgorzata Podsiadła-Białoskórska, Tomasz Przygodzki, Krzysztof Drabikowski, Lidia Stanczyk, Igor Zhukov, Cezary Watala, Krzysztof Woźniak

**Affiliations:** 1 Institute of Biochemistry and Biophysics, Polish Academy of Sciences, Pawinskiego 5a, 02-106 Warsaw, Poland; elasp@ibb.waw.pl (E.S.); mwin@ibb.waw.pl (M.W.-S.); e.szmajda@ibb.waw.pl (E.M.B.); weronika@ibb.waw.pl (M.P.-B.); drabikowski@ibb.waw.pl (K.D.); igor@ibb.waw.pl (I.Z.); 2 Biological and Chemical Research Centre, University of Warsaw, Żwirki i Wigury 101, 02-089 Warsaw, Poland; dtrzybinski@cnbc.uw.edu.pl (D.T.); kwozniak@chem.uw.edu.pl (K.W.); 3 Faculty of Chemistry, Warsaw University of Technology, Noakowskiego 3, 00-664 Warsaw, Poland; pwinska@ch.pw.edu.pl; 4 Department of Haemostatic Disorders, Chair of Biomedical Sciences, Faculty of Health Sciences, Medical University of Lodz, 6/8 Mazowiecka Street, 92-235 Lodz, Poland; tomasz.przygodzki@umed.lodz.pl (T.P.); lidiastanczyk57@gmail.com (L.S.); cezary.watala@umed.lodz.pl (C.W.)

**Keywords:** short peptide, diketopiperazine, tadalafil, PDE5 inhibitor, cytotoxicity

## Abstract

Phosphodiesterase 5 (PDE5) is one of the most extensively studied phosphodiesterases that is highly specific for cyclic-GMP hydrolysis. PDE5 became a target for drug development based on its efficacy for treatment of erectile dysfunction. In the present study, we synthesized four novel analogues of the phosphodiesterase type 5 (PDE5) inhibitor—tadalafil, which differs in (i) ligand flexibility (rigid structure of tadalafil vs. conformational flexibility of newly synthesized compounds), (ii) stereochemistry associated with applied amino acid building blocks, and (iii) substitution with bromine atom in the piperonyl moiety. For both the intermediate and final compounds as well as for the parent molecule, we have established the crystal structures and performed a detailed analysis of their structural features. The initial screening of the cytotoxic effect on 16 different human cancer and non-cancer derived cell lines revealed that in most cases, the parent compound exhibited a stronger cytotoxic effect than new derivatives, except for two cell lines: HEK 293T (derived from a normal embryonic kidney, that expresses a mutant version of SV40 large T antigen) and MCF7 (breast adenocarcinoma). Two independent studies on the inhibition of PDE5 activity, based on both pure enzyme assay and modulation of the release of nitric oxide from platelets under the influence of tadalafil and its analogues revealed that, unlike a reference compound that showed strong PDE5 inhibitory activity, the newly obtained compounds did not have a noticeable effect on PDE5 activity in the range of concentrations tested. Finally, we performed an investigation of the toxicological effect of synthesized compounds on *Caenorhabditis elegans* in the highest applied concentration of **6a,b** and **7a,b** (160 μM) and did not find any effect that would suggest disturbance to the life cycle of *Caenorhabditis elegans*. The lack of toxicity observed in *Caenorhabditis elegans* and enhanced, strengthened selectivity and activity toward the MCF7 cell line made **7a,b** good leading structures for further structure activity optimization and makes **7a,b** a reasonable starting point for the search of new, selective cytotoxic agents.

## 1. Introduction

**Figure 1:**
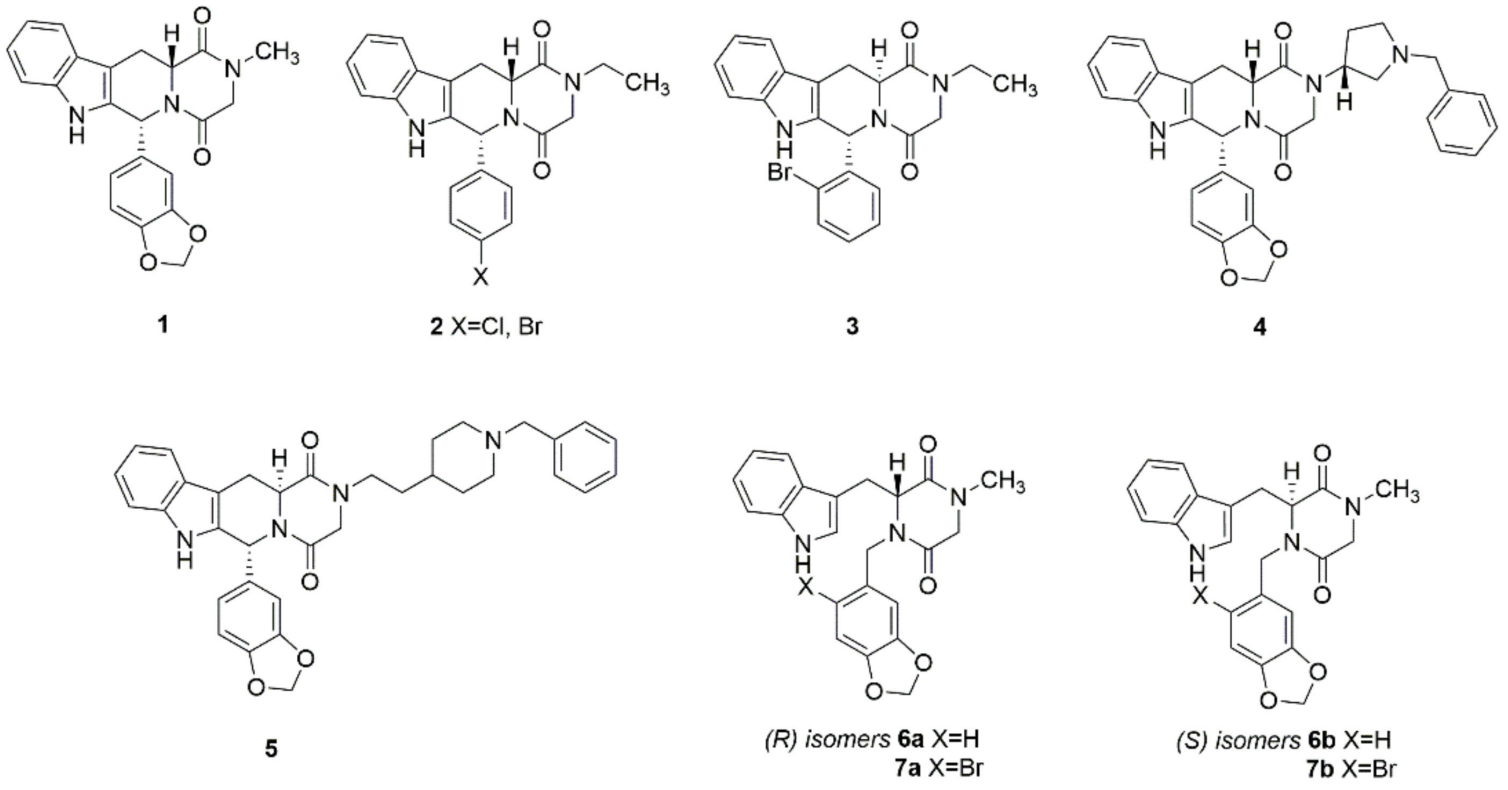
Tadalafil (**1**), its equipotent phosphodiesterase 5 (PDE5) inhibition analogues **2**, analogues with cytotoxic **3**, antiplasmodial **4**, procognitive **5** activities and newly synthesized compounds **6a,b** and **7a,b**.

Phosphodiesterase type 5 (PDE5) also called the cGMP-specific, cGMP-binding PDE, is the major cGMP-hydrolyzing PDE in platelets, lung, and the vascular smooth muscle of the penile corpus cavernosum. This ubiquitous enzyme decreases 3′,5′-cyclic guanosine monophosphate (cGMP) or 3′,5′-cyclic adenosine monophosphate (cAMP) in target cells by catalyzing the hydrolysis of these second messengers. PDE 5 inhibitors stop the enzyme in blood vessel walls from working properly and cause blood vessels muscle to relax. Tadalafil (**1**) (Figure 1) is a highly potent and highly selective phosphodiesterase type 5 (PDE5) inhibitor [1,2] which has gained wide recognition as an efficient agent for treatment of erectile dysfunction [3,4,5] with limited adverse effects [6] and a prolonged mode of action [7,8]. As a long-lasting PDE5 inhibitor, it was also approved in 2009 by the US Food and Drug Administration (FDA) for the treatment of pulmonary arterial hypertension (PAH) [9,10,11,12] and for the treatment of lower urinary tracts symptoms (LUTS) associated with benign prostatic hyperplasia (BPH) [13,14]. Koka et al. reported that tadalafil could be used as a protective agent in cancer treatment and could attenuate doxorubicin-induced cardiomyopathy without interfering with chemotherapeutic effect [15]. Tadalafil, exhibiting a weak cytotoxic effect, significantly enhanced the colchicine and paclitaxel sensitivity in MDR ABCB1-overexpressing KB-C2 cells and the vincristine and paclitaxel in HEK/ABCB1 cells [16]. Booth reported that in multiple tumor cell types, including anoikis resistant and stem cell-like cells, sildenafil (and tadalafil) interacted with celecoxib to promote killing in short-term assays and long-term colony formation assays [17]. Initially, it was assumed that tadalafil (and other PDE 5 inhibitors) could influence antiproliferation and proapoptotic mechanism in various carcinomas with special importance particularly in carcinoma cells with enhanced PDE5 expression [18]. A recent review [19] indicated the importance and the potential of tadalafil as an anticancer agent, which exhibited cytotoxic effects and the reduction of tumor growth in collateral [20,21], HNSCC [22] and thyroid [23] cancers, as well as improved survival in a murine model of brain lymphoma with rituximab treatment [24]. Finally, Kassel reported that tadalafil can improve clinical outcome of advanced melanoma patients by enhancing anti-tumor immunity and suggested its potential application in combined melanoma immunotherapy [25].

Consequently, in recent years many different analogues of tadalafil were synthesized [26,27] (Figure 1). Using computer-aided molecular modeling, **2** analogues were designed and synthesized, which exhibited equipotent PDE inhibition activity [28,29,30]. Abadi at al. reported [31] that bromine-modified series of tadalafil, such as compound **3**, demonstrate both strong PDE inhibition activity (IC_50_ = 0.06 μM for **3**) and a moderate cytotoxic effect against the colon cancer cell line HT29 (IC_50_ = 25.5 μM for **3**). The stereochemical effect on cytotoxic behavior was also determined, and surprisingly the presence of a chiral center derived from amino acid with the *S* configuration (l-tryptophan) resulted in more cytotoxic isomers than those derived from amino acid with the *R* configuration (d-tryptophan), even if such derivatives exhibited lower PDE inhibition activity. The structure of tadalafil was a starting point for the development of antiplasmodial agents, utilizing the structural differences between human and plasmodial PDE5 orthologues [32]. It was revealed that compound **4** kills the parasite via the inhibition of plasmodial PDE activity (pIC_50_ = 6.3 μM). These results prove that the cAMP/cGMP pathways could be desirable therapeutic targets against *Plasmodium falciparum* and antiplasmodial derivatives could act as inhibitors of the hydrolysis of cyclic nucleotides of the parasite. In 2018, Mao et al. and Ni et al. reported [33,34] the first example of dual-target inhibitors for AChE and PDE5 as potentially novel therapeutic agents for the treatment of Alzheimer’s disease (AD). The lead compound **5** has been confirmed to demonstrate memory-enhancing effects in AD mouse models, exhibiting AChE and PDE5A1 inhibition at IC_50_ = 0.032 μM and IC_50_ = 1.53 μM, respectively.

As the diketopiperazine framework is a well-recognized privileged structure, and a diketopiperazine ring is present in many bioactive compounds and drugs [35,36,37], we decided to design, synthesize and investigate the biological activity of some flexible tadalafil analogues based on the diketopiperazine scaffold on different biological models and compare their physicochemical and biological properties and features with the parent compound. In our study, we decided to investigate the influence and importance of inhibitor flexibility, stereochemical effect and substitution with bromine atom on biological activity and binding to PDE5. These studies are a continuation of our previous research on the development of new synthetic routes, and the search for biological activity of dilactam derivatives containing six-membered dilactam rings in their structure (hexahydro-2*H*-pyrazino[1,2-*a*]pyrazine-6,9-dione derivatives) [38], seven-membered dilactam rings (1,2,3,4,12,12a-hexahydrobenzo[e]pyrazino[1,2-*a*][1,3]diazepine-6,11-dione derivatives) [39,40,41,42] and eight-membered dilactam rings (dibenzo[*b*,*f*][1,5]diazocine-6,12(5*H*,11*H*)-dione and 5,12-dihydrodibenzo[*b*,*f*][1,4]diazocine-6,11-dione derivatives) [43,44,45,46].

## 2. Results

### 2.1. Chemistry

**Scheme 1:**
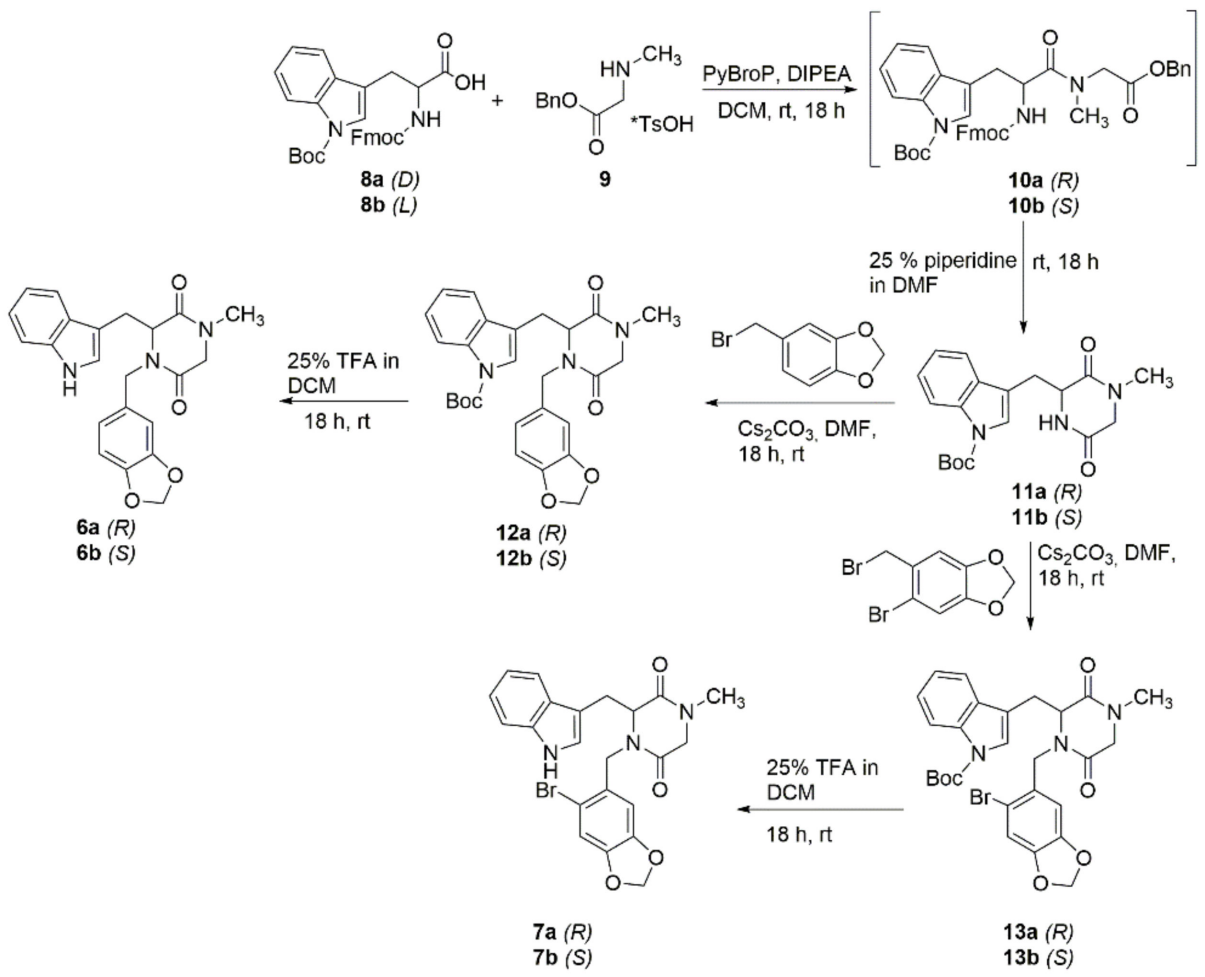
The synthesis of compounds **6a,b** and **7a,b**.

The designed synthetic path begins from a dipeptide formation reaction, where protected d-**8a** or l-tryptophane **8b** reacts with sarcosine benzyl ester tolenesulfonate (**9**) in the presence of PyBroP and DIPEA (Scheme 1). The reaction, monitored by TLC and LRMS(ESI) spectra, was completed after 18 h. Purification of intermediates **10a,b** produced unsatisfactory results, thus the obtained dipeptides **10a,b** were directly treated with 25% piperidine in dichloromethane, which resulted in the removal of the Fmoc group. The concomitant cyclisation to the diketopiperazine led to the products **11a,b** easily isolated and purified by column chromatography. Obtained diketopiperazine intermediates **11a,b** were then treated with 5-(bromomethyl)benzo[*d*][1,3]dioxole or 5-bromo-6-(bromomethyl)benzo[*d*][1,3]dioxole, in the presence of cesium carbonate, which led to the formation of the appropriate *N*-benzylated derivatives **12a,b** and **13a,b**. An initial attempt to use potassium carbonate instead of cesium carbonate did not yield the requested products. The final step, the removal of the Boc group, was performed by treating Boc derivatives **12a,b** and **13a,b** with 25% TFA in DCM. The obtained products **6a,b** and **7a,b** were isolated and purified by column chromatography and further used in biophysical and biochemical studies.

### 2.2. Crystallographic Studies

Attempts to obtain single-crystals suitable for diffractometric measurements of all synthesized compounds were undertaken, however, the goal was achieved only in four cases. Compounds **11a**, **12a** and **1** were obtained by slow evaporation from butanol-2 solution, while **6a** was obtained from slow evaporation from methanol.

**Figure 2:**
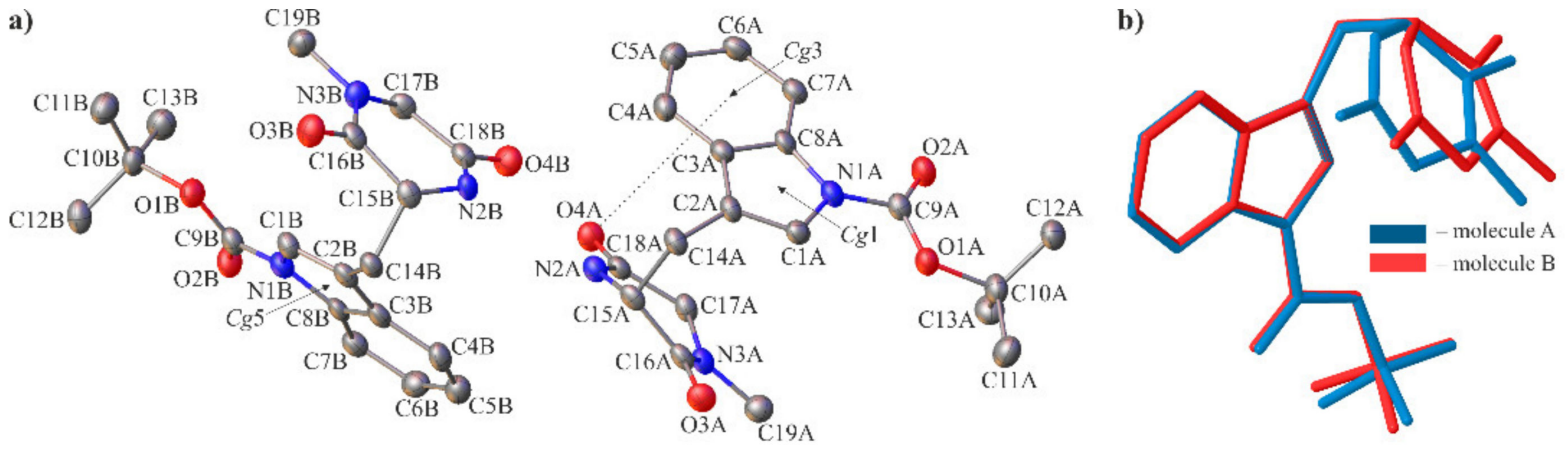
Asymmetric unit of the crystal lattice with crystallographic numbering (**a**) and superimposed molecules of **11a** (**b**). Displacement ellipsoids are drawn at the 50% probability level and the H-atoms were omitted for clarity. The intramolecular C=O⋯π contact is represented by the dotted line. *Cg*1, *Cg*3 and *Cg*5 denote the geometric centres of gravity of the aromatic rings delineated by the N1A/C1A–C3A/C8A, C3A–C8A and N1B/C1B–C3B/C8B atoms, respectively.

**Figure 3:**
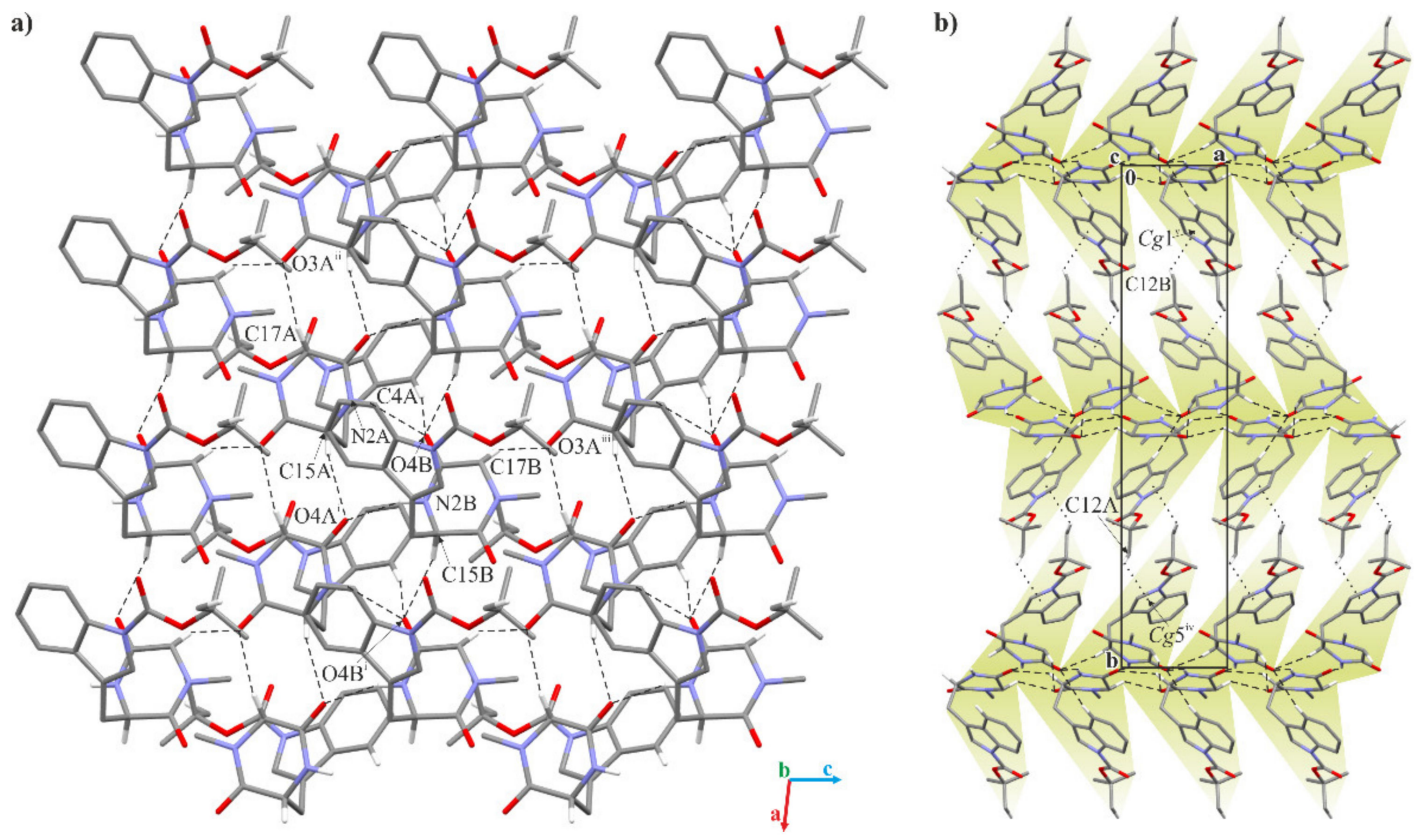
Arrangement of molecules of **11a** in the crystal, where: (**a**) layer of molecules spreading along (001) plane; (**b**) general view on layered supramolecular architecture of compound viewed along *c*-direction (single layers are highlighted in light-green). The H-atoms not involved in intermolecular interactions have been omitted for clarity. The N–H⋯O and C–H⋯O hydrogen bonds are represented by dashed lines, while the C–H⋯π contacts by dotted lines. Symmetry codes: (i) *x* + 1, *y*, *z*; (ii) *x* − 1, *y*, *z*; (iii) *x*, *y*, *z* + 1; (iv) −*x* + 1, *y* + 1/2, −*z*; (v) −*x* + 1, *y* − 1/2, −*z* + 1.

**Figure 4:**
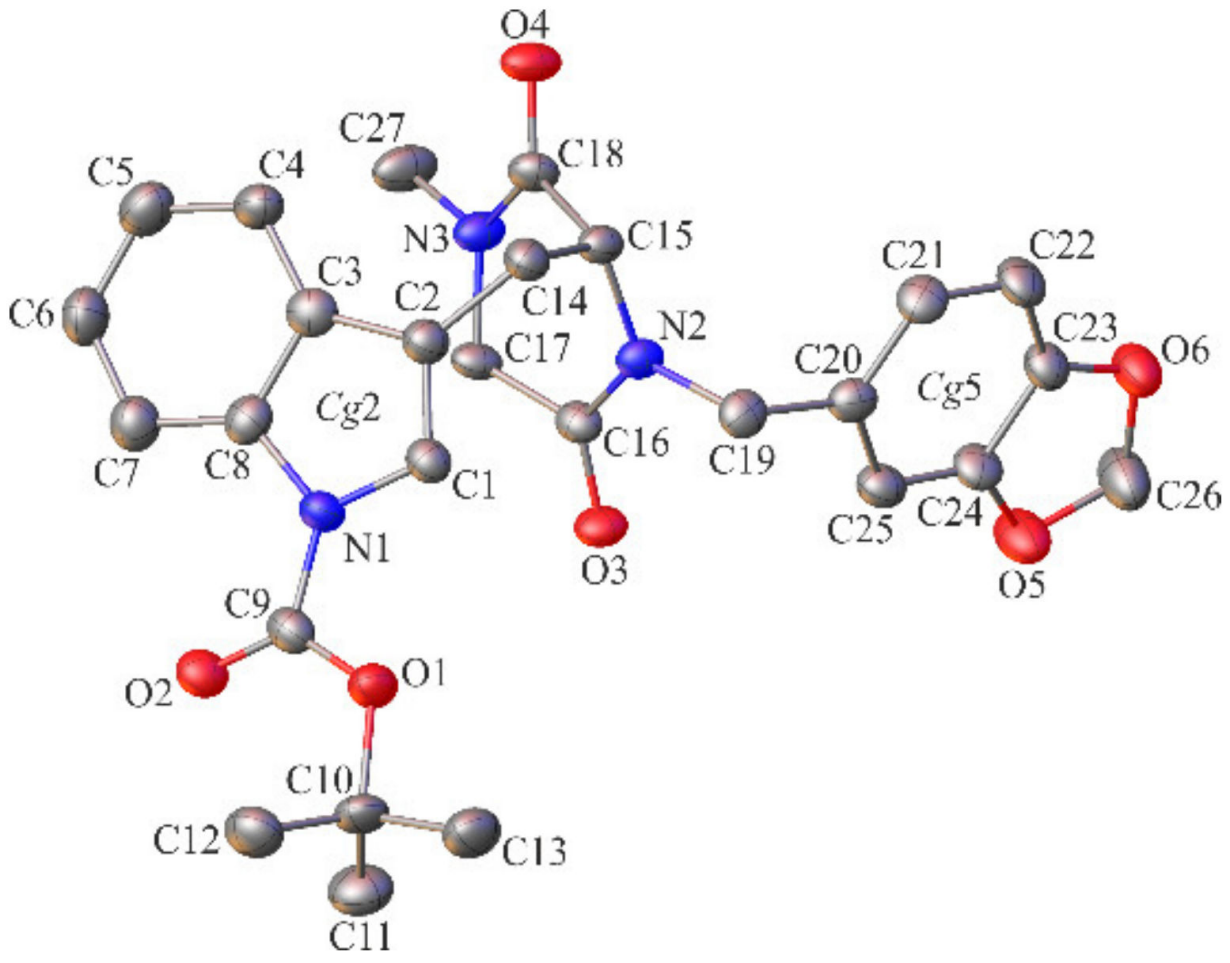
Asymmetric unit of the crystal lattice of **12a** with crystallographic numbering. Displacement ellipsoids are drawn at the 50% probability level and the H-atoms were omitted for clarity. *Cg*2 and *Cg*5 denote the geometric centres of gravity of the aromatic rings delineated by the N1/C1–C3/C8 and C20–C25 atoms, respectively.

**Figure 5:**
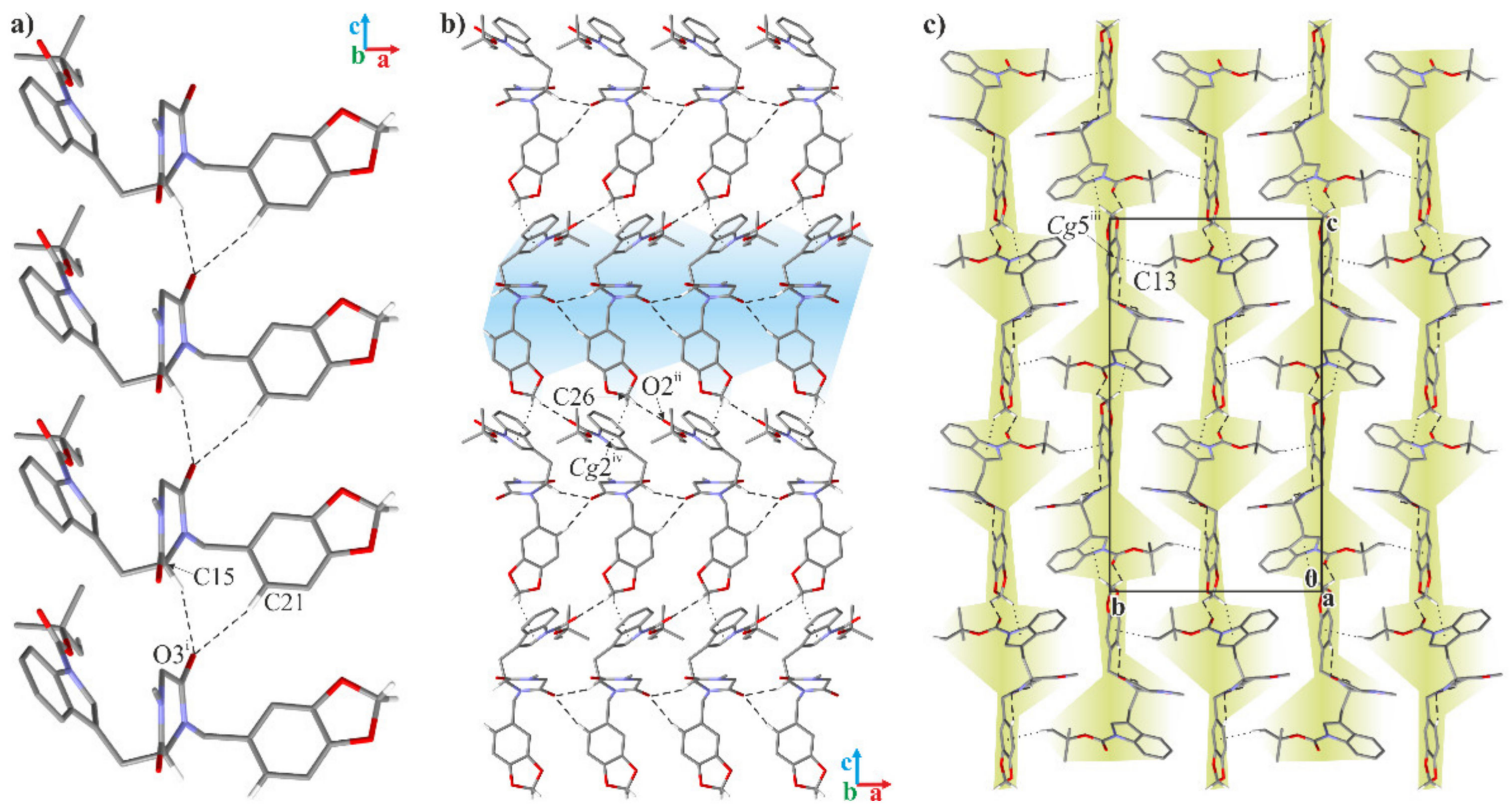
Arrangement of molecules of **12a** in the crystal, where: (**a**) chain of molecules running along [001] direction; (**b**) single layer of molecules spreading along (100) plane (single chain of molecules is highlighted in blue); (**c**) general view on layered supramolecular architecture of compound viewed along a-direction (single-layers are highlighted in light-green). The H-atoms not involved in intermolecular interactions have been omitted for clarity. The C–H⋯O hydrogen bonds are represented by dashed lines, while the C–H⋯π contacts by dotted lines. Symmetry codes: (i) x − 1, y, z; (ii) −x + 3/2, −y + 1, z − 1/2; (iii) −x + 1, y + 1/2, −z + 3/2; (iv) −x + 1/2, −y + 1, −z + 3/2.

**Figure 6:**
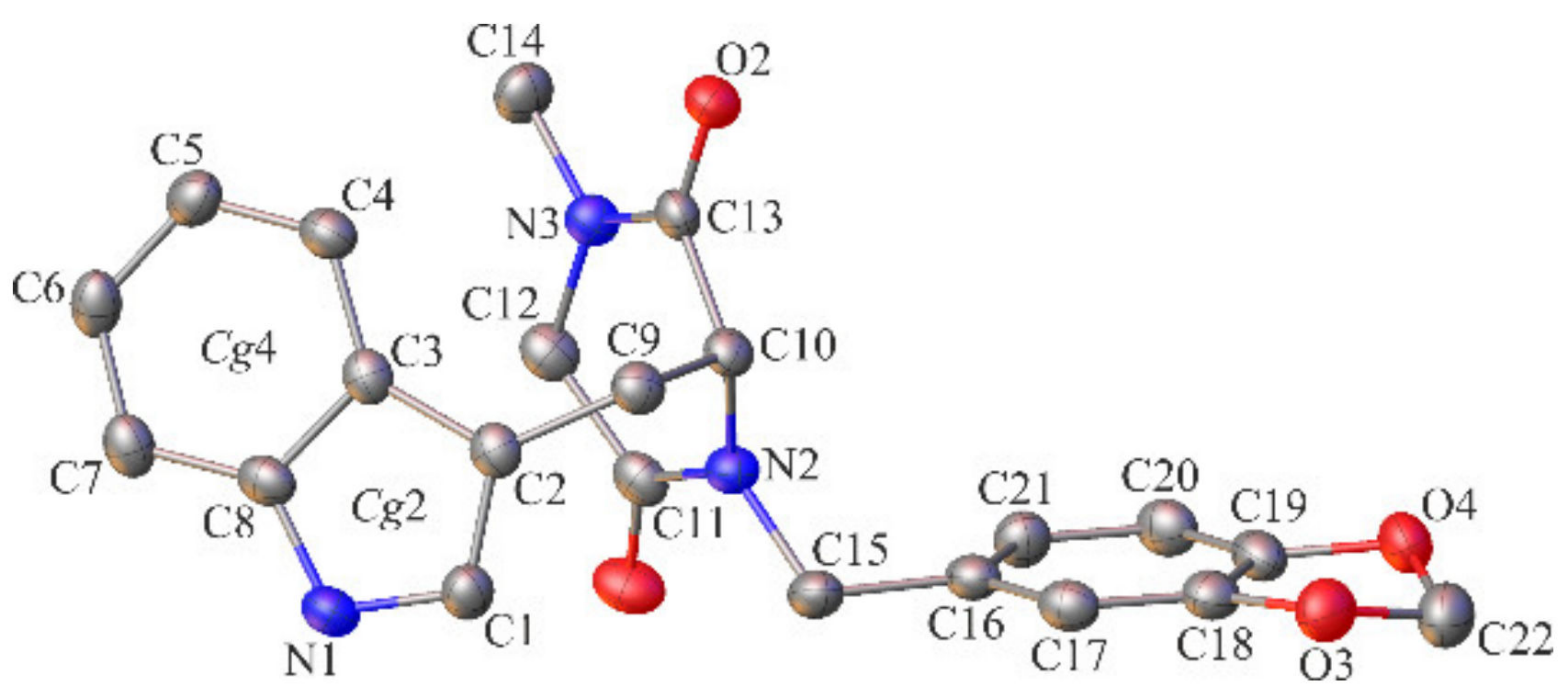
Asymmetric unit of the crystal lattice of **6a** with crystallographic numbering. Displacement ellipsoids are drawn at the 50% probability level and the H-atoms were omitted for clarity. *Cg*2 and *Cg*4 denote the geometric centres of gravity of the aromatic rings delineated by the N1/C1–C3/C8 and C3–C8 atoms, respectively.

**Figure 7:**
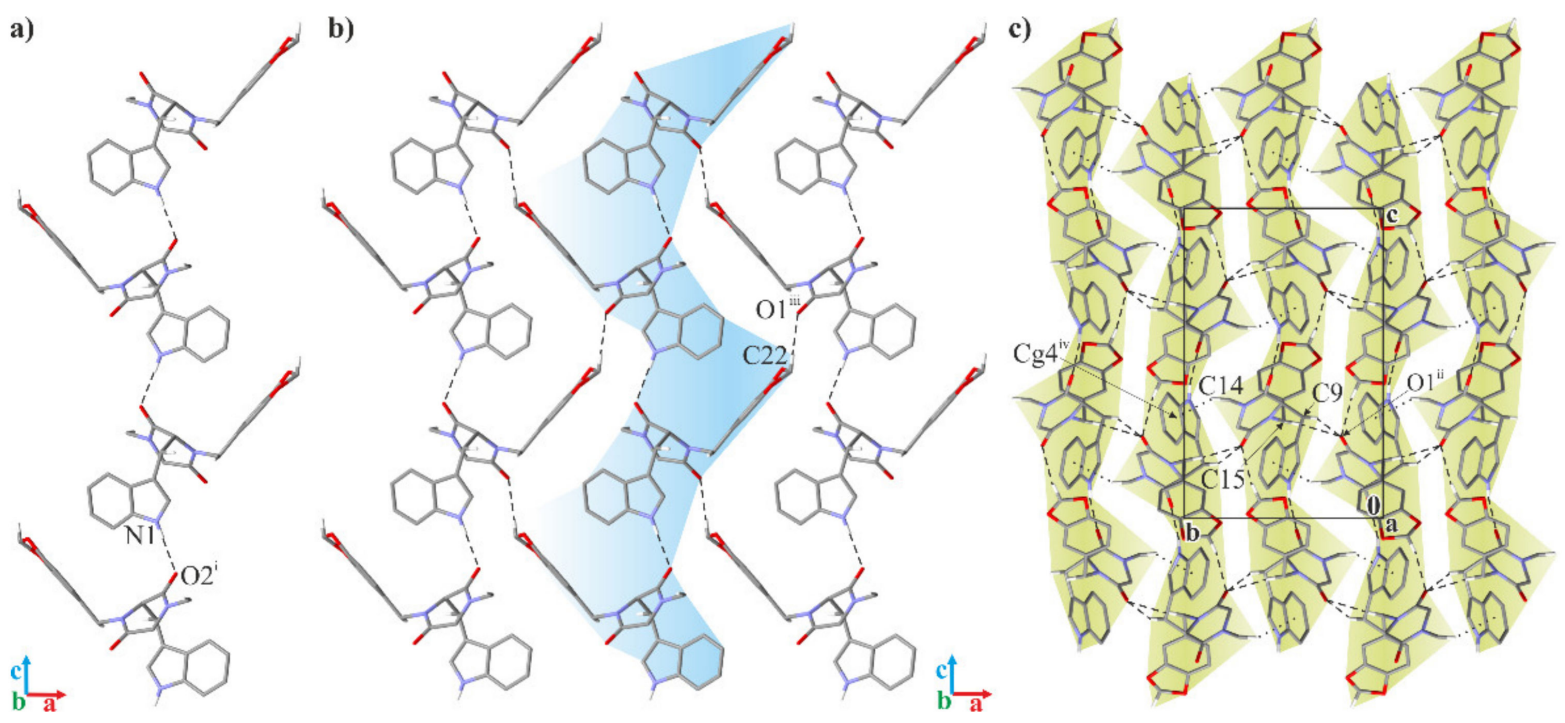
Arrangement of molecules of **6a** in the crystal, where: (**a**) chain of molecules running along [001] direction; (**b**) single layer of molecules spreading along (100) plane (single chain of molecules is highlighted in blue); (**c**) general view on layered supramolecular architecture of compound viewed along *a*-direction (single layers are highlighted in light green). The H-atoms not involved in intermolecular interactions have been omitted for clarity. The N–H⋯O and C–H⋯O hydrogen bonds are represented by dashed lines, while the C–H⋯π contacts by dotted lines. Symmetry codes: (i) −*x* + 1/2, −*y* + 1, *z* − 1/2; (ii) −*x* + 1, *y* − 1/2, −*z* + 1/2; (iii) −*x* + 3/2, −*y* + 1, *z* +1/2; (iv) −*x*, *y* + 1/2, −*z* + 1/2.

**Figure 8:**
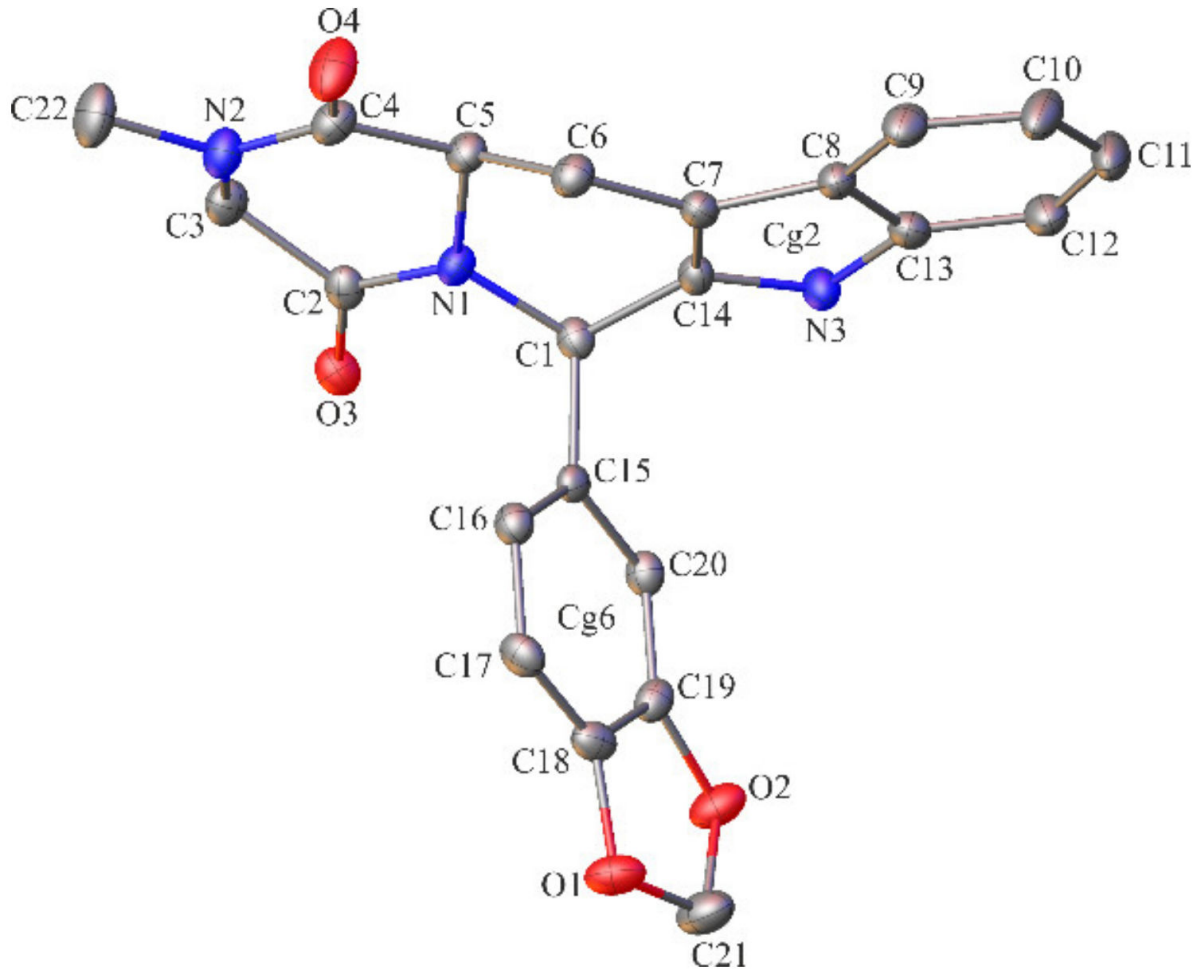
Asymmetric unit of the crystal lattice **1** with crystallographic numbering. Displacement ellipsoids are drawn at the 50% probability level and the H-atoms were omitted for clarity. *Cg*2 and *Cg*6 denote the geometric centres of gravity of the aromatic rings delineated by the N3/C13/C8–C7/C14 and C15–C20 atoms, respectively.

**Figure 9:**
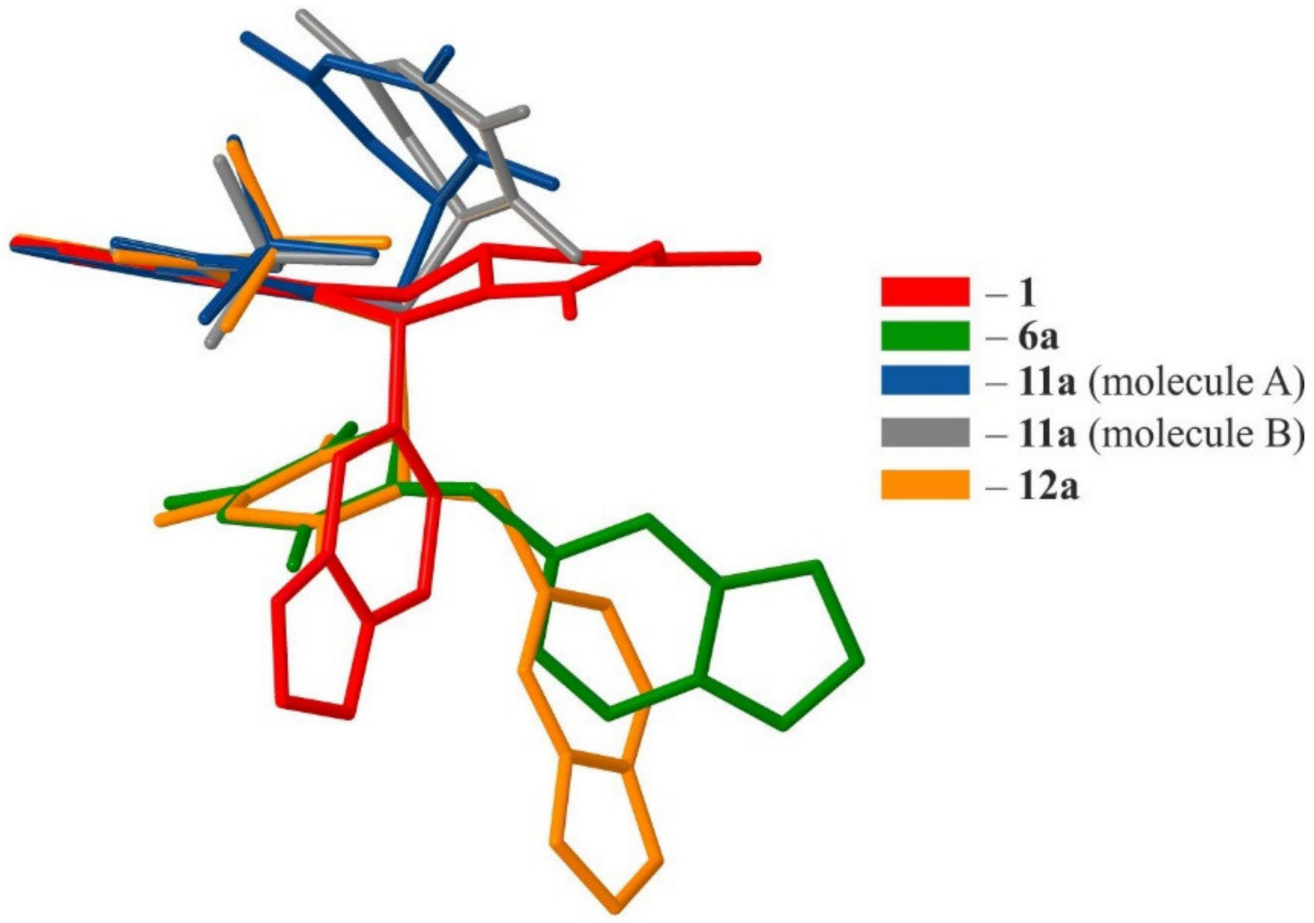
Superimposed molecules of **1**, **6a**, **11a** and **12a**. The H-atoms were omitted for clarity.

**Figure 10:**
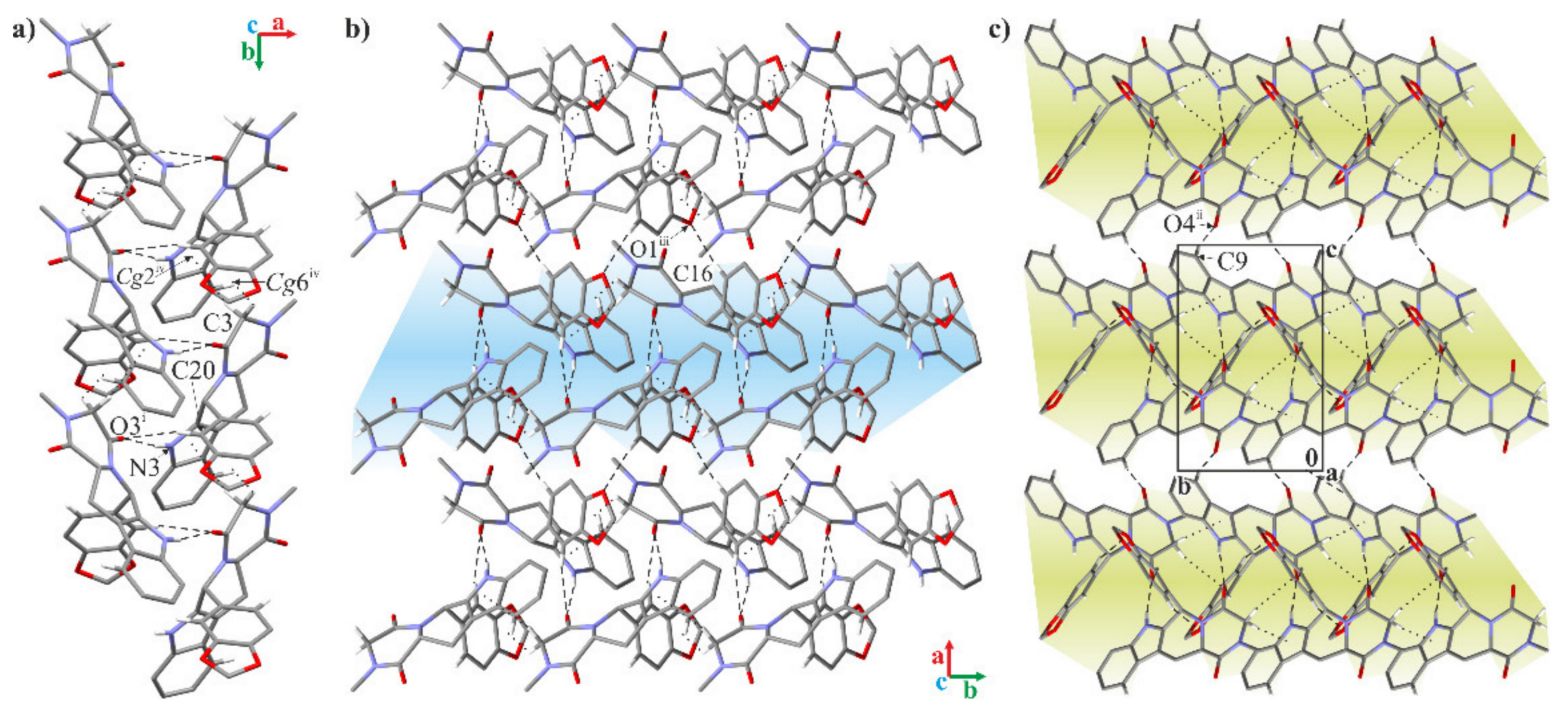
Arrangement of molecules of **1** in the crystal, where: (**a**) chain of molecules running along [010] direction; (**b**) single layer of molecules spreading along (001) plane (single chain of molecules is highlighted in blue); (**c**) general view on layered supramolecular architecture of compound viewed along *a*-direction (single layers are highlighted in light green). The H-atoms not involved in intermolecular interactions have been omitted for clarity. The N–H⋯O and C–H⋯O hydrogen bonds are represented by dashed lines, while the C–H⋯π contacts by dotted lines. Symmetry codes: (i) −*x* + 1/2, −*y* + 1, *z* − 1/2; (ii) −*x* + 1, *y* − 1/2, −*z* + 1/2; (iii) −*x* + 3/2, −*y* + 1, *z* +1/2; (iv) −*x*, *y* + 1/2, −*z* + 1/2.

The identity of the investigated compounds was confirmed by single-crystal X-Ray diffraction analysis (Figure 2, Figure 3, Figure 4, Figure 5, Figure 6, Figure 7, Figure 8, Figure 9 and Figure 10). It turned out that they were crystallizing in the monoclinic *P*2_1_ (**11a** and **1**) or orthorhombic *P*2_1_2_1_2_1_ (**12a** and **6a**) space groups with one (**1**, **6a**, **12a**) or two (**11a**) molecules of compound in the asymmetric unit of the crystal lattice (Figure 2a, Figure 4, Figure 6 and Figure 8). The details of crystallographic data and the refinement parameters are summarized in Table S1. The full list of values of bond lengths, valence and torsion angles can be found in the Supporting Information (Tables S2–S13).

The alignment of structural skeletons of both molecules comprising the asymmetric unit of **11a** (Figure 2b) reveals that their geometry is different. The main difference is related with the spatial orientation of the 3,6-dioxopiperazinyl moieties relative to the indole ring system. The mean-quadratic planes delineated by non-hydrogen atoms of mentioned fragments are inclined to themselves by the angle of 35.7(2)° and 44.1(2)° in case of molecules A and B, respectively. A close look at Figure 2b reveals also that *t*-butyl fragments in A and B are slightly twisted relative to each other. In both aforementioned molecules, the carbonyl group of the *t*-butylcarboxylate moiety is coplanar with the indole ring system. Specific molecular geometry is causing the appearance of several weak intramolecular C–H⋯O, C–H⋯π and C=O⋯π contacts (Tables S14–S16).

In the crystal, adjacent molecules of **11a** are involved in a dense network of N–H⋯O (d(D⋯A) = 2.908(6)–2.919(6) Å; <(D–H⋯A) = 154(7)–157(6)°, Table S14) and C–H⋯O (d(D⋯A) = 2.901(6)–3.459(7) Å; <(D–H⋯A) = 114–163°, Table S14) hydrogen bonds that are leading to the rise of infinite layers spreading along the (001) plane (Figure 3a). Molecules in these layers are arranged in a characteristic ‘herring-bone’ manner (Figure 2b). The whole crystal network is stabilized by the weak C–H⋯π contacts (d(D⋯A) = 3.490(6)–3.540(6) Å; <(D–H⋯A) = 122–126°, Table S15) involving neighboring molecules in adjacent layers (Figure 3b).

In case of **12a**, the mean-quadratic planes of indole and 3,6-dioxopiperazinyl in the molecule are inclined to each other by a 33.72(19)° angle, while the 3,6-piperazinyl and 5-methylbenzo[*d*][1,3]dioxazole fragments are oriented nearly perpendicular to themselves. The angle between mean planes defined by the non-hydrogen atoms of above moieties is 80.97(17)°. As in the case of **11a**, here also intramolecular C–H⋯O and C–H⋯π contacts were found (Tables S17 and S18).

Adjacent molecules of **12a** in the crystal are held together via the network of bifurcated C–H⋯O hydrogen bonds (d(D⋯A) = 3.418(7)–3.452(7) Å; <(D–H⋯A) = 146–163°, Table S17) into infinite chains running along the [001] direction (Figure 5a). The molecules from neighboring chains are further interacting by themselves by the network of weak C–H⋯O hydrogen bonds (d(D⋯A) = 2.987(8) Å; <(D–H⋯A) = 117°, Table S17) and C–H⋯π contacts (d(D⋯A) = 3.362(9) Å; <(D–H⋯A) = 135°, Table S18), which is leading to the rise of infinite layers spreading along the (100) plane (Figure 5b). Whole layered supramolecular architecture is stabilized by the presence of weak C–H⋯π contacts (d(D⋯A) = 3.896(7) Å; <(D–H⋯A) = 170°, Table S18) between adjacent molecules in neighboring layers (Figure 5c).

The mean-quadratic planes of indole and benzo[*d*][1,3]dioxazole moieties in case of the molecule of **6a** are inclined by the angles of 36.15(9) and 93.08(8)° relative to the 3,6-dioxopiperazinyl moiety. A detailed analysis of molecular interactions allowed us to identify the existence of one weak intramolecular C–H⋯π contact (Table S20).

In the crystal of **6a**, neighboring molecules of the investigated compound are incorporated in the N–H⋯O hydrogen bonds (d(D⋯A) = 2.895(3) Å; <(D–H⋯A) = 171(3)°, Table S19), which is leading to the appearance of an infinite chain running along [001] direction (Figure 7a). Adjacent molecules in neighboring supramolecular assemblies of this type are then interacting by themselves via the weak C–H⋯O hydrogen bonds (d(D⋯A) = 3.254(4) Å; <(D–H⋯A) = 170°, Table S19). This results in the formation of infinite layers spreading along (100) plane (Figure 7b). Whole, layered supramolecular architecture of **6a** is stabilized by the presence of weak C–H⋯π contacts (d(D⋯A) = 3.451(4) Å; <(D–H⋯A) = 127°, Table S20) and bifurcated C–H⋯O hydrogen bonds (d(D⋯A) = 3.345(4)–3.385(4) Å; <(D–H⋯A) = 155–171°, Table S19) involving adjacent molecules in neighboring layers (Figure 7c).

Molecule **1** (tadalafil) consists of the lactam and indole moieties (Figure 8). The unit cell parameters and geometric features describing the molecular skeleton of the compound presented in this work are in good agreement with the structure of tadalafil previously reported by Daugan et al. [2]. Although in our research diffractometric measurements of the investigated system were conducted at 100(2) K instead of room temperature, no phase transition was observed.

The alignment of tadalafil molecule with the molecules of three compounds, of which the crystal structures are described above (**6a**, **11a** and **12a**), highlights the existence of significant structural differences between them. A close look at Figure 9 reveals clearly that diketopiperazine moiety in **6a**, **11a** and **12a** is much more bent above or below the plane of the indole unit compared to that what is observed within the rigid pyrazinopyridoindole core in **1**. It can be seen also that all derivatives with disjointed diketopiperazine and indole moieties indicate noticeable conformational flexibility.

In the crystal, adjacent molecules of **1** are held together by the network of N–H⋯O (d(D⋯A) = 2.968(3) Å; <(D–H⋯A) = 171(4)°, Table S21) and C–H⋯O (d(D⋯A) = 3.373(4) Å; <(D–H⋯A) = 154°, Table S21) hydrogen bonds into infinite chains running along [010] direction (Figure 10a). The above-mentioned supramolecular assembly is stabilized by the presence of weak intermolecular C–H⋯π contacts (d(D⋯A) = 3.550(3)–3.690(3) Å; <(D–H⋯A) = 139–167°, Table S22). Neighboring chains further interact by weak C–H⋯O hydrogen bonds (d(D⋯A) = 3.176(4) Å; <(D–H⋯A) = 151°, Table S21), which results in an arising of an infinite layer of molecules spreading along the (001) plane (Figure 10b). Finally, layered supramolecular architecture of **1** is stabilized via the network of weak C–H⋯O hydrogen bonds (d(D⋯A) = 3.476(5) Å; <(D–H⋯A) = 171(4)°, Table S21) engaging adjacent molecules of compounds in neighboring layers (Figure 10c).

### 2.3. Cytotoxic Effect

**Figure 11:**
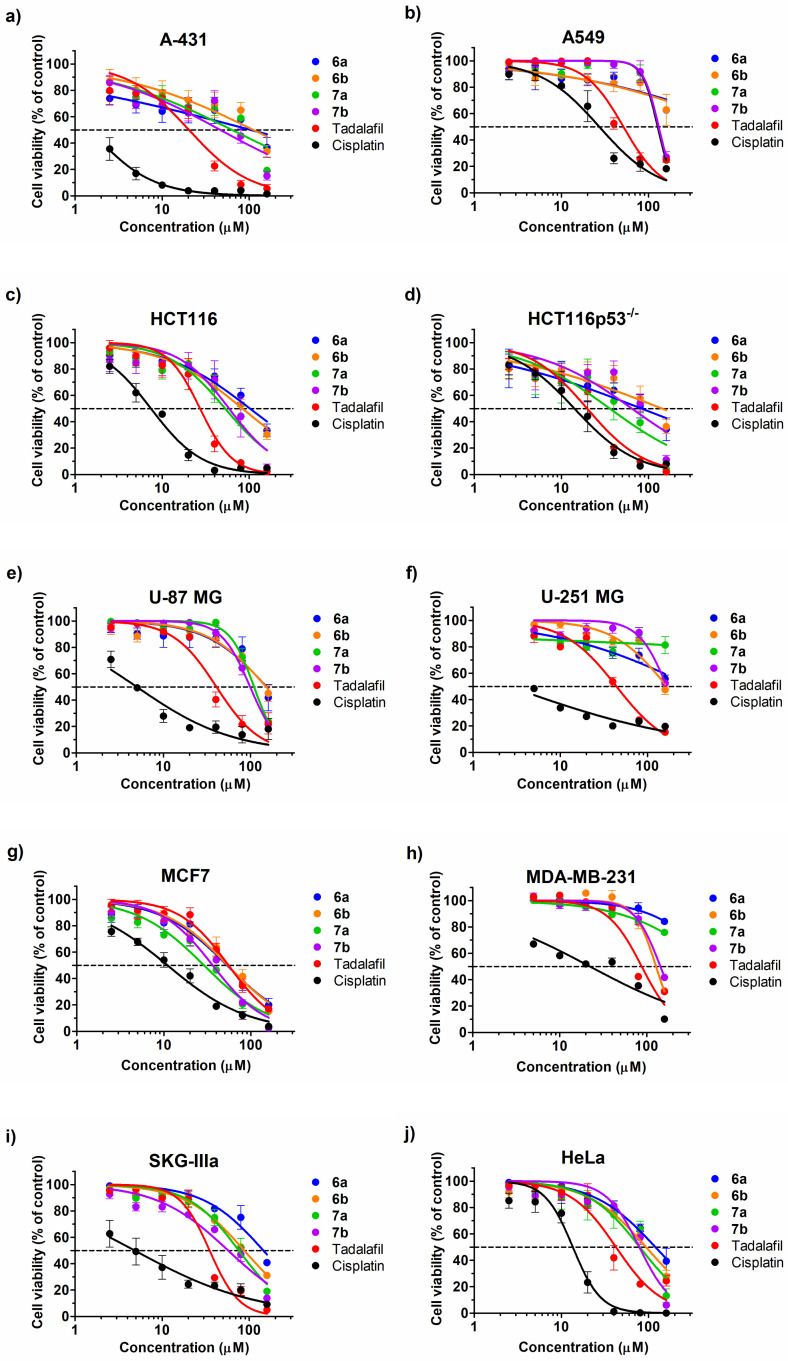

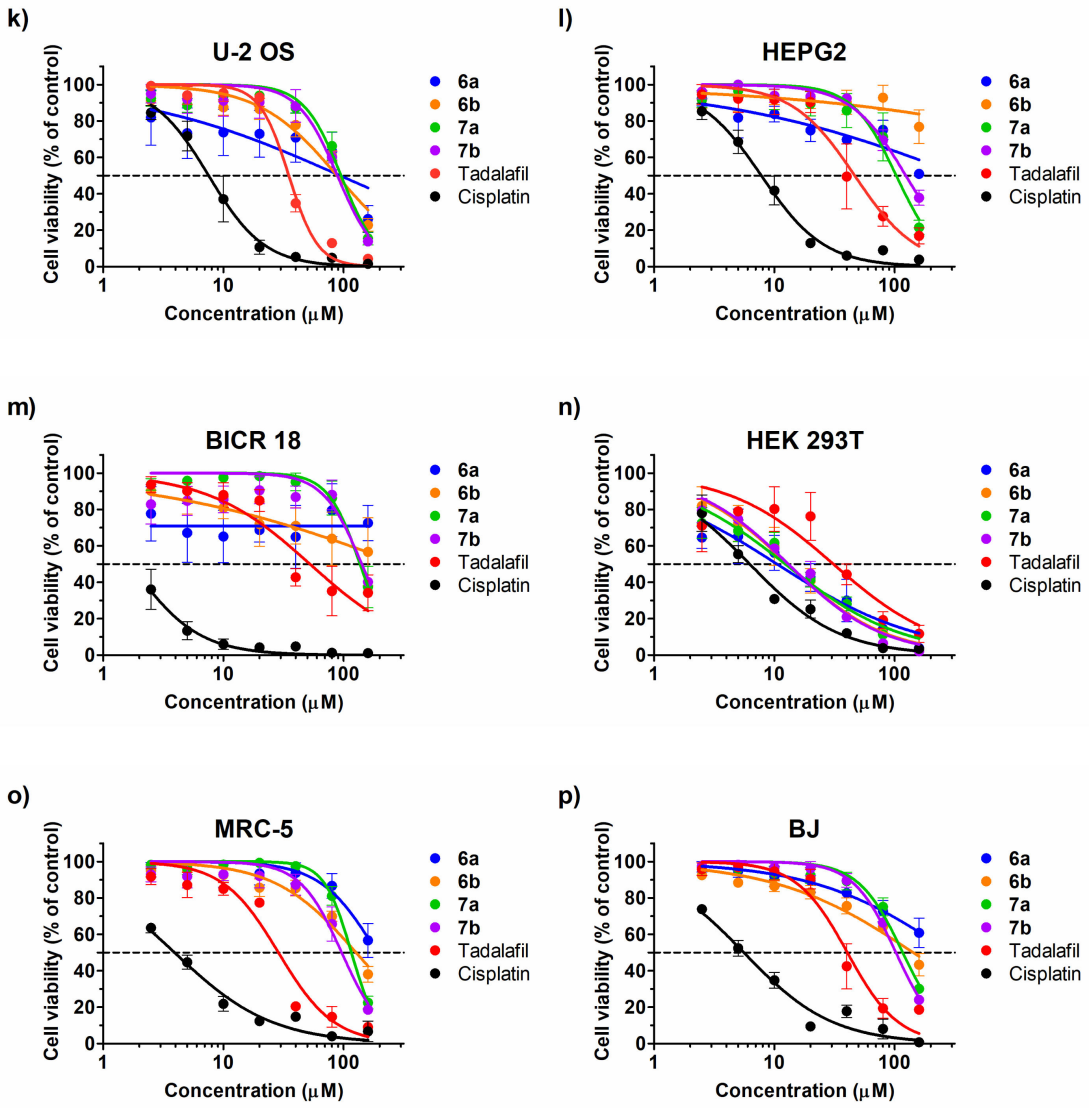
Viability of selected human cell lines in response to tadalafil and its analogues **6a,b**, **7a,b**, and cisplatin evaluated with MTT metabolism assay. A-431 (**a**), A549 (**b**), HCT116 (**c**), HCT116p53^−/−^ (**d**), U-87 MG (**e**), U-251 MG (**f**), MCF7 (**g**), MDA-MB-231 (**h**), SKG-IIIa (**i**), HeLa (**j**), U-2 OS (**k**), HEPG2 (**l**), BICR 18 (**m**), HEK 293T (**n**), MRC-5 (**o**) and BJ (**p**). Cells were incubated with increasing concentrations (range = 1.25–160 μM) of tadalafil, compounds **6a,b**, **7a,b**, and cisplatin for 72 h. Percentage of cell viability inhibition was calculated in comparison to cells treated with DMSO only (regarded as a control). Each point of curves represents the mean ± SD of at least three independent experiments done in triplicate. Nonlinear fit of the dose-log response curves was done in GraphPad Prism 5.03.

The synthesized compounds **6a,b** and **7a,b**, together with tadalafil, were tested in vitro to determine their cytotoxic activity against sixteen cancer cell lines: epidermoid carcinoma A-431, lung carcinoma A549, colorectal carcinoma HCT116 and HCT116p53^−/−^, glioblastoma U-87 MG and U-251, breast adenocarcinoma MCF7 and MDA-MB-231, cervical squamous cell carcinoma SKG-IIIa, cervical adenocarcinoma HeLa, bone osteosarcoma U-2 OS, hepatocellular carcinoma HEPG2, larynx squamous cell carcinoma BICR 18 and non-cancer cell lines: kidney HEK 293T (expresses a mutant version of SV40 large T antigen) and normal fibroblasts MRC-5 and BJ. Additionally, the anticancer drug, cisplatin, was used as a positive control. The viability results and obtained half maximal inhibitory concentrations (IC_50_) are shown in Figure 11 and Table 1. The cytotoxicity of each derivative of the tadalafil was different depending on the dose and type of treated cell line. The highest toxicity was observed for all analogues in HEK 293T and MCF7 cell lines. Only these lines were more sensitive to **6a,b** and **7a,b** than to tadalafil (Figure 11g,n, and Table 1, bold digits). For most cell lines, compounds **7a,b** were slightly more toxic in comparison to **6a,b**. The least cytotoxic effect was observed for U-251 MG cells, where the IC_50_ values for all studied analogues were above 160 μM (Figure 11f and Table 1). Overall, the results clearly demonstrate that only analogues **7a** and **7b**, where IC_50_ values were only about three times higher in comparison to cisplatin (Table 1) and in the highest two concentrations dropped to a similar percentage of survival as for this drug (Figure 11g); they could be considered as candidates for selective anticancer treatment. Since the mechanism of action of the tested compounds is not determined and the obtained cytotoxic effect is low, it is difficult to precisely explain the greater cytotoxic effect of the bromine substituted compounds **7a** and **7b** than their analogues **6a** and **6b**. It is known, however, that the presence of bromine in the ligand can cause the formation of additional halogen bonds at the active site of the protein and thus increase the binding of the ligand to the protein. Bromine substituted compounds may also be better suited to the hydrophobic pocket in the active side of the protein.

### 2.4. Enzyme Inhibition

Additionally, for all four compounds and tadalafil, as the control, the inhibitory activities against PDE5 were determined with the aid of a colorimetric PDE Activity Assay Kit (Abcam). A high concentration of cGMP substrate was used (200 μM) due to the moderate sensitivity of the aforementioned assay. This concentration of cGMP was approximately 100 times above the apparent Km, so the direct comparison with earlier reported tadalafil IC_50_ values was impossible. To obtain direct binding affinity (independent of substrate concentration), we converted measured IC_50_ value into inhibition constant (Ki) using the Cheng–Prusoff equation [47], assuming Km = 2.0 ± 0.5 μM [48]. The results are shown in Figure S41 and Table 2.

Calculated binding affinity for tadalafil is similar to earlier reported IC_50_ values for PDE5 that vary from ~1–2 nM [49,50]. Unfortunately, none of these newly synthesized analogs exhibit significant inhibitory activity against human PDE5, since the most potent of them—**7a** is over 300 less effective than tadalafil. However, we can observe an increase in the PDE5 inhibitory activity for brominated analogs **7a,b** compared to unsubstituted (**6a,6b**). Moreover, the unsubstituted (*S*) isomer **6a** is a significantly less effective inhibitor than the (*R*) isomer **6a** and the same tendency is observed for their halogenated counterparts.

### 2.5. Antiplatelet Activity of the Tested Compounds

**Figure 12:**
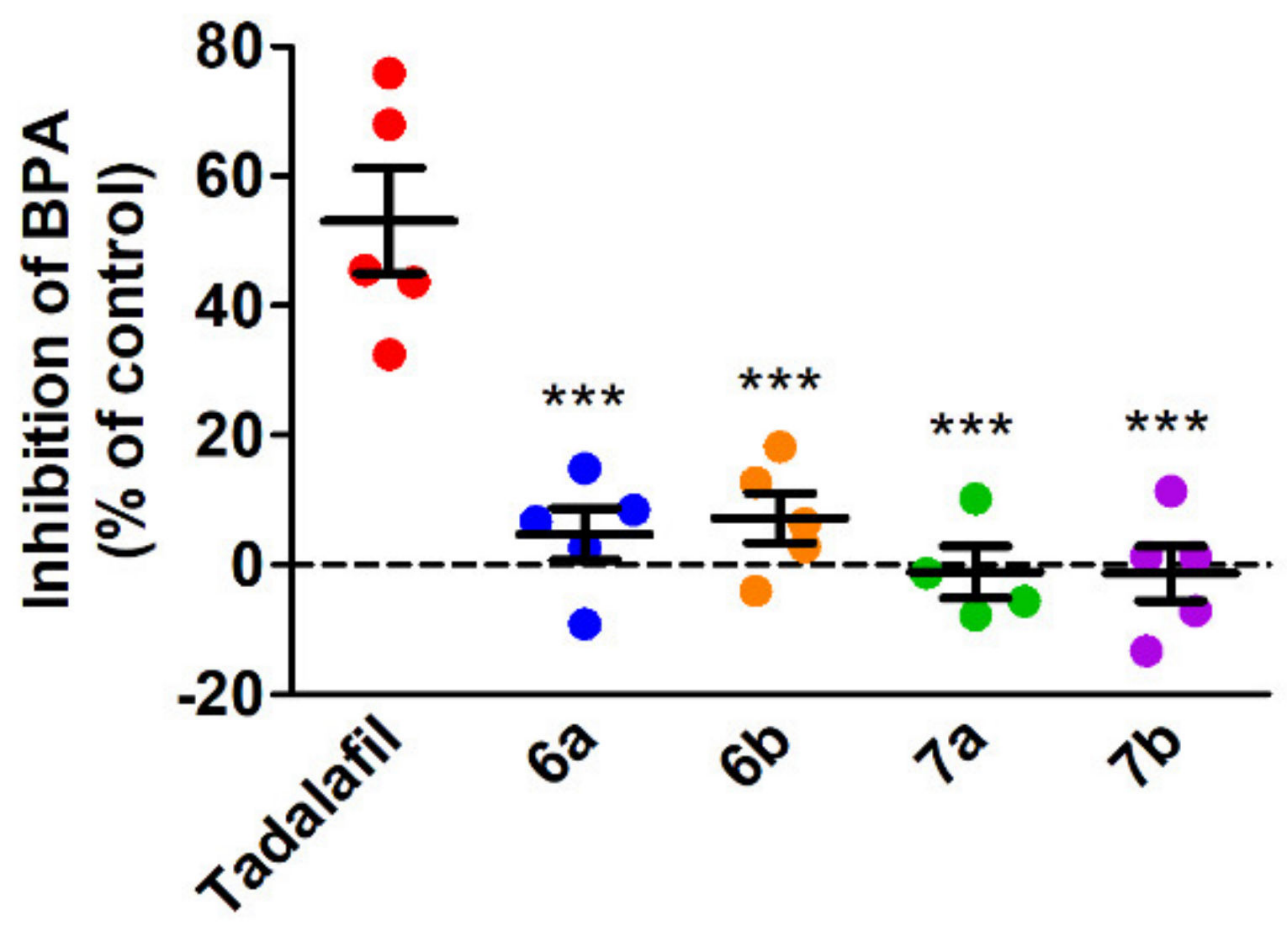
Inhibition of blood platelet aggregation (BPA) by tadalafil and its analogues in the presence of DEA-NO evaluated by light transmittance aggregometry. Platelet rich plasma was preincubated for 5 min with tadalafil (10 μM) or its analogues, **6a,b**, **7a,b** (10 μM). Shortly prior to measurement, 500 nM DEA-NO was added, followed by 2 μg/mL collagen. Percentage of inhibition of platelet response was calculated in comparison to a sample treated with DMSO and DEA-NO on the basis of maximal value of platelet aggregation. Black horizontal lines on data points show means and standard deviations. Statistical analysis was performed via one-way analysis of variance with Tukey’s multiple-comparison test (***, *p* < 0.001).

Nitric oxide is one of the main physiological antiplatelet molecules secreted by vascular endothelium. It stimulates intraplatelet production of cGMP. This secondary messenger is then decomposed in platelets by the activity of two out of three PDE isoforms which are detected in platelets, namely by PDE2 and PDE5, while the other isoform (PDE3) is mostly involved in cAMP hydrolysis [51]. Inhibitors of these phosphodiesterases increase intraplatelet concentration of cGMP and thus potentiate the antiplatelet effect of NO [52]. Sildenafil has been shown to possess such a potentiating effect in a model of blood platelet aggregation [53]. Present studies were aimed to verify antiplatelet activity of four newly synthesized PDE5 inhibitors with the use of tadalafil as a reference. Antiplatelet activity of the compounds was tested by means of light transmittance aggregometry in platelet-rich plasma stimulated with collagen in presence of a donor of nitric oxide (DEA-NO). As shown in Figure 12, tadalafil in presence of DEA-NO inhibited platelet aggregation by approx. 50%, while none of the tested analogues presented significant antiplatelet activity.

### 2.6. Toxicity against Caenorhabditis Elegans

We also performed an investigation on the toxicological effect of synthesized compounds on *Caenorhabditis elegans*. In the highest applied concentration—160 μM, **6a,b** and **7a,b** did not exhibit any toxicity in *C. elegans* after 48 h exposure.

## 3. Materials and Methods

### 3.1. Chemistry

#### 3.1.1. General

Commercially available chemicals were of reagent grade and used as received. Purification of the compounds was performed by column chromatography on silica gel 60 M (0.040–0.063 mm, E. Merck, Darmstadt, Germany). Thin layer chromatography (TLC), using silica gel plates (Kieselgel 60F254, E.Merck), was used to monitor reaction progress. A B-540 Melting Point apparatus (Büchi, New Castle, DE, USA) was used to measure melting points. The one-dimensional ^1^H, and ^13^C, together with two-dimensional ^1^H-^13^C hetero-correlation NMR experiments, were performed in DMSO-*d*_6_ utilizing Varian Inova 500 NMR spectrometer (Agilent Inc. PaloAlto, CA, USA) at 298 K. The ^1^H and ^13^C chemical shifts were reported relative to the SiMe_4_ (TMS) internal reference. The resonance assignments were achieved based on peak multiplicity and peak integration of recorded ^1^H data, supplemented with ^13^C and ^1^H-^13^C hetero-correlation spectra. Multiplets were assigned as *s* (singlet), *d* (doublet), *t* (triplet), *dd* (doublet of doublet), *ddd* (doublet of doublet of doublet) and *m* (multiplet). An LTQ Orbitrap Velos instrument (Thermo Scientific, Waltham, MA, USA) located at the Mass Spectrometry Laboratory of the Institute of Biochemistry and Biophysics PAS (Warsaw, Poland) was used to record high resolution mass spectra. A 6200 FT/IR spectrometer (Jasco, Easton, MD, USA) at the Laboratory of Optical Spectroscopy (Institute of Organic Chemistry PAS, Warsaw, Poland) was used to record IR spectra.

#### 3.1.2. Synthetic Procedures and Analytical Data

##### The Synthesis of tert-butyl (*R*)-(**11a**) and (*S*)-3-((4-methyl-3,6-dioxopiperazin-2-yl)methyl)-1*H*-indole-1-carboxylate (**11b**)

The 3.5 g of Fmoc-d-Trp(Boc)-OH (**8a**) or Fmoc-l-Trp(Boc)-OH (**8b**) (6.65 mmol, 1 equiv.) was dissolved in 100 mL of methylene chloride and 2.57 g of Sar(OBzl) *TsOH (7.31 mmol, 1.1 equiv., **9**) followed by 3.41 g of PyBrop (7.31 mmol, 1.1 equiv.) and 2.33 mL of DIPEA (13.29 mmol, 2.0 equiv.) were added. The reaction mixture was stirred at room temperature for 18 h, and the progress of reaction was monitored by mass spectrometry. Then, the solvent was evaporated and crude products **10a** and **10b** were dissolved in 100 mL of 25% piperidine in DMF. The reaction mixture was stirred at room temperature for 18 h. Then, when the volatiles were evaporated under the reduced pressure, the residue was dissolved in acetone and finally evaporated with silica gel. The product was purified by column chromatography using ethyl acetate (removal of less polar by-products) followed by ethyl acetate:acetone 7:3 (isolation of main product). Yield: 1.13 g of **11a** and 1.15 g of **11b** (48%). M.p.: 185.5–186.5 °C ^1^H NMR (CDCl_3_, 500 MHz) for **11a**: 8.14 (d, 1H, *J* = 8.0 Hz, H_Ar_); 7.56–7.52 (m, 1H, H_Ar_); 7.49 (s, 1H, H_Ar_); 7.36–7.30 (m, 1H, H_Ar_); 7.28–7.21 (m, 1H, H_Ar_); 6.41 (s, 1H, NH), 4.34–4.27 (m, 1H, CH); 3.71 (dd, 1H, *J* = 1.0, *J′* = 18.0 Hz, CH_2_); 3.44 (dd, 1H, *J* = 1.0, *J′* = 18.0 Hz, CH_2_); 3.39 (ddd, 1H, *J* = 1.0, *J′* = 3.5, *J″* = 14.5 Hz, CH_2_); 3.19–3.12 (m, 1H, CH_2_); 2.84 (s, 3H, Me), 1.67 (s, 9H, *t-*Bu). ^13^C NMR (CDCl_3_,125 MHz) for **11a**: 165.7, 165.4, 149.5, 135.6, 129.7, 125.4, 125.0, 122.9, 119.2, 115.5, 114.2, 84.2, 55.1, 51.5, 33.9, 30.7, 28.3; HRMS (ESI) **11a:** *m/z* [2M + H]^+^ calculated for C_38_H_47_N_6_O_8_: 715.34499, found: 715.34570; IR (KBr) **11a:** cm^−1^ 3477, 3265, 2981, 2936, 1737, 1654, 1565, 1457, 1377, 1339, 1319, 1271, 1228, 1201, 1158, 1106, 1081, 1025;

##### The Synthesis of tert-butyl (*R*)-(**12a**) and (*S*)-3-((1-(benzo[*d*][1,3]dioxol-5-ylmethyl)-4-methyl-3,6-dioxopiperazin-2-yl)methyl)-1*H*-indole-1-carboxylate (**12b**)

The 396 mg of *tert*-butyl (*R*)-3-((4-methyl-3,6-dioxopiperazin-2-yl)methyl)-1*H*-indole-1-carboxylate (**11a**) or *tert*-butyl (*S*)-3-((4-methyl-3,6-dioxopiperazin-2-yl)methyl)-1*H*-indole-1-carboxylate (**11b**) (1.11 mmol, 1 equiv.) were dissolved in 15 mL of DMF. After dissolution of **11a-b**, 722 mg (2.22 mmol, 2 equiv.) of Cs_2_CO_3_, followed by 250 mg (1.16 mmol, 1.05 equiv.) of 5-(bromomethyl)benzo[*d*][1,3]dioxole were added and reaction mixture was stirred at room temperature for 18 h. The next day, the obtained solution was diluted with 100 mL of ethyl acetate, washed with water (3 × 100 mL), dried over magnesium sulfate and finally evaporated with silica gel. The product was purified by column chromatography using hexane: ethyl acetate 1:1. Yield: 461 mg (84%) for **12a** and 369 mg (68%) for **12b**. M.p.: 195.0–196.0 °C ^1^H NMR (CDCl_3_, 500 MHz) for **12a**: 8.12 (d, 1H, *J* = 8.0 Hz, H_Ar_); 7.48–7.43 (m, 1H, H_Ar_); 7.34–7.28 (m, 2H, H_Ar_); 7.26–7.22 (m, 1H, H_Ar_); 6.78–6.71 (m, 3H, H_Ar_); 5.96 (dd, 2H, *J* = 1.5, *J′* = 4.0 Hz, CH_2_); 5.29 (d, 1H, *J* = 14.5 Hz, CH_2_); 4.02 (t, 1H, *J* = 4.0 Hz, CH); 3.92 (d, 1H, *J* = 14.5 Hz, CH_2_); 3.43–3.35 (m, 2H, CH_2_); 3.17 (dd, 1H, *J* = 5.0, *J′* = 15.0 Hz, CH_2_); 2.69 (d, 1H, *J* = 17.0 Hz, CH_2_); 2.56 (s, 3H, Me), 1.68(s, 9H, *t-*Bu). ^13^C NMR (CDCl_3_,125 MHz) for **12a**: 166.5, 164.2, 149.5, 148.4, 147.8, 135.5, 129.8, 129.2, 125.6, 125.1, 122.9, 122.4, 119.4, 115.4, 113.8, 109.1, 108.6, 101.4, 84.3, 59.1, 51.4, 47.0, 33.5, 28.3, 27.3; HRMS (ESI) **12a:** *m/z* [M^+^ calculated for C_27_H_30_N_3_O_6_: 492.21291, found: 492.21292; IR (KBr) **12a:** cm^−1^ 2978, 2927, 2898, 1742, 1658, 1494, 1472, 1450, 1373, 1324, 1296, 1252, 1156, 1098, 1079, 1038;

##### The Synthesis of tert-butyl (*R*)-(**13a**) and (*S*)-3-((1-((6-bromobenzo[*d*][1,3]dioxol-5-yl)methyl)-4-methyl-3,6-dioxopiperazin-2-yl) methyl)-1*H*-indole-1-carboxylate (**13b**)

The 354 mg of *tert*-butyl (*R*)-3-((4-methyl-3,6-dioxopiperazin-2-yl)methyl)-1*H*-indole-1-carboxylate (**11a**) or *tert*-butyl (*S*)-3-((4-methyl-3,6-dioxopiperazin-2-yl)methyl)-1*H*-indole-1-carboxylate (**11b**) (0.99 mmol, 1 equiv.) was dissolved in 15 mL of DMF. After dissolution of **11a,b**, 646 mg (1.98 mmol, 2 equiv.) of Cs_2_CO_3_, followed by 306 mg of 5-bromo-6-(bromomethyl)benzo[*d*][1,3]dioxole (1.04 mmol, 1.05 equiv.) were added and reaction mixture was stirred at room temperature for 18 h. The next day, the obtained solution was diluted with 100 mL of ethyl acetate, washed with water (3 × 100 mL), dried over magnesium sulfate and finally evaporated with silica gel. The product was purified by column chromatography using hexane: ethyl acetate 1:1. Yield: 418 mg (74%) for **13a** and 525 mg (93%) for **13b**. M.p.: 94.5–95.5 °C ^1^H NMR (CDCl_3_, 500 MHz) for **13a**: 8.12 (d, 1H, *J* = 8.0 Hz, H_Ar_); 7.50–7.43 (m, 1H, H_Ar_); 7.37–7.28 (m, 2H, H_Ar_); 7.26–7.22 (m, 1H, H_Ar_); 7.01 (s, 1H, H_Ar_); 6.84 (s, 1H, H_Ar_); 5.99 (dd, 2H, *J* = 1.5 Hz, *J′* = 5.5 Hz, CH_2_); 5.32 (d, 1H, *J* = 15.0 Hz, CH_2_); 4.28 (d, 1H, *J* = 15.0 Hz, CH_2_); 4.21 (dd, 1H, *J* = 3.5 Hz, *J′* = 4.5 Hz, CH); 3.44 (ddd, 1H, *J* = 1.0 Hz, *J′* = 4.5 Hz, *J″* = 15.0 Hz CH_2_); 3.37 (d, 1H, *J* = 17.5 Hz, CH_2_); 3.29 (dd, 1H, *J* = 4.5 Hz, *J′*= 15.0 Hz CH_2_); 2.62 (d, 1H, *J* = 17.5 Hz, CH_2_); 2.55 (s, 3H, Me); 1.68 (s, 9H, *t*-Bu); ^13^C NMR (CDCl_3_,125 MHz) for **13a**: 166.5, 164.5, 149.5, 148.6, 148.2, 135.4, 129.8, 128.0, 125.7, 125.0, 122.9, 119.4, 115.4, 114.9, 113.7, 112.8, 110.1, 102.2, 84.3, 59.3, 51.3, 46.2, 33.5, 28.3, 27.4; HRMS (ESI) **13a**: *m/z* [M + H]^+^ calculated for C_27_H_29_BrN_3_O_6_: 570.12342, 572.12138, found: 570.12365, 572.12133; IR (KBr) for **13a**: cm^−1^ 3457, 3314, 3111, 3050, 2977, 2928, 2076, 1734, 1667, 1567, 1504, 1479, 1454, 1407, 1371, 1330, 1256, 1232, 1197, 1156, 1113, 1083, 1035;

##### The Synthesis of (*R*)-(**6a**) and (*S*)-3-((1*H*-indol-3-yl)methyl)-4-(benzo[*d*][1,3]dioxol-5-ylmethyl)-1-methylpiperazine-2,5-dione (**6b**)

Moreover, 277 mg of Boc-protected intermediate **12a** and 321 mg of Boc-protected intermediate **12b** were dissolved in TFA:dichloromethane mixture (1:4 *v*/*v*, 20 mL) and stirred at room temperature for 18 h. The next day, reaction mixtures were diluted with toluene (200 mL), evaporated under the reduced pressure and co-evaporated with toluene (two times, 50 mL). The residues were dissolved in 30 mL of THF and finally evaporated with silica gel. The products were purified by column chromatography using hexane: ethyl acetate 1:3. Yields: 190 mg (86%) for **6a** and 192 mg (75%) for **6b**. M.p.: 195.5–196.5 °C **^1^H NMR** (CDCl_3_, 500 MHz) for **6a**: 8.63 (s, 1H, NH); 7.54–7.49 (m, 1H, H_Ar_); 7.36–7.31 (m, 1H, H_Ar_); 7.21–7.14 (m, 1H, H_Ar_); 7.14–7.08 (m, 1H, H_Ar_); 6.87 (d, 1H, *J* = 2.5 Hz, H_Ar_); 6.82–6.77 (m, 1H, H_Ar_); 6.77–6.74 (m, 2H, H_Ar_); 5.97–5.94 (m, 2H, CH_2_); 5.35 (d, 1H, *J* = 14.5 Hz, CH_2_); 4.18 (dd, 1H, *J* = 3.0 Hz, *J′* = 4.5 Hz, CH); 3.92 (d, 1H, *J* = 14.5 Hz, CH_2_); 3.51 (dd, 1H, *J* = 3.0 Hz, *J′* = 15.0 Hz, CH_2_); 3.29–3.17 (m, 2H, CH_2_); 2.44 (s, 3H, Me); 2.15 (d, 1H, *J* = 17.0 Hz, CH_2_); ^13^C NMR (CDCl_3_,125 MHz) for **6a**: 166.8, 165.0, 148.4, 147.7, 136.2, 129.2, 127.1, 124.4, 122.6, 122.3, 120.0, 119.2, 111.3, 109.0, 108.6, 108.5, 101.4, 59.5, 51.1, 46.7, 33.3, 27.20. HRMS (ESI) **6a**: *m/z* [M + H]^+^ calculated for C_22_H_22_N_3_O_4_: 392.16048, found: 392.16047; IR (KBr) **6a**: cm^−1^ 3262, 3048, 2979, 2896, 1658, 1488, 1439, 1408, 1331, 1285, 1245, 1193, 1159, 1130, 1100, 1033;

##### The Synthesis of (*R*)-(**7a**) and (*S*)-3-((1*H*-indol-3-yl)methyl)-4-((6-bromobenzo[*d*][1,3]dioxol-5-yl)methyl)-1-methyl-piperazine-2,5-dione (**7b**)

Overall, 338 mg of Boc-protected intermediate **13a** and 364 mg of Boc-protected intermediate **13b** were dissolved in TFA:dichloromethane mixture (1:4 *v*/*v*, 20 mL) and stirred at room temperature for 18 h. The next day, reaction mixtures were diluted with toluene (200 mL), evaporated under the reduced pressure and co-evaporated with toluene (two times, 50 mL). The residues were dissolved in 30 mL of THF and finally evaporated with silica gel. The products were purified by column chromatography using hexane: ethyl acetate 1:3. Yields: 265 mg (95%) for **7a** and 249 mg (83%) for **7b**. M.p.: 104.5–105.5 °C ^1^H NMR (CDCl_3_, 500 MHz) for **7a**: 8.44 (s, 1H, NH); 7.55–7.50 (m, 1H, H_Ar_); 7.38–7.33 (m, 1H, H_Ar_); 7.22–7.15 (m, 1H, H_Ar_); 7.15–7.10 (m, 1H, H_Ar_); 7.02 (s, 1H, H, H_Ar_); 6.93 (d, 1H, *J* = 2.5 Hz, H_Ar_); 6.83 (s, 1H, H_Ar_); 5.98 (dd, 2H, *J* = 1.5 Hz, *J′* = 4.5 Hz, CH_2_); 5.37 (d, 1H, *J* = 15.0 Hz, CH_2_); 4.26 (d, 1H, *J* = 15.0 Hz, CH_2_); 4.18 (dd, 1H, *J* = 2.5 Hz, *J′* = 4.5 Hz, CH); 3.56 (dd, 1H, *J* = 2.5 Hz, *J′* = 15.0 Hz, CH_2_); 3.32 (dd, 1H, *J* = 4.5 Hz, *J′* = 15.0 Hz, CH_2_); 3.23 (d, 1H, *J* = 17.0 Hz, CH_2_); 2.44 (s, 3H, Me); 2.12 (d, 1H, *J* = 17.0 Hz, CH_2_); ^13^C NMR (CDCl_3_,125 MHz) for **7a**: 166.8, 165.2, 148.5, 148.1, 136.2, 127.9, 127.1, 124.5, 122.7, 120.0, 119.3, 114.9, 112.9, 111.3, 110.0, 108.7, 102.2, 59.7, 51.1, 46.1, 33.4, 27.5; HRMS (ESI) for **7a**: *m/z* [M + H]^+^ calculated for C_22_H_21_BrN_3_O_4_: 470.07100, 472.06895, found: 470.07113, 470.06903; IR (KBr) for **8a**: cm^−1^ 3281, 3052, 2921, 1658, 1477, 1329, 1235, 1196, 1159, 1110, 1035;

#### 3.1.3. Crystallographic Studies

Good quality single crystals of **11a, 12a, 6a** and **1** (tadalafil) were selected for the X-ray diffraction experiments at *T* = 100(2) K. Diffraction data were collected on the Agilent Technologies SuperNova Dual Source diffractometer with Cu*K*α radiation (*λ* = 1.54184 Å) using CrysAlis RED software [CrysAlis CCD and CrysAlis RED, Oxford Diffraction, Oxford Diffraction Ltd.: Yarnton, UK, 2008]. The multi-scan empirical absorption correction using spherical harmonics (**11a**, **12a** and **1**) and analytical numeric absorption correction using a multifaceted crystal model based on expressions derived by R.C. Clark and J.S. Reid (**6a**) [54], implemented in SCALE3 ABSPACK scaling algorithm, was applied (CrysAlis CCD and CrysAlis RED, Oxford Diffraction, Oxford Diffraction Ltd.: Yarnton, 2008). The structural determination procedure was carried out using the SHELX package [55]. The structures were solved with direct methods and then successive least-square refinement was carried out based on the full-matrix least-squares method on *F*^2^ using the SHELXL program [55]. All H-atoms linked to the N-atoms were located on a Fourier difference map and refined with *U*_iso_(H) = 1.2*U*_eq_(N). In case of **11a**, the N–H bond lengths were restrained to a distance of 0.87 Å. Other H-atoms were positioned geometrically, with C–H equal to 0.93, 0.96, 0.97 and 0.98 Å for the aromatic, methyl, methylene and methine H-atoms, respectively, and constrained to ride on their parent atoms with *U*_iso_(H) = x*U*_eq_(C), where x = 1.2 for the aromatic, methylene and methine, and 1.5 for the methyl-H-atoms, respectively. All presented molecular interactions were found using the PLATON program [56]. The figures for this publication were prepared using Olex2 and Mercury programs [57,58].

### 3.2. Biology

#### 3.2.1. Cell Culture

The following human cell lines were used in this study: (i) epidermoid carcinoma A-431 (ATCC^®^ CRL-1555), (ii) lung carcinoma A549 (ATCC^®^ CCL-185), (iii) colorectal carcinoma HCT116 (ATCC^®^ CCL-247), (iv) HCT116p53^−/−^, (v) glioblastoma U-87 MG (ATCC^®^ HTB-14), (vi) glioblastoma U-251 MG (ECACC 09063001), (vii) breast adenocarcinoma MCF7 (ATCC^®^ HTB-22), (viii) breast adenocarcinoma MDA-MB-231 (ECACC 92020424), (ix) papillomavirus-related cervical squamous cell carcinoma SKG-IIIa (RRID:CVCL_1704), (x) cervical adenocarcinoma HeLa (ATCC^®^ CCL-2), (xi) bone osteosarcoma U-2 OS (ATCC^®^ HTB-96), (xii) hepatocellular carcinoma HEPG2 (ATCC^®^ HB-8065), (xiii) larynx squamous cell carcinoma BICR 18 (ECACC 06051601), (ix) kidney HEK 293T (ATCC^®^ CRL-11268), (xv) normal lung fibroblasts MRC-5 (ATCC^®^ CCL-171), (xvi) normal fibroblasts BJ (ATCC^®^ CRL-2522). The HCT116p53^−/−^ cell line was a kind gift from professor Bert Vogelstein (John Hopkin’s University, Baltimore, MD, USA). The remaining cell lines were purchased from ATCC (Manassas, VA, USA) or Sigma-Aldrich by Merck (Darmstadt, Germany).

Cells were cultured in minimal or Dulbecco’s modified Eagle’s or Ham’s F10 medium supplemented with 10% fetal bovine serum (Gibco by Thermo Fisher Scientific, Waltham, MA, USA) and (for BICR 18) 0.4 μg/mL hydrocortizone (Sigma-Aldrich by Merck, Darmstadt, Germany) under standard culture conditions (37 °C, 5% CO_2_) in a humidified incubator with an atmospheric oxygen.

#### 3.2.2. Cell Viability Assay

The viability of cells was assayed by measuring the conversion of MTT (3-(4,5-dimethylthiazol-2-yl)-2,5-diphenyltetrazolium bromide) to the formazan (the rate of this reaction is proportional to the number of surviving cells). Cells were seeded in a 96-well plate at a density of 2500–3500 cells per well, 24 h before treatment. Treatment with compounds **6a,b**, **7a,b**, tadalafil and cisplatin (2.5–160 μM) was performed for 72 h. MTT stock solution (Sigma-Aldrich) was added at the final concentration 0.5 mg/mL. After 4 h of incubation at 37 °C, water-insoluble formazan was dissolved in a lysis buffer containing 20% SDS, 50% DMF, 2.5% hydrochloric acid and 2.5% acetic acid. Optical densities were measured at 570 nm using a scanning multi-well spectrophotometer (PARADIGM Detection Platform; Beckman Coulter, Brea, CA, USA). At least three independent experiments of viability were conducted.

#### 3.2.3. Enzyme Inhibition

The inhibitory effect of the tested compounds was measured using colorimetric PDE Activity Assay Kit (Abcam, Cambridge, UK). This assay allows the quantification of 5′-nucleotide released in the reaction catalyzed by tested phosphodiesterase, PDE5, using the additional enzyme 5′-nucleotidase to cleave the mentioned product, and next, using modified Malachite Green assay, the phosphate is quantified. The assay was carried in a 96-well plate at a volume of 40 μL in an assay buffer, containing 40 ng recombinant human PDE5A/PDE5 protein (Abcam), 200 μM cGMP, 25 kU 5′-nucleotidase and serially diluted ligand to preserve 4% of DMSO. The reaction was initiated with the enzyme and kept going for 20 min at 37 °C. The reaction was stopped by adding 80 μL of Green Assay reagent. After incubation at room temperature for 30 min (to develop color proportional to phosphate content), the absorbance at 620 nm was measured with The SpectraMax iD3 Multi-Mode Microplate Reader (Molecular Devices, San Jose, CA, USA). Inhibitors concentrations varied in the range of 2 pM–200 μM. Each experiment was repeated 4–5 times and IC_50_ values were calculated by global fitting of the sigmoidal dose response equation implemented in Origin 9.0 package (www.originlab.com). For each experiment, control reaction with no enzyme added and without inhibitor was also performed.

#### 3.2.4. Platelet Aggregation in Platelet Rich Plasma

Multiplate test cells for turbidimetric aggregometry and collagen were purchased from Chronolog Co (Havertown, PA, USA). Diethylammonium (*Z*)-1-(*N*,*N*-diethylamino)diazen-1-ium-1,2-diolate (DEA-NO) was purchased from Sigma-Aldrich by Merck. The blood samples were centrifuged for 12 min at 190× *g* to obtain platelet-rich plasma (PRP). The platelet count in the PRP was adjusted to 2 × 10^8^ platelets/mL prior to its use in experiments. Five minutes prior to measurements, samples were added with a tested compound (dissolved in 0.1% DMSO), control samples were added with 0.1% DMSO. DEA-NO was added to the final concentration of 500 nM, and shortly after, collagen was added to each sample to a final concentration of 2 μg/mL to induce platelet aggregation, which was thereafter monitored for 10 min using an optical aggregometer Chrono-Log 490-2D (Chrono-Log, Havertown, PA, USA). The total area under the aggregation curve (AUC) and the maximal value of platelet aggregation (Amax,U) was recorded.

#### 3.2.5. Patients and Blood Collection

Blood was drawn from healthy donors. The samples were collected into a vacuum tube containing 0.105 M buffered sodium citrate, for optical aggregation. The study was performed under the guidelines of the Helsinki Declaration for human research and approved by the Medical University of Lodz committee on the Ethics of Research in Human Experimentation (approval numer: RNN/43/17/KE).

#### 3.2.6. Caenorhabditis Elegans

Worms were maintained and handled in standard conditions, as described by Brenner [59]. Worms were cultured at 20 °C on plates seeded with *E. coli* HB101. Wild type *C. elegans* N2 strain was used in this study. For the toxicological test, worms were cultured in liquid S-medium in 96 well plates. Each well contained 100 μL medium and was seeded with heat inactivated HB101 bacteria. 50 L4 worms were transferred to each well. The viability of worms was assessed after 24 and 48 h by visual inspection; thrashing worms were scored alive and straight, non-moving as dead.

## 4. Conclusions

In the present study, we synthesized four novel analogues of PDE5 inhibitor—tadalafil, and compared their physicochemical and biological properties with the parent compound. The obtained crystal structures, due to ligands flexibility, revealed significant structural differences and interaction schemes in the solid state. In the cytotoxic assays, in the most cases, newly synthesized compounds exhibited a weaker effect than tadalafil, except for bromine-substituted compounds **7a** and **7b**, which showed a stronger cytotoxic effect then the parent compound on HEK 293T and MC F7 cell lines. The effect on MCF7 is especially interesting, as IC_50_ = 55.29 μM observed for tadalafil dropped to the 28.9 μM in case of (*R*)-3-((1*H*-indol-3-yl)methyl)-4-((6-bromobenzo[*d*][1,3]dioxol-5-yl)methyl)-1-methylpiperazine-2,5-dione (**7a**) and 36.32 μM for (*S*)-3-((1*H*-indol-3-yl)methyl)-4-((6-bromobenzo[*d*][1,3]dioxol-5-yl)methyl)-1-methylpiperazine-2,5-dione (**7b**). As the newly prepared compounds, in two different independent assays, turned out to be significantly weaker PDE5 inhibitors, we concluded that structural changes introduced to the tadalafil structure diminished their interactions with enzyme, and the rigidity of molecules is a key factor responsible for its biological activity. Consequently, the enhanced cytotoxic effect observed in the breast cancer cell line MCF7, worthy of further biological research, is not in this case related to the inhibition of PDE5 activity, but results from another, yet undiscovered mechanism of action. The synthesized products are low molecular weight compounds with lead-like properties suitable for a further medicinal chemistry optimization program. Further research on the improvement of the cytotoxic effect and the selectivity of the tested compounds, especially to the MCF7 breast cancer cell line, is being conducted in our laboratory and will be published in due course.

## Figures and Tables

**Table 1:** Calculated IC_50_ values of tadalafil and its analogues **6a,b**, **7a,b**, and cisplatin on selected human cell lines.

Comp. No.	6a	6b	7a	7b	Tadalafil	Cisplatin
Cell Line	IC_50_ Values (μM)					
**A-431**	**104.1** (29.58–366.3) *	**111.8** (52.68–237.3) *	**63.56** (37.34–108.2) *	**46.56** (28.02–77.39) *	**20.36** (15.13–27.39) *	**1.51** (1.02–2.22) *
**A549**	[62.67] **	[62.85] **	**127.6** (116–140.3) *	**130.6** (119.7–142.5) *	**51.51** (41.69–63.65) *	**27.22** (20.9–35.46) *
**HCT116**	**102** (73.02–142.4) *	**83.74** (67.19–104.4) *	**52.99** (39.18–71.68) *	**58.73** (39.9–86.44) *	**27.25** (22.29–33.31) *	**7.44** (6.44–8.6) *
**HCT116p53^−/−^**	**83.21** (21.27–325.5) *	**143** (48.92–417.9) *	**35.93** (20.05–64.39) *	**64.42** (36.06–115.1) *	**19.77** (15.89–24.59) *	**14.12** (10.44–19.09) *
**U-87 MG**	**153.2** (97.54–240.6) *	**151.2** (116.6–196) *	**109.7** (101.4–118.7) *	**98.99** (91.26–107.4) *	**40.66** (33.27–49.7) *	**4.96** (3.39–7.25) *
**U-251 MG**	[55.76] **	[47.58] **	[81.34] **	[52.48] **	**44.13** (37.43–52.04) *	**2.61** (1.57–4.34) *
**MCF7**	**53.20** (41.97–67.44) *	**54.05** (44.27–65.99) *	**28.9** (23.15–36.07) *	**36.32** (30.85–42.77) *	**55.29** (46.56–65.64) *	**10.99** (9.22–13.11) *
**MDA-MB-231**	[84.18] **	**128** (118.7–138) *	[75.86] **	**143.6** (132.6–155.5) *	**87.6** (76.56–100.2) *	**22.3** (16.37–30.38) *
**SKGIIIa**	**145.5** (108.9–194.6) *	**87.58** (77.73–98.68) *	**75.94** (63.71–90.52) *	**57.28** (46.02–71.29) *	**35.08** (29.88–41.19) *	**4.84** (*2.96–7.93*) *
**HeLa**	**119.9** (96.9–148.5) *	**93.49** (74.63–117.1) *	**79.19** (57.88–108.4) *	**78.39** (65.68–93.57) *	**42.29** (33.45–53.46) *	**13.52** (11.46–15.96) *
**U-2 OS**	**93.52** (28.41–307.8) *	**88.26** (70.31–110.8) *	**95.39** (83.27–109.3) *	**88.83** (74.16–106.4) *	**35.06** (31.77–38.7) *	**7.55** (6.36–8.96) *
**HEPG2**	[50.9] **	[76.76] **	**103.3** (83.65–127.5) *	**123.9** (109.7–139.9) *	**46.56** (35.22–61.55) *	**7.8** (6.77–8.99) *
**BICR 18**	[72.57] **	[56.72] **	**137.1** (119.8–156.9) *	**141.9** (108.6–185.5) *	**52.53** (34.89–79.08) *	**1.69** (1.15–2.48) *
**HEK 293T**	**10.52** (6.64–16.68) *	**12.95** (9.9–16.93) *	**12.34** (9.59–15.87) *	**13.06** (10.98–15.55) *	**30.96** (19.52–49.12) *	**6.25** (5.17–7.55) *
**MRC-5**	[56.68] **	**127** (103.7–155.6) *	**116.3** (109.1–124) *	**96.57** (81.85–113.9) *	**28.72** (23.33–35.37) *	**3.92** (3.23–4.76) *
**BJ**	[60.84] **	**142.8** (105.1–194.1) *	**117** (103.9–131.7) *	**102.6** (95.38–110.4) *	**40.48** (33.11–49.50) *	**5.58** (4.64–6.71) *

IC_50_—half maximal inhibitory concentration (lethal dose for 50% of cells). IC_50_ values were calculated from the nonlinear fit of the dose-log response curves done in GraphPad Prism 5.03. Values are medians of at least three independent experiments. * 95% Confidence Intervals, ** % of proliferation inhibition at highest concentration used—160 μM.

**Table 2:** Calculated IC_50_ and Ki values of tadalafil and its analogues **6a,b**, **7a,b** against human PDE5.

Compound No.	IC_50_ (μM)	Ki (μM)
**6a**	137 ± 47	1.4 ± 0.8
**6b**	260 ± 210	2.6 ± 2.7
**7a**	22 ± 12	0.22 ± 0.17
**7b**	39 ± 27	0.39 ± 0.36
tadalafil	0.072 ± 0.031	(7.1 ± 4.8) × 10^−4^

IC_50_—half maximal inhibitory concentration. IC_50_ values were calculated by global fitting of the sigmoidal dose-response equation done in Origin 9.0.

## Data Availability

The CCDC 2049073–2049076 contain the supplementary crystallographic data for this paper. The data can be obtained free of charge from the Cambridge Crystallographic Data Centre via www.ccdc.cam.ac.uk/structures.

## Supplementary Materials

The following are available online, Table S1: Crystal data and structure refinement for investigated compounds, Table S2: Bond lengths for **11a**, Table S3: Valence angles for **11a**, Table S4: Torsion angles for **11a**, Table S5: Bond lengths for **12a**, Table S6: Valence angles for **12a**, Table S7: Torsion angles for **12a**, Table S8: Bond lengths for **6a**, Table S9: Valence angles for **6a**, Table S10: Torsion angles for **6a**, Table S11: Bond lengths for **1**, Table S12: Valence angles for **1**, Table S13: Torsion angles for **1**, Table S14: The geometry of hydrogen bonds in the crystal of **11a**, Table S15: The geometry of C–H⋯π contacts in the crystal of **11a**, Table S16: The geometry of C=O⋯π contacts in the crystal of **11a**, Table S17: The geometry of hydrogen bonds in the crystal of **12a**, Table S18: The geometry of C–H⋯π contacts in the crystal of **12a**, **Table S19**: The geometry of hydrogen bonds in the crystal of **6a**, Table S20: The geometry of C–H⋯π contacts in the crystal of **6a**, Table S21: The geometry of hydrogen bonds in the crystal of **1**, Table S22: The geometry of C–H⋯π contacts in the crystal of **1**, Figure S1: 1H-NMR spectrum for **11a**, Figure S2: 1H-NMR spectrum for **11b**, Figure S3: 1H-NMR spectrum for **12a**, Figure S4: 1H-NMR spectrum for **12b**, Figure S5: 1H-NMR spectrum for **13a**, Figure S6: 1H-NMR spectrum for **13b**, Figure S7: 1H-NMR spectrum for **6a**, Figure S8: 1H-NMR spectrum for **6b**, Figure S9: 1H-NMR spectrum for **7a**, Figure S10: 1H-NMR spectrum for **7b**, Figure S11: 13-NMR spectrum for **11a**, Figure S12: 13C-NMR spectrum for **11b**, Figure S13: 13C-NMR spectrum for **12a**, Figure S14: 13C-NMR spectrum for **12b**, Figure S15: 13C-NMR spectrum for **13a**, Figure S16: 13C-NMR spectrum for **13b,** Figure S17: 13C-NMR spectrum for **6a**, Figure S18: 13C-NMR spectrum for **6b**, Figure S19: 13C-NMR spectrum for **7a**, Figure S20: 13C-NMR spectrum for **7b**, Figure S21: **IR** spectrum for **11a**, Figure S22: **IR** spectrum for **11b**, Figure S23: **IR** spectrum for **12a**, Figure S24: **IR** spectrum for **12b**, Figure S25: **IR** spectrum for **13a**, Figure S26: **IR** spectrum for **13b**, Figure S27: **IR** spectrum for **6a**, Figure S28: **IR** spectrum for **6b**, Figure S29: IR spectrum for **7a**, Figure S30: IR spectrum for **7b**, Figure S31: HRMS spectrum for **11a**, Figure S32: HRMS spectrum for **11b**, Figure S33: HRMS spectrum for **12a**, Figure S34: HRMS spectrum for **12b**, Figure S35: HRMS spectrum for **13a**, Figure S36: HRMS spectrum for **13b**, Figure S37: HRMS spectrum for **6a**, Figure S38: HRMS spectrum for **6b**, Figure S39: HRMS spectrum for **7a**, Figure S40: HRMS spectrum for **7b**, Figure S41: Absorbance at 620 nm used for IC_50_ value determinations of (a) **6a**, (b) **6b**, (c) **7a**, (d) **7b** and (e) tadalafil against PDE5. Both control data points are marked on graphs.

## Sample Availability

Samples of the compounds **6a,b**, **7a,b** are available from the authors.
