# Supplementary material for: Diketopiperazine-Based, Flexible Tadalafil Analogues: Synthesis, Crystal Structures and Biological Activity Profile
Source: Molecules. 2021 Feb 3;26(4):794. doi: 10.3390/molecules26040794 (PMC7913621; doi:10.3390/molecules26040794)
Supplement: Supplementary file 1 [file molecules-26-00794-s001.pdf]

Article

Diketopiperazine-based, Flexible Tadalafil Analogues: Synthesis, Crystal Structures and Biological Activity Profile

Adam Mieczkowski [1,*,†], Elżbieta Speina [1,†], Damian Trzybiński [2], Maria Winiewska-Szajewska [1], Patrycja Wińska [3], Ewelina M. Borsuk [1], Małgorzata Podsiadła-Białoskórska [1], Tomasz Przygodzki [4], Krzysztof Drabikowski [1], Lidia Stanczyk [4], Igor Zhukov [1], Cezary Watala [4] and Krzysztof Woźniak [2]

[1] Institute of Biochemistry and Biophysics, Polish Academy of Sciences, Pawinskiego 5a, 02-106 Warsaw, Poland; elasp@ibb.waw.pl (E.S.); mwin@ibb.waw.pl (M.W.-S.); e.szmajda@ibb.waw.pl (E.M.B.); weronika@ibb.waw.pl (M.P.-B.) drabikowski@ibb.waw.pl (K.D.) igor@ibb.waw.pl (I.Z.)
[2] Biological and Chemical Research Centre, University of Warsaw, Żwirki i Wigury 101, 02-089 Warsaw, Poland; dtrzybinski@cnbc.uw.edu.pl (D.T.); kwozniak@chem.uw.edu.pl (K.W.)
[3] Faculty of Chemistry, Warsaw University of Technology, Noakowskiego 3, 00-664 Warsaw, Poland; pwinska@ch.pw.edu.pl
[4] Department of Haemostatic Disorders, Chair of Biomedical Sciences, Faculty of Health Sciences, Medical University of Lodz, 6/8 Mazowiecka Street, 92-235 Lodz, Poland; tomasz.przygodzki@umed.lodz.pl (T.P.); lidiastanczyk57@gmail.com (L.S.); cezary.watala@umed.lodz.pl (C.W.)
† These authors contributed equally to this work.
* Correspondence: amiecz@ibb.waw.pl; Tel.: +48-22-592-3506

SUPPORTING INFORMATION -

crystallographic data, 1H-NMR, 13C-NMR, ESI HRMS, IR spectra

Table S1. Crystal data and structure refinement for investigated compounds.

**Identification code**	**11a**	**12a**	**6a**	**1 (tadalafil)**
Empirical formula	$C_{38}H_{46}N_6O_8$	$C_{27}H_{29}N_3O_6$	$C_{22}H_{21}N_3O_4$	$C_{22}H_{19}N_3O_4$
Formula weight	714.81	491.53	391.42	389.40
Temperature/K	100(2)	100(2)	100(2)	100(2)
Crystal system	Monoclinic	Orthorhombic	Orthorhombic	monoclinic
Space group	$P2_1$	$P2_12_12_1$	$P2_12_12_1$	$P2_1$

a/Å	6.3558(4)	6.2900(4)	9.9729(5)	9.8319(4)
b/Å	29.7177(15)	14.9183(11)	11.0468(6)	7.6926(4)
c/Å	9.9780(7)	25.996(3)	17.1587(10)	12.2711(5)
α/°	90	90	90	90
β/°	96.816(6)	90	90	99.436(4)
γ/°	90	90	90	90
Volume/Å^3	1871.32(19)	2439.3(3)	1890.35(18)	915.54(7)
Z	2	4	4	2
ρ_{calc}g/cm^3	1.269	1.338	1.375	1.413
μ/mm^{-1}	0.739	0.785	0.789	0.814
F(000)	760.0	1040.0	824.0	408.0
Crystal size/mm^3	0.30 × 0.09 × 0.06	0.41 × 0.07 × 0.04	0.33 × 0.23 × 0.07	0.47 × 0.06 × 0.05
Radiation	Cu$K\alpha$ (λ = 1.54184)	Cu$K\alpha$ (λ = 1.54184)	Cu$K\alpha$ (λ = 1.54184)	Cu$K\alpha$ (λ = 1.54184)
2Θ range for data collection/°	5.948 to 134.138	6.8 to 134.112	9.522 to 134.008	7.302 to 134.144
Index ranges	$-7 \le h \le 7$, $-34 \le k \le 35$, $-11 \le l \le 11$	$-7 \le h \le 6$, $-16 \le k \le 17$, $-26 \le l \le 31$	$-9 \le h \le 11$, $-13 \le k \le 13$, $-20 \le l \le 19$	$-11 \le h \le 11$, $-9 \le k \le 8$, $-14 \le l \le 14$
Reflections collected	15055	8162	6228	6799
Independent reflections	6604 [R_{int} = 0.0539, R_{sigma} = 0.0727]	4357 [R_{int} = 0.0743, R_{sigma} = 0.1204]	3361 [R_{int} = 0.0286, R_{sigma} = 0.0454]	3151 [R_{int} = 0.0388, R_{sigma} = 0.0484]
Data/restraints/parameters	6604/3/483	4357/0/329	3361/0/266	3151/1/266
Goodness-of-fit on F^2	1.037	0.986	1.052	1.047
Final R indexes [I>=2σ (I)]	R_1 = 0.0581, wR_2 = 0.1392	R_1 = 0.0612, wR_2 = 0.1163	R_1 = 0.0399, wR_2 = 0.0937	R_1 = 0.0385, wR_2 = 0.0912
Final R indexes [all data]	R_1 = 0.0716, wR_2 = 0.1521	R_1 = 0.1043, wR_2 = 0.1435	R_1 = 0.0502, wR_2 = 0.1012	R_1 = 0.0438, wR_2 = 0.0959
Largest diff. peak/hole / e Å^{-3}	0.76/-0.32	0.26/-0.28	0.16/-0.19	0.15/-0.19
Flack parameter	0.07(17)	0.0(4)	0.00(15)	0.10(17)

Table S2. Bond lengths for **11a**.

Atom	Atom	Length/Å	Atom	Atom	Length/Å
O(3A)	C(16A)	1.228(6)	C(16B)	C(15B)	1.525(7)
O(4A)	C(18A)	1.227(7)	C(1A)	C(2A)	1.363(7)
O(1A)	C(9A)	1.319(7)	C(15A)	C(16A)	1.531(8)
O(1A)	C(10A)	1.497(6)	C(15A)	C(14A)	1.552(7)
O(4B)	C(18B)	1.236(7)	C(8B)	C(3B)	1.405(7)
O(1B)	C(9B)	1.332(7)	C(8B)	C(7B)	1.389(8)
O(1B)	C(10B)	1.489(6)	C(3B)	C(4B)	1.409(8)
O(2B)	C(9B)	1.202(7)	C(3B)	C(2B)	1.441(8)
O(3B)	C(16B)	1.226(7)	C(7B)	C(6B)	1.386(8)
O(2A)	C(9A)	1.199(7)	C(2A)	C(3A)	1.447(7)
N(2B)	C(18B)	1.331(7)	C(2A)	C(14A)	1.498(7)
N(2B)	C(15B)	1.461(6)	C(4B)	C(5B)	1.381(8)
N(3A)	C(16A)	1.338(7)	C(3A)	C(8A)	1.413(7)
N(3A)	C(17A)	1.452(7)	C(3A)	C(4A)	1.414(8)
N(3A)	C(19A)	1.448(7)	C(8A)	C(7A)	1.386(7)
N(3B)	C(16B)	1.333(7)	C(14B)	C(15B)	1.555(7)
N(3B)	C(17B)	1.457(7)	C(14B)	C(2B)	1.516(7)
N(3B)	C(19B)	1.460(7)	C(10B)	C(13B)	1.515(8)
N(1B)	C(8B)	1.409(8)	C(10B)	C(12B)	1.526(8)
N(1B)	C(9B)	1.390(7)	C(10B)	C(11B)	1.520(8)
N(1B)	C(1B)	1.399(7)	C(1B)	C(2B)	1.350(8)
N(1A)	C(1A)	1.397(7)	C(6A)	C(7A)	1.397(8)
N(1A)	C(9A)	1.416(7)	C(6A)	C(5A)	1.405(8)
N(1A)	C(8A)	1.411(7)	C(4A)	C(5A)	1.377(8)
N(2A)	C(18A)	1.327(7)	C(6B)	C(5B)	1.396(8)
N(2A)	C(15A)	1.451(6)	C(12A)	C(10A)	1.520(8)
C(18A)	C(17A)	1.515(8)	C(13A)	C(10A)	1.527(8)
C(18B)	C(17B)	1.506(7)	C(10A)	C(11A)	1.510(8)

Table S3. Valence angles for **11a**.

Atom	Atom	Atom	Angle/°	Atom	Atom	Atom	Angle/°
C(9A)	O(1A)	C(10A)	119.0(4)	C(5B)	C(4B)	C(3B)	118.8(5)
C(9B)	O(1B)	C(10B)	120.2(4)	C(8A)	C(3A)	C(2A)	108.5(5)
C(18B)	N(2B)	C(15B)	124.2(4)	C(8A)	C(3A)	C(4A)	118.5(5)
C(16A)	N(3A)	C(17A)	122.4(5)	C(4A)	C(3A)	C(2A)	133.0(5)
C(16A)	N(3A)	C(19A)	119.3(4)	O(1A)	C(9A)	N(1A)	108.9(4)
C(19A)	N(3A)	C(17A)	116.3(4)	O(2A)	C(9A)	O(1A)	129.3(5)
C(16B)	N(3B)	C(17B)	124.3(5)	O(2A)	C(9A)	N(1A)	121.8(5)
C(16B)	N(3B)	C(19B)	119.0(5)	N(1A)	C(8A)	C(3A)	106.3(4)
C(17B)	N(3B)	C(19B)	115.5(4)	C(7A)	C(8A)	N(1A)	130.7(5)
C(9B)	N(1B)	C(8B)	125.1(5)	C(7A)	C(8A)	C(3A)	123.1(5)
C(9B)	N(1B)	C(1B)	127.2(5)	O(1B)	C(9B)	N(1B)	109.7(5)
C(1B)	N(1B)	C(8B)	107.6(4)	O(2B)	C(9B)	O(1B)	127.1(5)
C(1A)	N(1A)	C(9A)	126.9(4)	O(2B)	C(9B)	N(1B)	123.2(5)
C(1A)	N(1A)	C(8A)	108.4(4)	N(3A)	C(17A)	C(18A)	115.5(4)
C(8A)	N(1A)	C(9A)	124.4(4)	C(2B)	C(14B)	C(15B)	112.2(4)
C(18A)	N(2A)	C(15A)	124.8(5)	O(1B)	C(10B)	C(13B)	110.5(4)
O(4A)	C(18A)	N(2A)	124.2(5)	O(1B)	C(10B)	C(12B)	108.9(4)
O(4A)	C(18A)	C(17A)	119.6(5)	O(1B)	C(10B)	C(11B)	102.4(4)
N(2A)	C(18A)	C(17A)	116.3(5)	C(13B)	C(10B)	C(12B)	112.8(5)
O(4B)	C(18B)	N(2B)	123.9(5)	C(13B)	C(10B)	C(11B)	110.8(5)
O(4B)	C(18B)	C(17B)	118.7(5)	C(11B)	C(10B)	C(12B)	111.1(5)
N(2B)	C(18B)	C(17B)	117.3(5)	C(2B)	C(1B)	N(1B)	110.7(5)
O(3B)	C(16B)	N(3B)	124.0(5)	C(7A)	C(6A)	C(5A)	120.9(5)
O(3B)	C(16B)	C(15B)	118.1(5)	C(8A)	C(7A)	C(6A)	117.1(5)
N(3B)	C(16B)	C(15B)	117.9(5)	N(2B)	C(15B)	C(16B)	113.9(4)
C(2A)	C(1A)	N(1A)	110.5(5)	N(2B)	C(15B)	C(14B)	110.8(4)
N(2A)	C(15A)	C(16A)	112.8(4)	C(16B)	C(15B)	C(14B)	108.9(4)
N(2A)	C(15A)	C(14A)	112.4(4)	C(5A)	C(4A)	C(3A)	118.8(5)
C(16A)	C(15A)	C(14A)	108.4(4)	C(2A)	C(14A)	C(15A)	112.1(4)
C(3B)	C(8B)	N(1B)	106.9(5)	N(3B)	C(17B)	C(18B)	116.1(4)
C(7B)	C(8B)	N(1B)	130.4(5)	C(4A)	C(5A)	C(6A)	121.6(5)
C(7B)	C(8B)	C(3B)	122.8(5)	C(3B)	C(2B)	C(14B)	127.0(5)
C(8B)	C(3B)	C(4B)	118.7(5)	C(1B)	C(2B)	C(3B)	106.8(5)
C(8B)	C(3B)	C(2B)	108.1(5)	C(1B)	C(2B)	C(14B)	126.2(5)
C(4B)	C(3B)	C(2B)	133.2(5)	C(7B)	C(6B)	C(5B)	121.6(5)
C(6B)	C(7B)	C(8B)	117.1(5)	C(4B)	C(5B)	C(6B)	121.1(5)
O(3A)	C(16A)	N(3A)	123.3(5)	O(1A)	C(10A)	C(12A)	109.7(5)
O(3A)	C(16A)	C(15A)	118.8(5)	O(1A)	C(10A)	C(13A)	108.8(4)
N(3A)	C(16A)	C(15A)	117.9(4)	O(1A)	C(10A)	C(11A)	102.5(4)
C(1A)	C(2A)	C(3A)	106.3(5)	C(12A)	C(10A)	C(13A)	113.1(5)
C(1A)	C(2A)	C(14A)	126.2(5)	C(11A)	C(10A)	C(12A)	111.2(5)
C(3A)	C(2A)	C(14A)	127.3(5)	C(11A)	C(10A)	C(13A)	111.0(5)

Table S4. Torsion angles for **11a**.

A	B	C	D	Angle/°	A	B	C	D	Angle/°
O(4A)	C(18A)	C(17A)	N(3A)	-160.0(5)	C(3A)	C(2A)	C(14A)	C(15A)	85.2(6)
O(4B)	C(18B)	C(17B)	N(3B)	-172.5(4)	C(3A)	C(8A)	C(7A)	C(6A)	-2.0(8)

O(3B)	C(16B)	C(15B)	N(2B)	-166.7(5)	C(3A)	C(4A)	C(5A)	C(6A)	0.0(9)
O(3B)	C(16B)	C(15B)	C(14B)	69.0(6)	C(9A)	O(1A)	C(10A)	C(12A)	64.4(6)
N(2B)	C(18B)	C(17B)	N(3B)	4.3(7)	C(9A)	O(1A)	C(10A)	C(13A)	-59.9(6)
N(3B)	C(16B)	C(15B)	N(2B)	13.9(6)	C(9A)	O(1A)	C(10A)	C(11A)	-177.4(5)
N(3B)	C(16B)	C(15B)	C(14B)	-110.4(5)	C(9A)	N(1A)	C(1A)	C(2A)	-173.0(5)
N(1B)	C(8B)	C(3B)	C(4B)	-179.5(4)	C(9A)	N(1A)	C(8A)	C(3A)	173.9(5)
N(1B)	C(8B)	C(3B)	C(2B)	1.8(5)	C(9A)	N(1A)	C(8A)	C(7A)	-6.3(9)
N(1B)	C(8B)	C(7B)	C(6B)	179.0(5)	C(8A)	N(1A)	C(1A)	C(2A)	1.3(6)
N(1B)	C(1B)	C(2B)	C(3B)	1.2(6)	C(8A)	N(1A)	C(9A)	O(1A)	-175.0(4)
N(1B)	C(1B)	C(2B)	C(14B)	178.0(4)	C(8A)	N(1A)	C(9A)	O(2A)	4.6(8)
N(1A)	C(1A)	C(2A)	C(3A)	-1.4(6)	C(8A)	C(3A)	C(4A)	C(5A)	-0.2(8)
N(1A)	C(1A)	C(2A)	C(14A)	173.8(5)	C(9B)	O(1B)	C(10B)	C(13B)	-60.2(6)
N(1A)	C(8A)	C(7A)	C(6A)	178.2(5)	C(9B)	O(1B)	C(10B)	C(12B)	64.2(6)
N(2A)	C(18A)	C(17A)	N(3A)	19.9(7)	C(9B)	O(1B)	C(10B)	C(11B)	-178.1(5)
N(2A)	C(15A)	C(16A)	O(3A)	-159.8(4)	C(9B)	N(1B)	C(8B)	C(3B)	174.5(5)
N(2A)	C(15A)	C(16A)	N(3A)	21.4(6)	C(9B)	N(1B)	C(8B)	C(7B)	-5.9(8)
N(2A)	C(15A)	C(14A)	C(2A)	-61.4(6)	C(9B)	N(1B)	C(1B)	C(2B)	-175.5(5)
C(18A)	N(2A)	C(15A)	C(16A)	-29.8(6)	C(17A)	N(3A)	C(16A)	O(3A)	-172.7(5)
C(18A)	N(2A)	C(15A)	C(14A)	93.2(6)	C(17A)	N(3A)	C(16A)	C(15A)	6.1(7)
C(18B)	N(2B)	C(15B)	C(16B)	-28.2(7)	C(10B)	O(1B)	C(9B)	O(2B)	3.5(8)
C(18B)	N(2B)	C(15B)	C(14B)	95.1(6)	C(10B)	O(1B)	C(9B)	N(1B)	-178.2(4)
C(16B)	N(3B)	C(17B)	C(18B)	-17.6(7)	C(1B)	N(1B)	C(8B)	C(3B)	-1.1(5)
C(1A)	N(1A)	C(9A)	O(1A)	-1.5(7)	C(1B)	N(1B)	C(8B)	C(7B)	178.6(5)
C(1A)	N(1A)	C(9A)	O(2A)	178.1(5)	C(1B)	N(1B)	C(9B)	O(1B)	3.1(7)
C(1A)	N(1A)	C(8A)	C(3A)	-0.6(5)	C(1B)	N(1B)	C(9B)	O(2B)	-178.6(5)
C(1A)	N(1A)	C(8A)	C(7A)	179.2(5)	C(7A)	C(6A)	C(5A)	C(4A)	-0.9(9)
C(1A)	C(2A)	C(3A)	C(8A)	1.0(6)	C(15B)	N(2B)	C(18B)	O(4B)	-164.8(5)
C(1A)	C(2A)	C(3A)	C(4A)	179.4(6)	C(15B)	N(2B)	C(18B)	C(17B)	18.7(7)
C(1A)	C(2A)	C(14A)	C(15A)	-89.1(6)	C(15B)	C(14B)	C(2B)	C(3B)	100.0(6)
C(15A)	N(2A)	C(18A)	O(4A)	-171.1(5)	C(15B)	C(14B)	C(2B)	C(1B)	-76.3(6)
C(15A)	N(2A)	C(18A)	C(17A)	9.0(7)	C(4A)	C(3A)	C(8A)	N(1A)	-178.9(5)
C(8B)	N(1B)	C(9B)	O(1B)	-171.6(4)	C(4A)	C(3A)	C(8A)	C(7A)	1.2(8)
C(8B)	N(1B)	C(9B)	O(2B)	6.7(8)	C(14A)	C(15A)	C(16A)	O(3A)	75.1(5)
C(8B)	N(1B)	C(1B)	C(2B)	-0.1(6)	C(14A)	C(15A)	C(16A)	N(3A)	-103.8(5)
C(8B)	C(3B)	C(4B)	C(5B)	1.1(8)	C(14A)	C(2A)	C(3A)	C(8A)	-174.2(5)
C(8B)	C(3B)	C(2B)	C(14B)	-178.7(4)	C(14A)	C(2A)	C(3A)	C(4A)	4.2(10)
C(8B)	C(3B)	C(2B)	C(1B)	-1.8(6)	C(17B)	N(3B)	C(16B)	O(3B)	-171.7(5)
C(8B)	C(7B)	C(6B)	C(5B)	0.0(8)	C(17B)	N(3B)	C(16B)	C(15B)	7.6(7)
C(3B)	C(8B)	C(7B)	C(6B)	-1.4(7)	C(5A)	C(6A)	C(7A)	C(8A)	1.8(8)
C(3B)	C(4B)	C(5B)	C(6B)	-2.5(8)	C(2B)	C(3B)	C(4B)	C(5B)	179.5(5)
C(7B)	C(8B)	C(3B)	C(4B)	0.8(7)	C(2B)	C(14B)	C(15B)	N(2B)	-57.0(5)
C(7B)	C(8B)	C(3B)	C(2B)	-177.9(4)	C(2B)	C(14B)	C(15B)	C(16B)	69.1(5)
C(7B)	C(6B)	C(5B)	C(4B)	2.0(9)	C(19B)	N(3B)	C(16B)	O(3B)	-5.1(8)
C(16A)	N(3A)	C(17A)	C(18A)	-27.6(7)	C(19B)	N(3B)	C(16B)	C(15B)	174.3(5)
C(16A)	C(15A)	C(14A)	C(2A)	64.1(5)	C(19B)	N(3B)	C(17B)	C(18B)	175.4(5)
C(2A)	C(3A)	C(8A)	N(1A)	-0.3(6)	C(19A)	N(3A)	C(16A)	O(3A)	-9.4(8)
C(2A)	C(3A)	C(8A)	C(7A)	179.9(5)	C(19A)	N(3A)	C(16A)	C(15A)	169.4(5)
C(2A)	C(3A)	C(4A)	C(5A)	-178.4(6)	C(19A)	N(3A)	C(17A)	C(18A)	168.7(5)
C(4B)	C(3B)	C(2B)	C(14B)	2.8(9)	C(10A)	O(1A)	C(9A)	O(2A)	-3.8(8)

C(4B)	C(3B)	C(2B)	C(1B)	179.7(6)	C(10A)	O(1A)	C(9A)	N(1A)	175.7(4)

Table S5. Bond lengths for **12a**.

Atom	Atom	Length/Å	Atom	Atom	Length/Å
C(1)	C(2)	1.356(8)	C(15)	N(2)	1.457(7)
C(1)	N(1)	1.403(7)	C(16)	C(17)	1.511(8)
C(2)	C(3)	1.444(8)	C(16)	N(2)	1.342(7)
C(2)	C(14)	1.491(8)	C(16)	O(3)	1.240(7)
C(3)	C(4)	1.393(9)	C(17)	N(3)	1.455(7)
C(3)	C(8)	1.418(9)	C(18)	N(3)	1.331(7)
C(4)	C(5)	1.384(9)	C(18)	O(4)	1.240(7)
C(5)	C(6)	1.396(10)	C(19)	C(20)	1.515(8)
C(6)	C(7)	1.387(10)	C(19)	N(2)	1.488(8)
C(7)	C(8)	1.387(9)	C(20)	C(21)	1.395(8)
C(8)	N(1)	1.412(8)	C(20)	C(25)	1.400(8)
C(9)	N(1)	1.396(8)	C(21)	C(22)	1.392(9)
C(9)	O(1)	1.329(8)	C(22)	C(23)	1.350(9)
C(9)	O(2)	1.218(7)	C(23)	C(24)	1.390(8)
C(10)	C(11)	1.523(9)	C(23)	O(6)	1.375(8)
C(10)	C(12)	1.512(9)	C(24)	C(25)	1.348(9)
C(10)	C(13)	1.513(9)	C(24)	O(5)	1.382(7)
C(10)	O(1)	1.489(7)	C(26)	O(5)	1.404(8)
C(14)	C(15)	1.558(8)	C(26)	O(6)	1.421(8)
C(15)	C(18)	1.511(8)	C(27)	N(3)	1.466(8)

Table S6. Valence angles for **12a**.

Atom	Atom	Atom	Angle/°	Atom	Atom	Atom	Angle/°
C(2)	C(1)	N(1)	109.8(6)	N(3)	C(17)	C(16)	114.4(5)
C(1)	C(2)	C(3)	107.6(6)	N(3)	C(18)	C(15)	117.2(5)
C(1)	C(2)	C(14)	125.0(6)	O(4)	C(18)	C(15)	119.2(5)
C(3)	C(2)	C(14)	127.3(6)	O(4)	C(18)	N(3)	123.5(6)
C(4)	C(3)	C(2)	133.8(6)	N(2)	C(19)	C(20)	112.0(5)
C(4)	C(3)	C(8)	118.5(6)	C(21)	C(20)	C(19)	119.9(6)
C(8)	C(3)	C(2)	107.7(6)	C(21)	C(20)	C(25)	119.8(6)
C(5)	C(4)	C(3)	119.4(7)	C(25)	C(20)	C(19)	120.2(6)
C(4)	C(5)	C(6)	120.7(7)	C(22)	C(21)	C(20)	121.3(6)
C(7)	C(6)	C(5)	121.7(7)	C(23)	C(22)	C(21)	117.6(6)
C(6)	C(7)	C(8)	117.0(7)	C(22)	C(23)	C(24)	121.3(6)
C(7)	C(8)	C(3)	122.7(6)	C(22)	C(23)	O(6)	129.3(6)
C(7)	C(8)	N(1)	130.9(6)	O(6)	C(23)	C(24)	109.4(6)
N(1)	C(8)	C(3)	106.4(5)	C(25)	C(24)	C(23)	122.5(6)
O(1)	C(9)	N(1)	109.5(5)	C(25)	C(24)	O(5)	128.4(6)
O(2)	C(9)	N(1)	123.1(6)	O(5)	C(24)	C(23)	109.0(6)
O(2)	C(9)	O(1)	127.3(6)	C(24)	C(25)	C(20)	117.5(6)
C(12)	C(10)	C(11)	112.8(6)	O(5)	C(26)	O(6)	108.9(6)
C(12)	C(10)	C(13)	111.3(6)	C(1)	N(1)	C(8)	108.5(5)
C(13)	C(10)	C(11)	111.4(6)	C(9)	N(1)	C(1)	125.3(5)
O(1)	C(10)	C(11)	109.9(6)	C(9)	N(1)	C(8)	125.9(5)
O(1)	C(10)	C(12)	109.7(6)	C(15)	N(2)	C(19)	117.1(5)

O(1)	C(10)	C(13)	101.0(5)	C(16)	N(2)	C(15)	122.9(5)
C(2)	C(14)	C(15)	113.1(5)	C(16)	N(2)	C(19)	119.3(5)
C(18)	C(15)	C(14)	110.2(5)	C(17)	N(3)	C(27)	115.5(5)
N(2)	C(15)	C(14)	111.6(5)	C(18)	N(3)	C(17)	123.7(5)
N(2)	C(15)	C(18)	113.1(5)	C(18)	N(3)	C(27)	120.4(5)
N(2)	C(16)	C(17)	117.0(5)	C(9)	O(1)	C(10)	121.2(5)
O(3)	C(16)	C(17)	118.6(5)	C(24)	O(5)	C(26)	106.4(5)
O(3)	C(16)	N(2)	124.4(6)	C(23)	O(6)	C(26)	106.1(5)

Table S7. Torsion angles for **12a**.

A	B	C	D	Angle/°	A	B	C	D	Angle/°
C(1)	C(2)	C(3)	C(4)	-179.7(7)	C(19)	C(20)	C(21)	C(22)	-177.5(6)
C(1)	C(2)	C(3)	C(8)	-1.6(7)	C(19)	C(20)	C(25)	C(24)	177.8(6)
C(1)	C(2)	C(14)	C(15)	89.7(8)	C(20)	C(19)	N(2)	C(15)	-103.7(6)
C(2)	C(1)	N(1)	C(8)	-0.3(7)	C(20)	C(19)	N(2)	C(16)	85.7(7)
C(2)	C(1)	N(1)	C(9)	-174.4(6)	C(20)	C(21)	C(22)	C(23)	0.8(11)
C(2)	C(3)	C(4)	C(5)	178.1(7)	C(21)	C(20)	C(25)	C(24)	1.0(10)
C(2)	C(3)	C(8)	C(7)	-179.6(6)	C(21)	C(22)	C(23)	C(24)	-1.4(11)
C(2)	C(3)	C(8)	N(1)	1.4(7)	C(21)	C(22)	C(23)	O(6)	179.4(7)
C(2)	C(14)	C(15)	C(18)	71.3(7)	C(22)	C(23)	C(24)	C(25)	1.9(11)
C(2)	C(14)	C(15)	N(2)	-55.3(7)	C(22)	C(23)	C(24)	O(5)	179.1(6)
C(3)	C(2)	C(14)	C(15)	-84.3(8)	C(22)	C(23)	O(6)	C(26)	178.6(8)
C(3)	C(4)	C(5)	C(6)	0.9(11)	C(23)	C(24)	C(25)	C(20)	-1.6(11)
C(3)	C(8)	N(1)	C(1)	-0.7(7)	C(23)	C(24)	O(5)	C(26)	3.1(8)
C(3)	C(8)	N(1)	C(9)	173.3(5)	C(24)	C(23)	O(6)	C(26)	-0.7(8)
C(4)	C(3)	C(8)	C(7)	-1.2(10)	C(25)	C(20)	C(21)	C(22)	-0.6(10)
C(4)	C(3)	C(8)	N(1)	179.9(6)	C(25)	C(24)	O(5)	C(26)	-179.9(8)
C(4)	C(5)	C(6)	C(7)	-1.0(11)	N(1)	C(1)	C(2)	C(3)	1.2(7)
C(5)	C(6)	C(7)	C(8)	0.0(11)	N(1)	C(1)	C(2)	C(14)	-173.8(6)
C(6)	C(7)	C(8)	C(3)	1.0(10)	N(1)	C(9)	O(1)	C(10)	178.9(5)
C(6)	C(7)	C(8)	N(1)	179.7(7)	N(2)	C(15)	C(18)	N(3)	26.2(8)
C(7)	C(8)	N(1)	C(1)	-179.6(6)	N(2)	C(15)	C(18)	O(4)	-154.7(6)
C(7)	C(8)	N(1)	C(9)	-5.5(11)	N(2)	C(16)	C(17)	N(3)	18.7(8)
C(8)	C(3)	C(4)	C(5)	0.2(10)	N(2)	C(19)	C(20)	C(21)	88.4(7)
C(11)	C(10)	O(1)	C(9)	63.7(8)	N(2)	C(19)	C(20)	C(25)	-88.4(7)
C(12)	C(10)	O(1)	C(9)	-61.0(8)	O(1)	C(9)	N(1)	C(1)	5.8(8)
C(13)	C(10)	O(1)	C(9)	-178.6(6)	O(1)	C(9)	N(1)	C(8)	-167.3(6)
C(14)	C(2)	C(3)	C(4)	-4.9(12)	O(2)	C(9)	N(1)	C(1)	-172.9(6)
C(14)	C(2)	C(3)	C(8)	173.2(6)	O(2)	C(9)	N(1)	C(8)	14.0(9)
C(14)	C(15)	C(18)	N(3)	-99.5(7)	O(2)	C(9)	O(1)	C(10)	-2.5(10)
C(14)	C(15)	C(18)	O(4)	79.6(7)	O(3)	C(16)	C(17)	N(3)	-161.1(5)
C(14)	C(15)	N(2)	C(16)	91.0(7)	O(3)	C(16)	N(2)	C(15)	-169.1(6)
C(14)	C(15)	N(2)	C(19)	-79.3(6)	O(3)	C(16)	N(2)	C(19)	1.0(9)
C(15)	C(18)	N(3)	C(17)	2.8(10)	O(4)	C(18)	N(3)	C(17)	-176.3(6)
C(15)	C(18)	N(3)	C(27)	175.0(6)	O(4)	C(18)	N(3)	C(27)	-4.1(11)
C(16)	C(17)	N(3)	C(18)	-26.1(9)	O(5)	C(24)	C(25)	C(20)	-178.3(7)
C(16)	C(17)	N(3)	C(27)	161.4(6)	O(5)	C(26)	O(6)	C(23)	2.6(9)
C(17)	C(16)	N(2)	C(15)	11.1(9)	O(6)	C(23)	C(24)	C(25)	-178.7(6)
C(17)	C(16)	N(2)	C(19)	-178.8(5)	O(6)	C(23)	C(24)	O(5)	-1.5(8)

C(18)	C(15)	N(2)	C(16)	-34.0(8)	O(6)	C(26)	O(5)	C(24)	-3.5(9)
C(18)	C(15)	N(2)	C(19)	155.8(5)					

Table S8. Bond lengths for **6a**.

**Atom**	**Atom**	**Length/Å**	**Atom**	**Atom**	**Length/Å**
C(1)	C(2)	1.370(4)	C(12)	N(3)	1.449(4)
C(1)	N(1)	1.373(4)	C(13)	N(3)	1.342(4)
C(2)	C(3)	1.437(4)	C(13)	O(2)	1.241(4)
C(2)	C(9)	1.508(4)	C(14)	N(3)	1.460(4)
C(3)	C(4)	1.404(4)	C(15)	C(16)	1.511(4)
C(3)	C(8)	1.415(4)	C(15)	N(2)	1.473(4)
C(4)	C(5)	1.373(5)	C(16)	C(17)	1.401(4)
C(5)	C(6)	1.402(4)	C(16)	C(21)	1.387(5)
C(6)	C(7)	1.377(5)	C(17)	C(18)	1.374(5)
C(7)	C(8)	1.404(5)	C(18)	C(19)	1.387(4)
C(8)	N(1)	1.380(4)	C(18)	O(3)	1.385(4)
C(9)	C(10)	1.560(4)	C(19)	C(20)	1.360(5)
C(10)	C(13)	1.511(4)	C(19)	O(4)	1.384(4)
C(10)	N(2)	1.460(4)	C(20)	C(21)	1.394(5)
C(11)	C(12)	1.507(5)	C(22)	O(3)	1.437(4)
C(11)	N(2)	1.346(4)	C(22)	O(4)	1.440(4)
C(11)	O(1)	1.238(4)			

Table S9. Valence angles for **6a**.

**Atom**	**Atom**	**Atom**	**Angle/°**	**Atom**	**Atom**	**Atom**	**Angle/°**
C(2)	C(1)	N(1)	109.9(3)	O(2)	C(13)	N(3)	122.7(3)
C(1)	C(2)	C(3)	106.9(3)	C(1)	N(1)	C(8)	108.9(3)
C(1)	C(2)	C(9)	125.3(3)	N(2)	C(15)	C(16)	113.0(2)
C(3)	C(2)	C(9)	127.6(3)	C(17)	C(16)	C(15)	119.3(3)
C(4)	C(3)	C(2)	134.9(3)	C(21)	C(16)	C(15)	120.6(3)
C(4)	C(3)	C(8)	118.6(3)	C(21)	C(16)	C(17)	120.0(3)
C(8)	C(3)	C(2)	106.5(3)	C(18)	C(17)	C(16)	117.3(3)
C(5)	C(4)	C(3)	119.7(3)	C(17)	C(18)	C(19)	122.1(3)
C(4)	C(5)	C(6)	120.6(3)	C(17)	C(18)	O(3)	128.4(3)
C(7)	C(6)	C(5)	121.9(3)	O(3)	C(18)	C(19)	109.4(3)
C(6)	C(7)	C(8)	117.2(3)	C(20)	C(19)	C(18)	121.4(3)
C(7)	C(8)	C(3)	122.0(3)	C(20)	C(19)	O(4)	128.9(3)
N(1)	C(8)	C(3)	107.7(3)	O(4)	C(19)	C(18)	109.6(3)
N(1)	C(8)	C(7)	130.3(3)	C(19)	C(20)	C(21)	117.4(3)
C(2)	C(9)	C(10)	113.0(2)	C(16)	C(21)	C(20)	121.9(3)
C(13)	C(10)	C(9)	109.4(2)	O(3)	C(22)	O(4)	106.9(3)
N(2)	C(10)	C(9)	112.0(2)	C(10)	N(2)	C(15)	116.4(2)
N(2)	C(10)	C(13)	114.0(2)	C(11)	N(2)	C(10)	121.9(3)
N(2)	C(11)	C(12)	118.2(3)	C(11)	N(2)	C(15)	120.0(3)
O(1)	C(11)	C(12)	118.7(3)	C(12)	N(3)	C(14)	115.7(3)
O(1)	C(11)	N(2)	123.2(3)	C(13)	N(3)	C(12)	122.7(3)
N(3)	C(12)	C(11)	116.1(3)	C(13)	N(3)	C(14)	121.2(3)
N(3)	C(13)	C(10)	118.2(3)	C(18)	O(3)	C(22)	103.9(3)
O(2)	C(13)	C(10)	119.0(3)	C(19)	O(4)	C(22)	103.6(3)

Table S10. Torsion angles for **6a**.

A	B	C	D	Angle/°	A	B	C	D	Angle/°
C(1)	C(2)	C(3)	C(4)	-179.7(3)	N(1)	C(1)	C(2)	C(3)	0.3(4)
C(1)	C(2)	C(3)	C(8)	-0.1(3)	N(1)	C(1)	C(2)	C(9)	-174.9(3)
C(1)	C(2)	C(9)	C(10)	90.9(4)	C(15)	C(16)	C(17)	C(18)	176.9(3)
C(2)	C(1)	N(1)	C(8)	-0.3(4)	C(15)	C(16)	C(21)	C(20)	-177.6(3)
C(2)	C(3)	C(4)	C(5)	178.2(3)	C(16)	C(15)	N(2)	C(10)	-81.3(3)
C(2)	C(3)	C(8)	C(7)	-178.8(3)	C(16)	C(15)	N(2)	C(11)	113.2(3)
C(2)	C(3)	C(8)	N(1)	-0.1(3)	C(16)	C(17)	C(18)	C(19)	1.2(5)
C(2)	C(9)	C(10)	C(13)	79.3(3)	C(16)	C(17)	C(18)	O(3)	176.4(3)
C(2)	C(9)	C(10)	N(2)	-48.1(3)	C(17)	C(16)	C(21)	C(20)	-0.2(5)
C(3)	C(2)	C(9)	C(10)	-83.3(4)	C(17)	C(18)	C(19)	C(20)	-1.2(5)
C(3)	C(4)	C(5)	C(6)	1.1(5)	C(17)	C(18)	C(19)	O(4)	175.3(3)
C(3)	C(8)	N(1)	C(1)	0.3(4)	C(17)	C(18)	O(3)	C(22)	169.4(3)
C(4)	C(3)	C(8)	C(7)	0.8(5)	C(18)	C(19)	C(20)	C(21)	0.5(5)
C(4)	C(3)	C(8)	N(1)	179.5(3)	C(18)	C(19)	O(4)	C(22)	16.0(3)
C(4)	C(5)	C(6)	C(7)	-0.3(5)	C(19)	C(18)	O(3)	C(22)	-14.9(3)
C(5)	C(6)	C(7)	C(8)	-0.2(5)	C(19)	C(20)	C(21)	C(16)	0.2(5)
C(6)	C(7)	C(8)	C(3)	0.0(5)	C(20)	C(19)	O(4)	C(22)	-167.9(3)
C(6)	C(7)	C(8)	N(1)	-178.5(3)	C(21)	C(16)	C(17)	C(18)	-0.5(4)
C(7)	C(8)	N(1)	C(1)	178.8(3)	N(2)	C(10)	C(13)	N(3)	25.6(4)
C(8)	C(3)	C(4)	C(5)	-1.3(5)	N(2)	C(10)	C(13)	O(2)	-156.8(3)
C(9)	C(2)	C(3)	C(4)	-4.6(6)	N(2)	C(11)	C(12)	N(3)	14.2(4)
C(9)	C(2)	C(3)	C(8)	174.9(3)	N(2)	C(15)	C(16)	C(17)	129.3(3)
C(9)	C(10)	C(13)	N(3)	-100.7(3)	N(2)	C(15)	C(16)	C(21)	-53.3(4)
C(9)	C(10)	C(13)	O(2)	76.9(3)	O(1)	C(11)	C(12)	N(3)	-164.4(3)
C(9)	C(10)	N(2)	C(11)	93.9(3)	O(1)	C(11)	N(2)	C(10)	-170.3(3)
C(9)	C(10)	N(2)	C(15)	-71.2(3)	O(1)	C(11)	N(2)	C(15)	-5.7(5)
C(10)	C(13)	N(3)	C(12)	-1.3(4)	O(2)	C(13)	N(3)	C(12)	-178.8(3)
C(10)	C(13)	N(3)	C(14)	171.5(3)	O(2)	C(13)	N(3)	C(14)	-6.0(4)
C(11)	C(12)	N(3)	C(13)	-19.1(4)	O(3)	C(18)	C(19)	C(20)	-177.3(3)
C(11)	C(12)	N(3)	C(14)	167.7(3)	O(3)	C(18)	C(19)	O(4)	-0.7(4)
C(12)	C(11)	N(2)	C(10)	11.2(4)	O(3)	C(22)	O(4)	C(19)	-25.1(3)
C(12)	C(11)	N(2)	C(15)	175.8(3)	O(4)	C(19)	C(20)	C(21)	-175.3(3)
C(13)	C(10)	N(2)	C(11)	-31.0(4)	O(4)	C(22)	O(3)	C(18)	24.7(3)
C(13)	C(10)	N(2)	C(15)	163.9(2)					

Table S11. Bond lengths for **1**.

Atom	Atom	Length/Å	Atom	Atom	Length/Å
C(1)	C(14)	1.493(4)	C(9)	C(10)	1.388(5)
C(1)	C(15)	1.527(4)	C(10)	C(11)	1.401(5)
C(1)	N(1)	1.483(4)	C(11)	C(12)	1.386(5)
C(2)	C(3)	1.502(4)	C(12)	C(13)	1.391(5)
C(2)	N(1)	1.348(4)	C(13)	N(3)	1.384(4)
C(2)	O(3)	1.234(4)	C(14)	N(3)	1.377(4)
C(3)	N(2)	1.459(4)	C(15)	C(16)	1.387(4)
C(4)	C(5)	1.537(4)	C(15)	C(20)	1.403(4)
C(4)	N(2)	1.346(4)	C(16)	C(17)	1.403(4)

C(4)	O(4)	1.227(4)	C(17)	C(18)	1.374(4)
C(5)	C(6)	1.532(4)	C(18)	C(19)	1.382(4)
C(5)	N(1)	1.492(4)	C(18)	O(1)	1.383(4)
C(6)	C(7)	1.498(4)	C(19)	C(20)	1.368(4)
C(7)	C(8)	1.441(4)	C(19)	O(2)	1.379(4)
C(7)	C(14)	1.361(4)	C(21)	O(1)	1.420(4)
C(8)	C(9)	1.401(5)	C(21)	O(2)	1.431(4)
C(8)	C(13)	1.420(4)	C(22)	N(2)	1.462(4)

Table S12. Valence angles for **1**.

Atom	Atom	Atom	Angle/°	Atom	Atom	Atom	Angle/°
C(14)	C(1)	C(15)	108.5(2)	N(3)	C(13)	C(12)	129.9(3)
N(1)	C(1)	C(14)	109.1(2)	C(7)	C(14)	C(1)	124.9(3)
N(1)	C(1)	C(15)	112.5(2)	C(7)	C(14)	N(3)	111.0(3)
N(1)	C(2)	C(3)	117.4(3)	N(3)	C(14)	C(1)	123.8(3)
O(3)	C(2)	C(3)	119.7(3)	C(16)	C(15)	C(1)	121.7(3)
O(3)	C(2)	N(1)	122.8(3)	C(16)	C(15)	C(20)	120.5(3)
N(2)	C(3)	C(2)	114.2(3)	C(20)	C(15)	C(1)	117.5(3)
N(2)	C(4)	C(5)	117.0(3)	C(15)	C(16)	C(17)	121.7(3)
O(4)	C(4)	C(5)	119.8(3)	C(18)	C(17)	C(16)	116.6(3)
O(4)	C(4)	N(2)	123.1(3)	C(17)	C(18)	C(19)	121.7(3)
C(6)	C(5)	C(4)	109.3(2)	C(17)	C(18)	O(1)	128.4(3)
N(1)	C(5)	C(4)	112.4(2)	C(19)	C(18)	O(1)	109.9(3)
N(1)	C(5)	C(6)	112.1(2)	C(20)	C(19)	C(18)	122.4(3)
C(7)	C(6)	C(5)	108.0(2)	C(20)	C(19)	O(2)	127.7(3)
C(8)	C(7)	C(6)	133.4(3)	O(2)	C(19)	C(18)	109.9(3)
C(14)	C(7)	C(6)	119.8(3)	C(19)	C(20)	C(15)	117.0(3)
C(14)	C(7)	C(8)	106.7(3)	O(1)	C(21)	O(2)	108.9(3)
C(9)	C(8)	C(7)	134.5(3)	C(1)	N(1)	C(5)	121.5(2)
C(9)	C(8)	C(13)	119.1(3)	C(2)	N(1)	C(1)	115.8(2)
C(13)	C(8)	C(7)	106.4(3)	C(2)	N(1)	C(5)	121.3(3)
C(10)	C(9)	C(8)	118.7(3)	C(3)	N(2)	C(22)	118.3(3)
C(9)	C(10)	C(11)	121.1(3)	C(4)	N(2)	C(3)	121.9(3)
C(12)	C(11)	C(10)	121.4(3)	C(4)	N(2)	C(22)	119.0(3)
C(11)	C(12)	C(13)	117.5(3)	C(14)	N(3)	C(13)	107.9(3)
C(12)	C(13)	C(8)	122.0(3)	C(18)	O(1)	C(21)	105.1(2)
N(3)	C(13)	C(8)	108.0(3)	C(19)	O(2)	C(21)	105.1(2)

Table S13. Torsion angles for **1**.

A	B	C	D	Angle/°	A	B	C	D	Angle/°
C(1)	C(14)	N(3)	C(13)	-173.3(3)	C(14)	C(1)	N(1)	C(5)	-3.5(4)
C(1)	C(15)	C(16)	C(17)	-174.1(3)	C(14)	C(7)	C(8)	C(9)	-174.8(4)
C(1)	C(15)	C(20)	C(19)	173.1(3)	C(14)	C(7)	C(8)	C(13)	1.7(3)
C(2)	C(3)	N(2)	C(4)	33.8(4)	C(15)	C(1)	C(14)	C(7)	-93.4(3)
C(2)	C(3)	N(2)	C(22)	-156.5(3)	C(15)	C(1)	C(14)	N(3)	78.6(4)
C(3)	C(2)	N(1)	C(1)	-172.1(3)	C(15)	C(1)	N(1)	C(2)	-76.4(3)
C(3)	C(2)	N(1)	C(5)	-5.4(4)	C(15)	C(1)	N(1)	C(5)	117.0(3)
C(4)	C(5)	C(6)	C(7)	178.0(2)	C(15)	C(16)	C(17)	C(18)	0.9(5)
C(4)	C(5)	N(1)	C(1)	-160.6(3)	C(16)	C(15)	C(20)	C(19)	-0.7(4)

C(4)	C(5)	N(1)	C(2)	33.5(4)	C(16)	C(17)	C(18)	C(19)	-0.1(5)
C(5)	C(4)	N(2)	C(3)	-4.9(4)	C(16)	C(17)	C(18)	O(1)	-178.4(3)
C(5)	C(4)	N(2)	C(22)	-174.5(3)	C(17)	C(18)	C(19)	C(20)	-1.2(5)
C(5)	C(6)	C(7)	C(8)	144.6(3)	C(17)	C(18)	C(19)	O(2)	-179.4(3)
C(5)	C(6)	C(7)	C(14)	-31.3(4)	C(17)	C(18)	O(1)	C(21)	-174.5(3)
C(6)	C(5)	N(1)	C(1)	-36.9(4)	C(18)	C(19)	C(20)	C(15)	1.6(5)
C(6)	C(5)	N(1)	C(2)	157.2(3)	C(18)	C(19)	O(2)	C(21)	-5.8(3)
C(6)	C(7)	C(8)	C(9)	9.0(6)	C(19)	C(18)	O(1)	C(21)	6.9(4)
C(6)	C(7)	C(8)	C(13)	-174.6(3)	C(20)	C(15)	C(16)	C(17)	-0.5(5)
C(6)	C(7)	C(14)	C(1)	-11.1(5)	C(20)	C(19)	O(2)	C(21)	176.2(3)
C(6)	C(7)	C(14)	N(3)	176.1(3)	N(1)	C(1)	C(14)	C(7)	29.5(4)
C(7)	C(8)	C(9)	C(10)	178.9(4)	N(1)	C(1)	C(14)	N(3)	-158.5(3)
C(7)	C(8)	C(13)	C(12)	177.8(3)	N(1)	C(1)	C(15)	C(16)	-32.4(4)
C(7)	C(8)	C(13)	N(3)	-1.9(3)	N(1)	C(1)	C(15)	C(20)	153.9(3)
C(7)	C(14)	N(3)	C(13)	-0.4(4)	N(1)	C(2)	C(3)	N(2)	-28.1(4)
C(8)	C(7)	C(14)	C(1)	172.0(3)	N(1)	C(5)	C(6)	C(7)	52.6(3)
C(8)	C(7)	C(14)	N(3)	-0.8(4)	N(2)	C(4)	C(5)	C(6)	-153.1(3)
C(8)	C(9)	C(10)	C(11)	1.0(6)	N(2)	C(4)	C(5)	N(1)	-27.9(4)
C(8)	C(13)	N(3)	C(14)	1.5(3)	O(1)	C(18)	C(19)	C(20)	177.4(3)
C(9)	C(8)	C(13)	C(12)	-5.0(5)	O(1)	C(18)	C(19)	O(2)	-0.7(4)
C(9)	C(8)	C(13)	N(3)	175.2(3)	O(1)	C(21)	O(2)	C(19)	10.1(4)
C(9)	C(10)	C(11)	C(12)	-3.0(6)	O(2)	C(19)	C(20)	C(15)	179.4(3)
C(10)	C(11)	C(12)	C(13)	0.8(5)	O(2)	C(21)	O(1)	C(18)	-10.5(4)
C(11)	C(12)	C(13)	C(8)	3.2(5)	O(3)	C(2)	C(3)	N(2)	153.9(3)
C(11)	C(12)	C(13)	N(3)	-177.1(3)	O(3)	C(2)	N(1)	C(1)	5.8(4)
C(12)	C(13)	N(3)	C(14)	-178.3(3)	O(3)	C(2)	N(1)	C(5)	172.5(3)
C(13)	C(8)	C(9)	C(10)	2.8(5)	O(4)	C(4)	C(5)	C(6)	28.8(4)
C(14)	C(1)	C(15)	C(16)	88.4(3)	O(4)	C(4)	C(5)	N(1)	154.0(3)
C(14)	C(1)	C(15)	C(20)	-85.3(3)	O(4)	C(4)	N(2)	C(3)	173.2(3)
C(14)	C(1)	N(1)	C(2)	163.2(2)	O(4)	C(4)	N(2)	C(22)	3.6(5)

Table S14. The geometry of hydrogen bonds in the crystal of **11a**.

D–H	A	D−H (Å)	d(D···A) (Å)	< D–H···A (°)
N2A–H2A	O4B	0.87(4)	2.919(6)	154(7)
N2B–H2B	$O4A^{i}$	0.87(4)	2.908(6)	157(6)
C4A–H4A	O4B	0.93	3.459(7)	163
C7A–H7A	O2B*	0.93	2.901(6)	115
C7B–H7B	O2B*	0.93	2.924(7)	115
C12A–H12A	O2A*	0.96	3.016(7)	119
C12B–H12D	O2B*	0.96	3.050(7)	114
C13A–H13C	O2A*	0.96	2.979(7)	115
C13B–H13F	O2B*	0.96	2.956(8)	116
C15A–H15A	$O4A^{i}$	0.98	3.246(6)	143
C15B–H15B	$O4B^{i}$	0.98	3.451(6)	162
C17A–H17A	$O3A^{ii}$	0.97	3.229(7)	140
C17B–H17C	$O3A^{iii}$	0.97	3.401(6)	158

Symmetry codes: (i) *x* + 1, *y*, *z*; (ii) *x* – 1, *y*, *z*; (iii) *x*, *y*, *z* +1; (*) intramolecular interaction.

Table S15. The geometry of C–H···π contacts in the crystal of **11a**.

D–H	*Cg*I	d(D··· *Cg*I) (Å)	< D–X···*Cg*I (°)
C12A−H12B	5^{iv}	3.490(6)	122

C12B−H12E	1^{v}	3.540(6)	126
C17A−H17B	1*	3.376(6)	131

Symmetry code: (iv) $-x + 1, y + 1/2, -z$; (v) $-x + 1, y - 1/2, -z + 1$; (*) intramolecular interaction.
*Cg*1 and *Cg*5 denote the geometric centres of gravity of the aromatic rings delineated by the N1A/C1A−C3A/C8A and N1B/C1B−C3B/C8B atoms, respectively (Fig. 2a).

Table S16. The geometry of C=O···*π* contacts in the crystal of **11a**.

Y=X	*Cg*I	d(X··· *Cg*I) (Å)	< Y=X···*Cg*I (°)
C18A=O4A	3*	3.951(4)	77.6(3)

Symmetry code: (*) intramolecular interaction.
*Cg*3 denotes the geometric centre of gravity of the aromatic ring delineated by the C3A−C8A atoms (Fig. 2a).

Table S17. The geometry of hydrogen bonds in the crystal of **12a**.

D–H	A	D−H (Å)	d(D···A) (Å)	< D–H···A (°)
C7–H7	O2*	0.93	2.986(9)	115
C11–H11A	O2*	0.96	3.048(9)	118
C12–H12C	O2*	0.96	3.032(9)	117
C15–H15	$O3^{i}$	0.98	3.452(7)	146
C21–H21	$O3^{i}$	0.93	3.418(7)	163
C26–H26A	$O2^{ii}$	0.97	2.987(8)	117

Symmetry codes: (i) $x - 1, y, z$; (ii) $-x + 3/2, -y + 1, z - 1/2$; (*) intramolecular interaction.

Table S18. The geometry of C–H···*π* contacts in the crystal of **12a**.

D–H	*Cg*I	d(D··· *Cg*I) (Å)	< D–X···*Cg*I (°)
C13−H13C	5^{iii}	3.896(7)	170
C17−H17B	2*	3.361(8)	130
C26−H26B	2^{iv}	3.362(9)	135

Symmetry code: (iii) $-x + 1, y + 1/2, -z + 3/2$; (iv) $-x + 1/2, -y + 1, -z + 3/2$; (*) intramolecular interaction.
*Cg*2 and *Cg*5 denote the geometric centres of gravity of the aromatic rings delineated by the N1/C1−C3/C8 and C20−C25 atoms, respectively (Fig. 4).

Table S19. The geometry of hydrogen bonds in the crystal of **6a**.

D–H	A	D−H (Å)	d(D···A) (Å)	< D–H···A (°)
N1–H1A	$O2^{i}$	0.88(4)	2.895(3)	171(3)
C9–H9	$O1^{ii}$	0.97	3.385(4)	155
C15–H15B	$O1^{ii}$	0.97	3.345(4)	171
C22–H22B	$O1^{iii}$	0.97	3.254(4)	170

Symmetry codes: (i) $-x + 1/2, -y + 1, z - 1/2$; (ii) $-x + 1, y - 1/2, -z + 1/2$; (iii) $-x + 3/2, -y + 1, z +1/2$; (*) intramolecular interaction.

Table S20. The geometry of C–H···*π* contacts in the crystal of **6a**.

D–H	*Cg*I	d(D··· *Cg*I) (Å)	< D–X···*Cg*I (°)
C12−H12B	2*	3.554(4)	122
C14−H14B	4^{iv}	3.451(4)	127

Symmetry code: (iv) $-x, y + 1/2, -z + 1/2$;(*) intramolecular interaction.
*Cg*2 and *Cg*4 denote the geometric centres of gravity of the aromatic rings delineated by the N1/C1−C3/C8 and C3−C8 atoms, respectively (Fig. 6).

Table S21. The geometry of hydrogen bonds in the crystal of **1**.

D–H	A	D−H (Å)	d(D···A) (Å)	< D–H···A (°)
N3–H3	$O3^{i}$	0.83(4)	2.968(3)	171(4)
C9–H9	$O4^{ii}$	0.93	3.476(5)	165
C16–H16	$O1^{iii}$	0.93	3.176(4)	151
C20–H20	$O3^{i}$	0.93	3.373(4)	154

Symmetry codes: (i) $-x + 1, y + 1/2, -z +1$; (ii) $-x + 2, y + 1/2, -z + 2$; (iii) $-x + 2, y - 1/2, -z + 1$; (*) intramolecular interaction.

Table S22. The geometry of C–H···*π* contacts in the crystal of **1**.

D–H	*Cg*I	d(D··· *Cg*I) (Å)	< D–X···*Cg*I (°)

C3−H3A	2^{iv}	3.550(3)	167
C3−H3B	6^{iv}	3.690(3)	139

Symmetry code: (iv) *x*, *y* − 1, *z*.

*Cg*2 and *Cg*6 denote the geometric centres of gravity of the aromatic rings delineated by the N3/C13/C8−C7/C14 and C15−C20 atoms, respectively (Fig. 8).

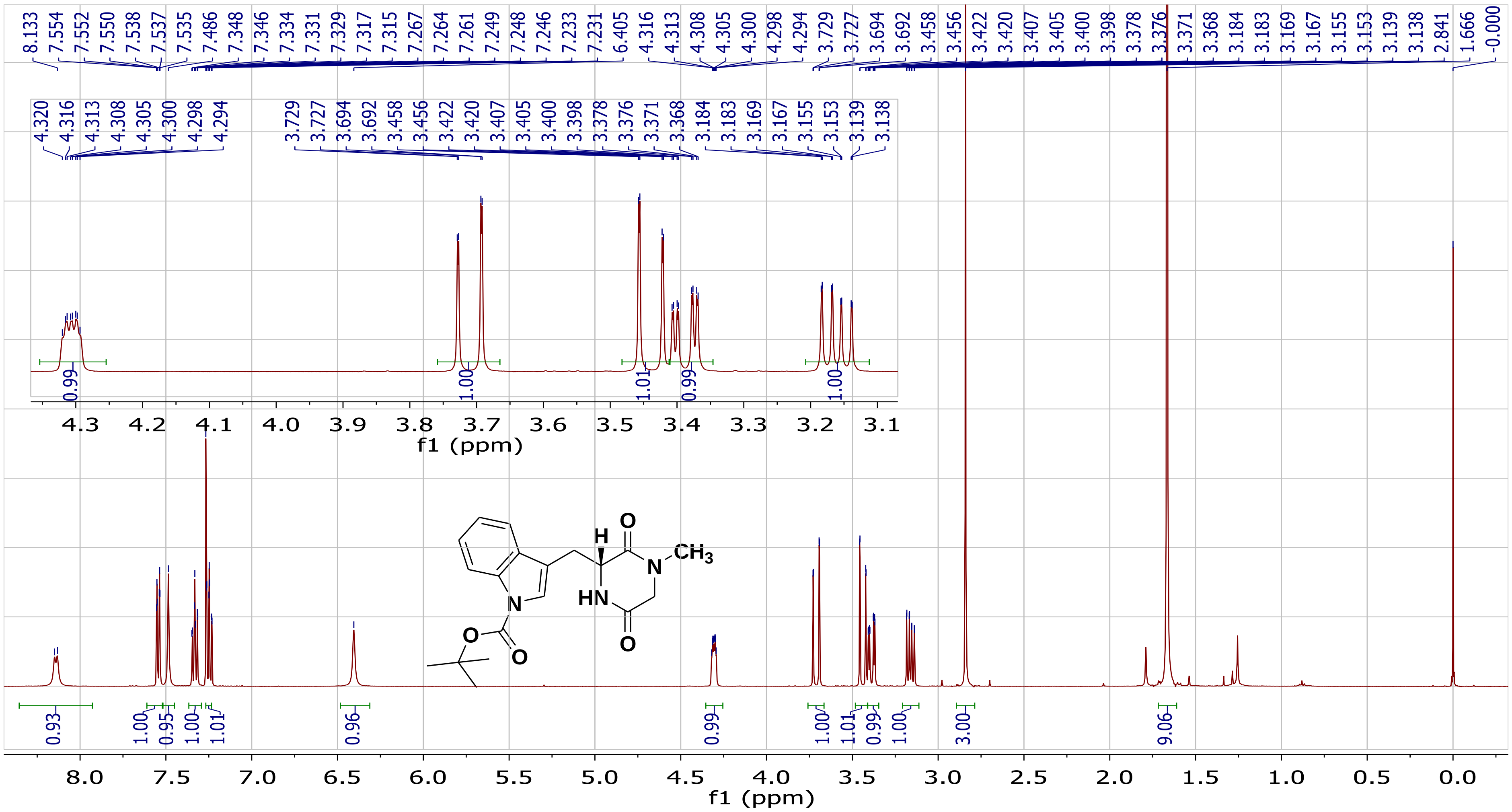

Figure S1. 1H-NMR spectrum for **11a**.

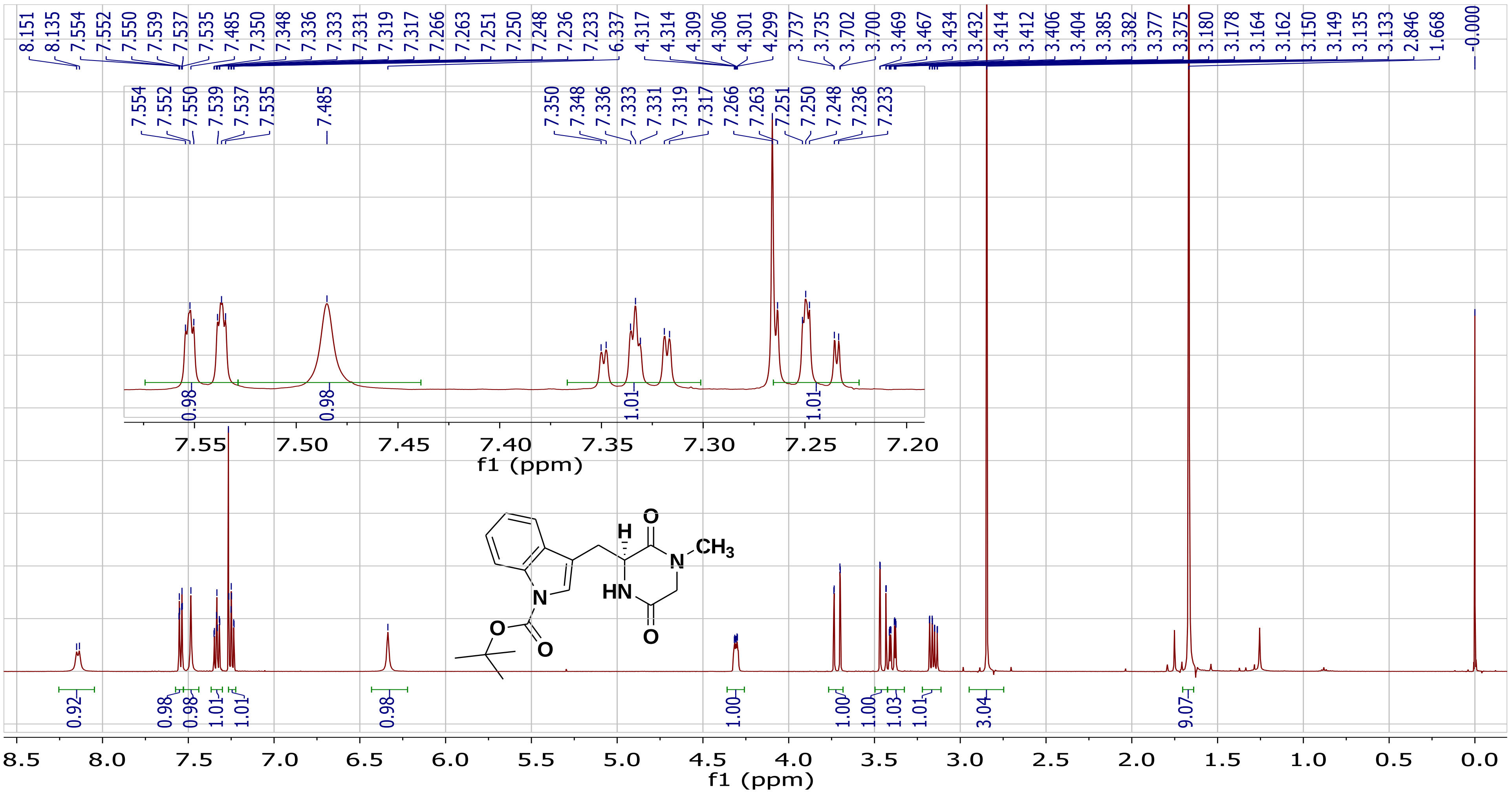

Figure S2. 1H-NMR spectrum for **11b**.

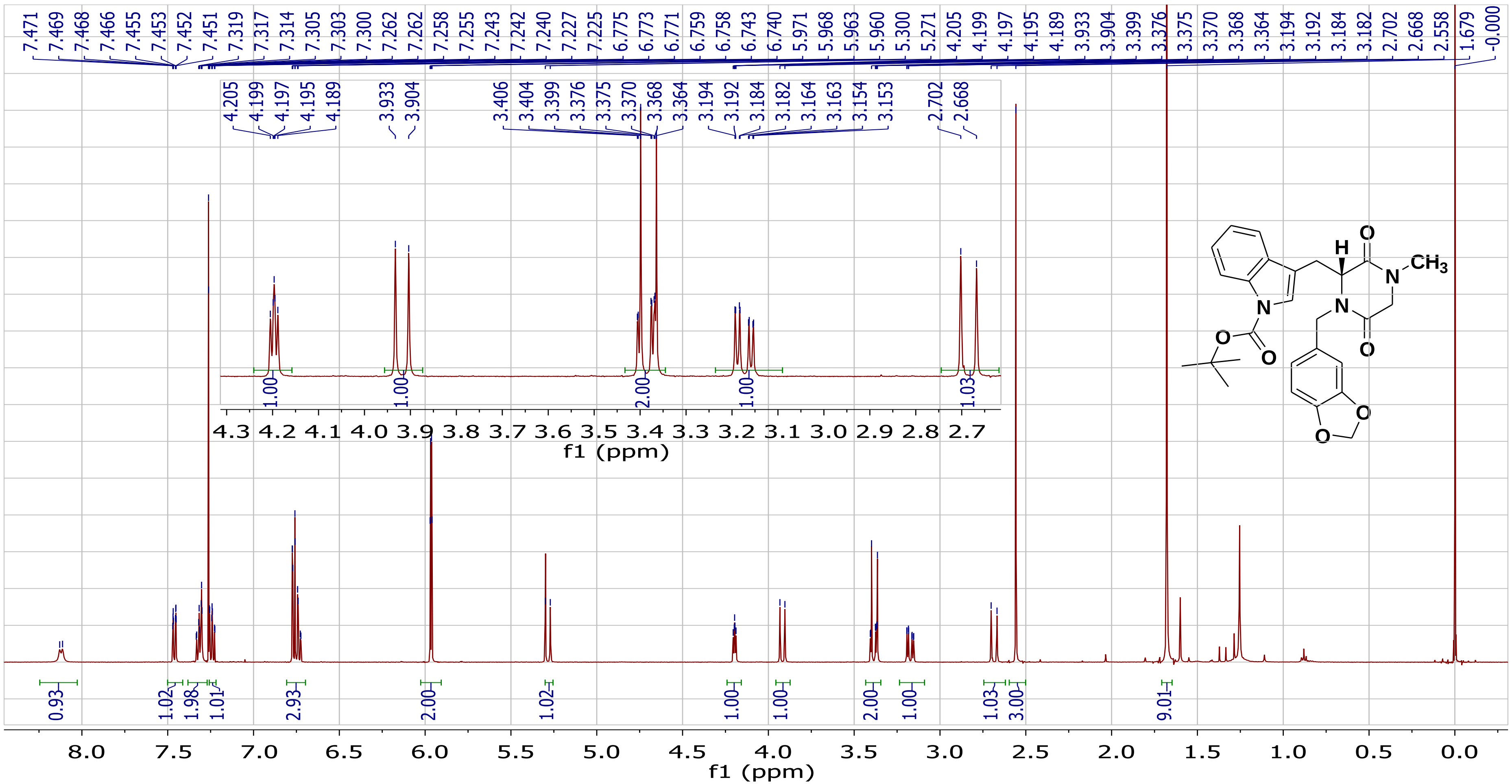

Figure S3. 1H-NMR spectrum for **12a**.

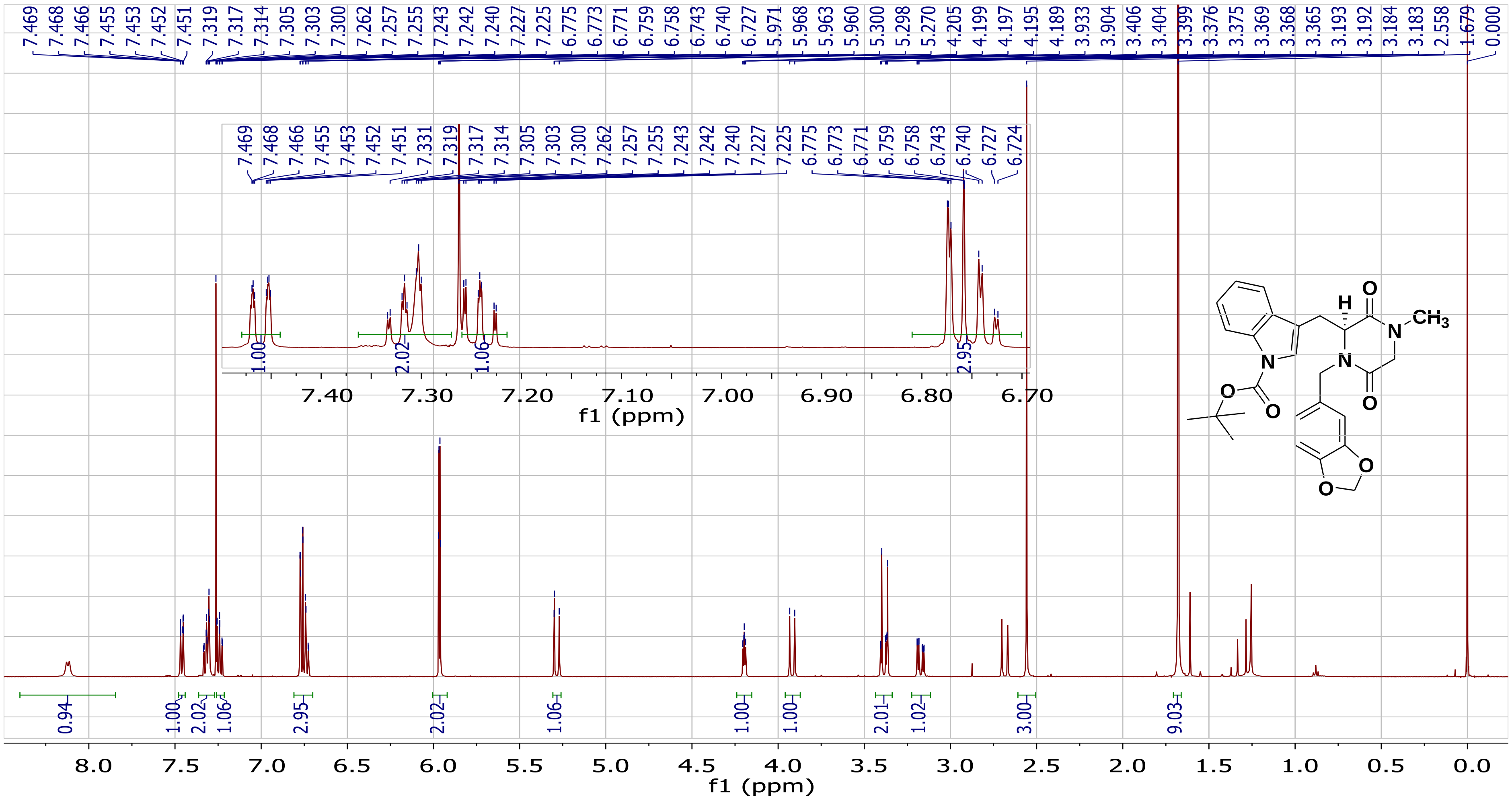

Figure S4. 1H-NMR spectrum for **12b**.

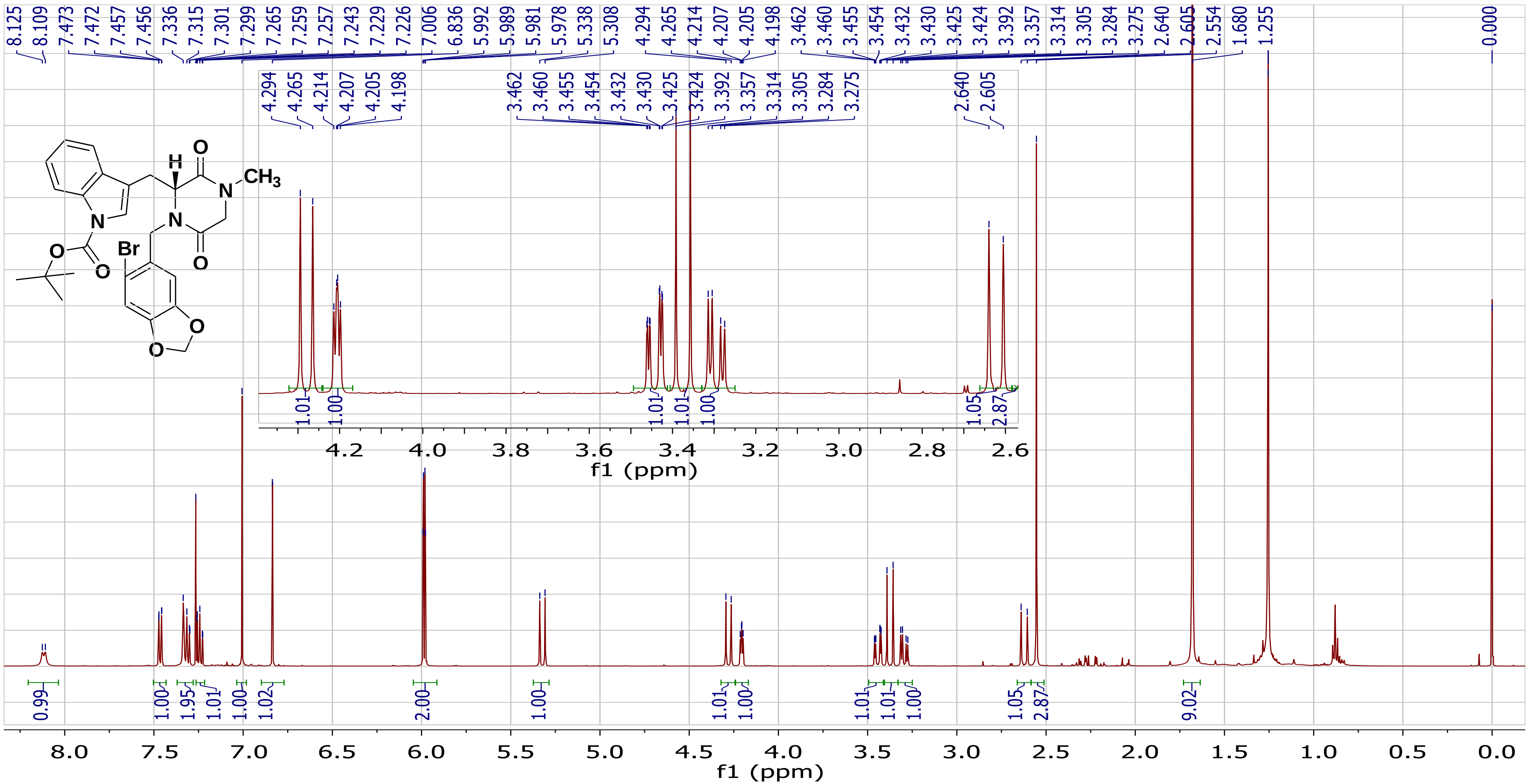

Figure S5. 1H-NMR spectrum for **13a**.

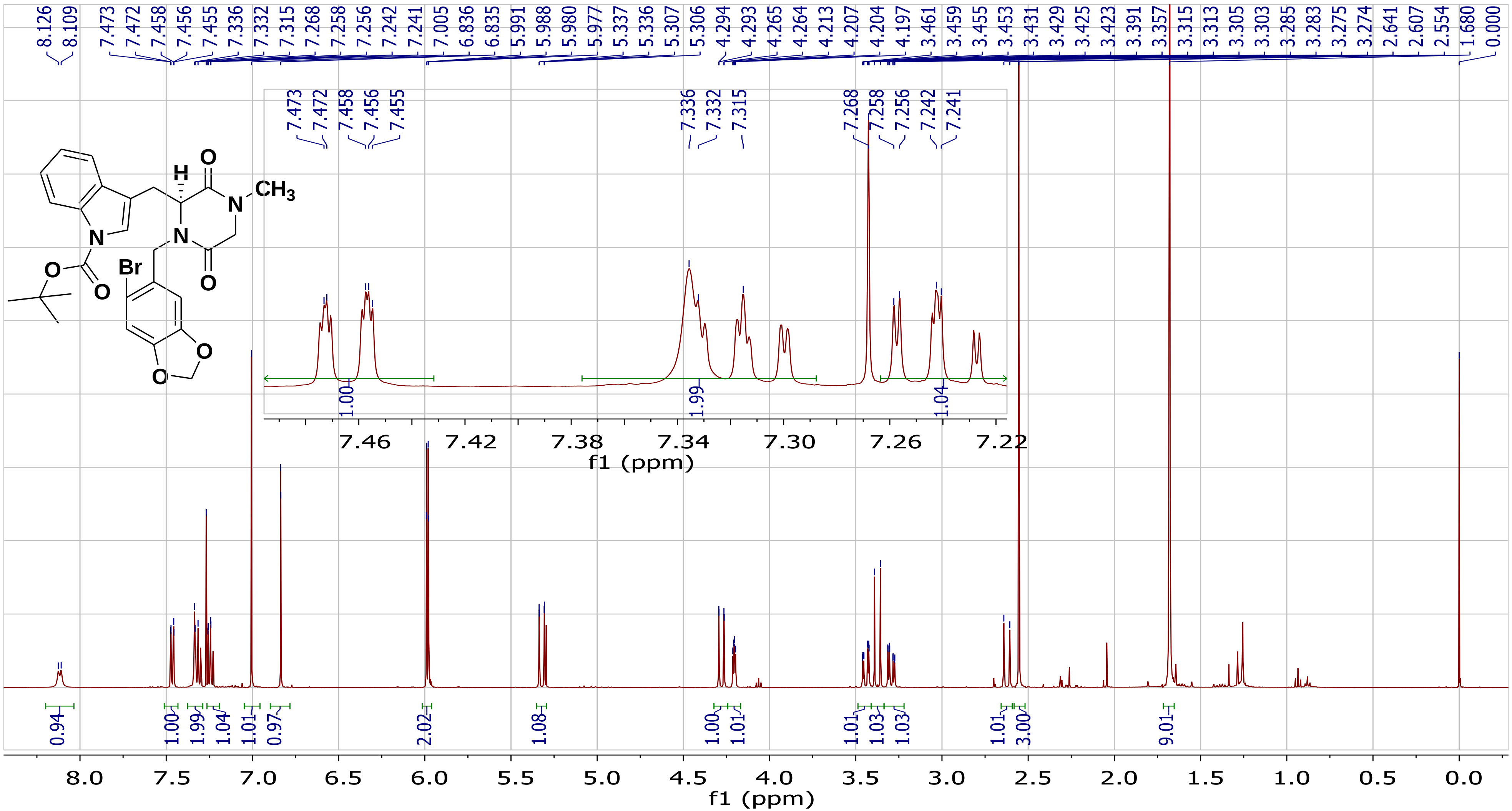

Figure S6. 1H-NMR spectrum for **13b**.

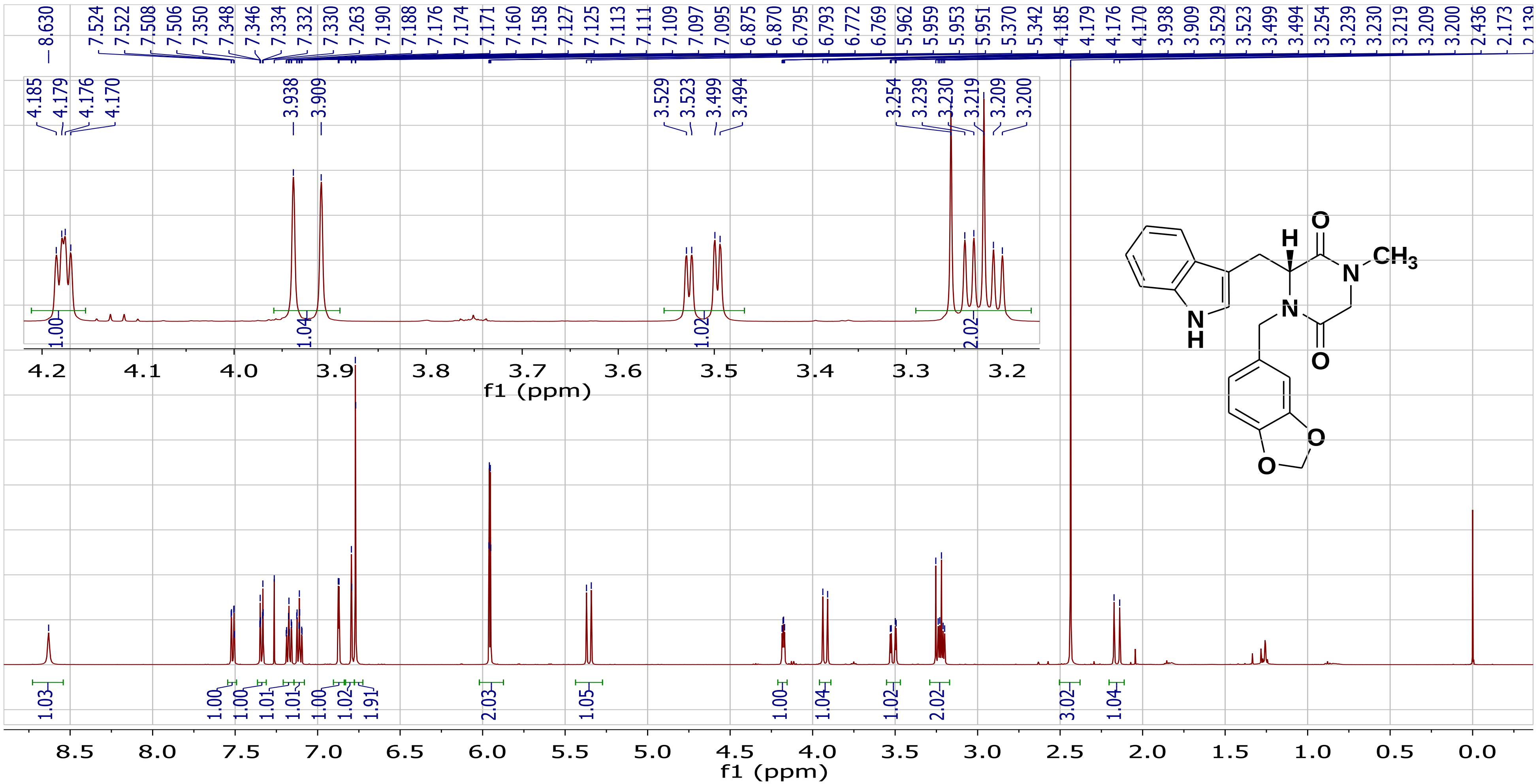

Figure S7. 1H-NMR spectrum for **6a**.

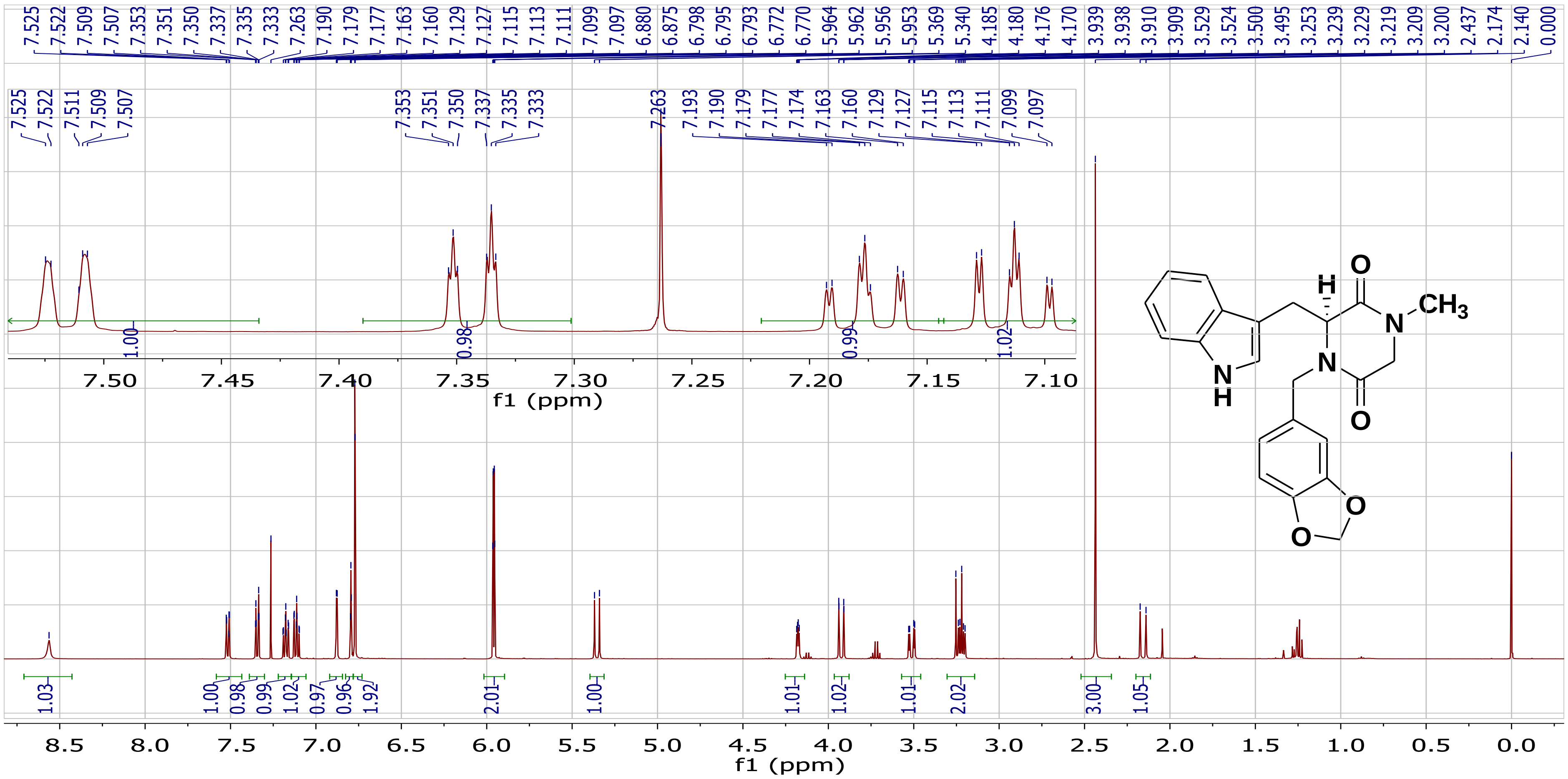

Figure S8. 1H-NMR spectrum for **6b**.

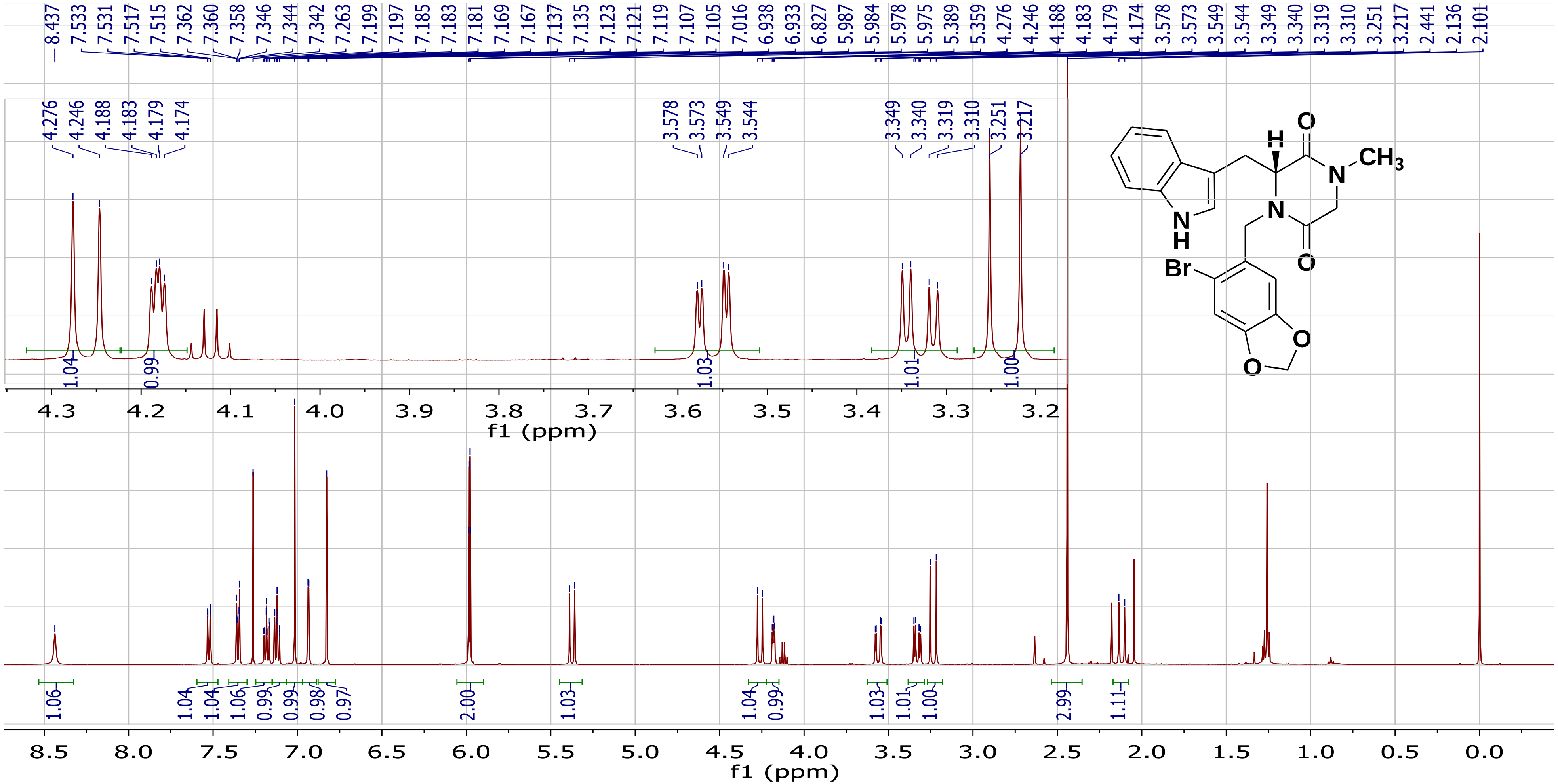

Figure S9. 1H-NMR spectrum for **7a**.

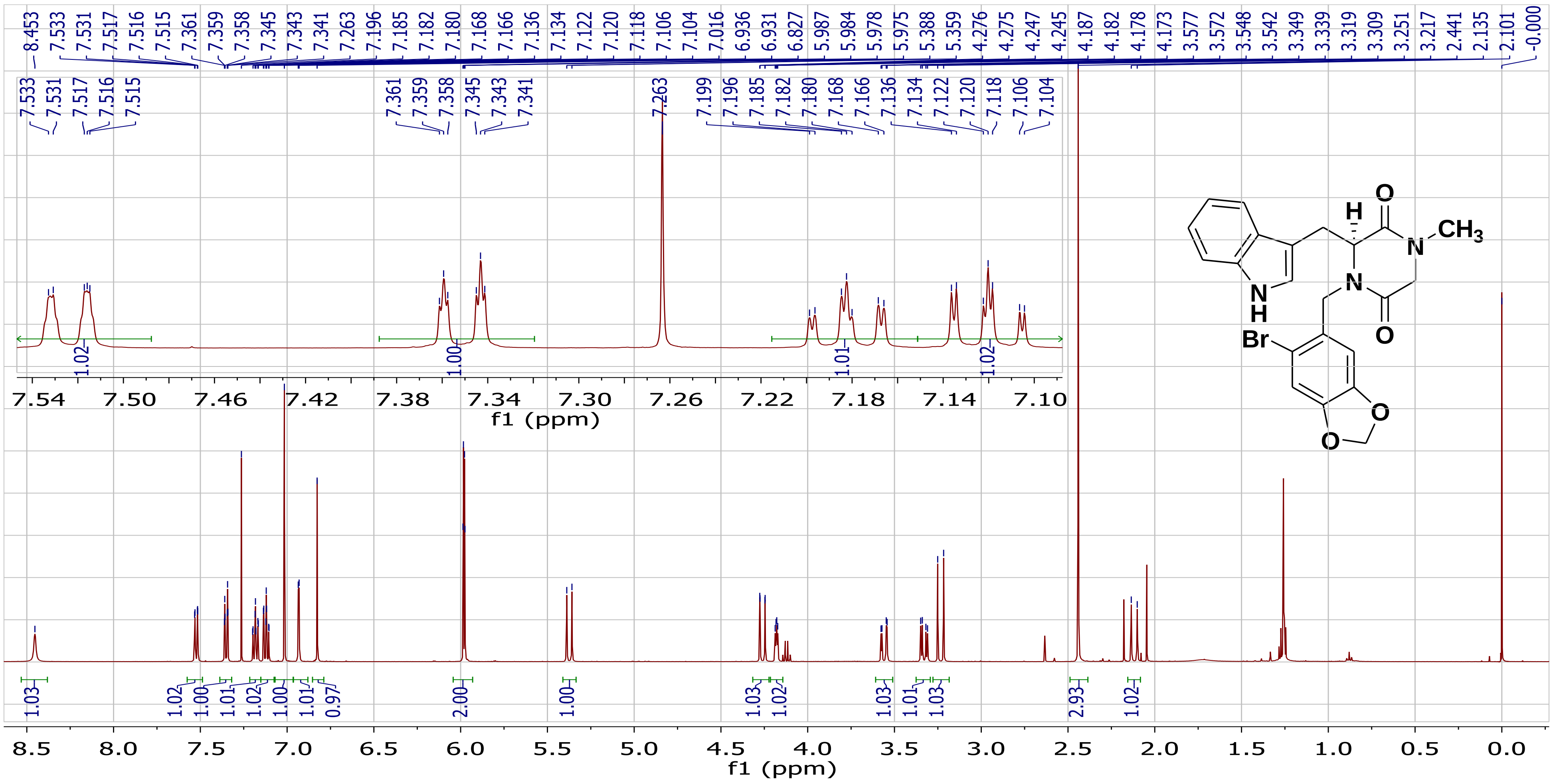

Figure S10. 1H-NMR spectrum for **7b**.

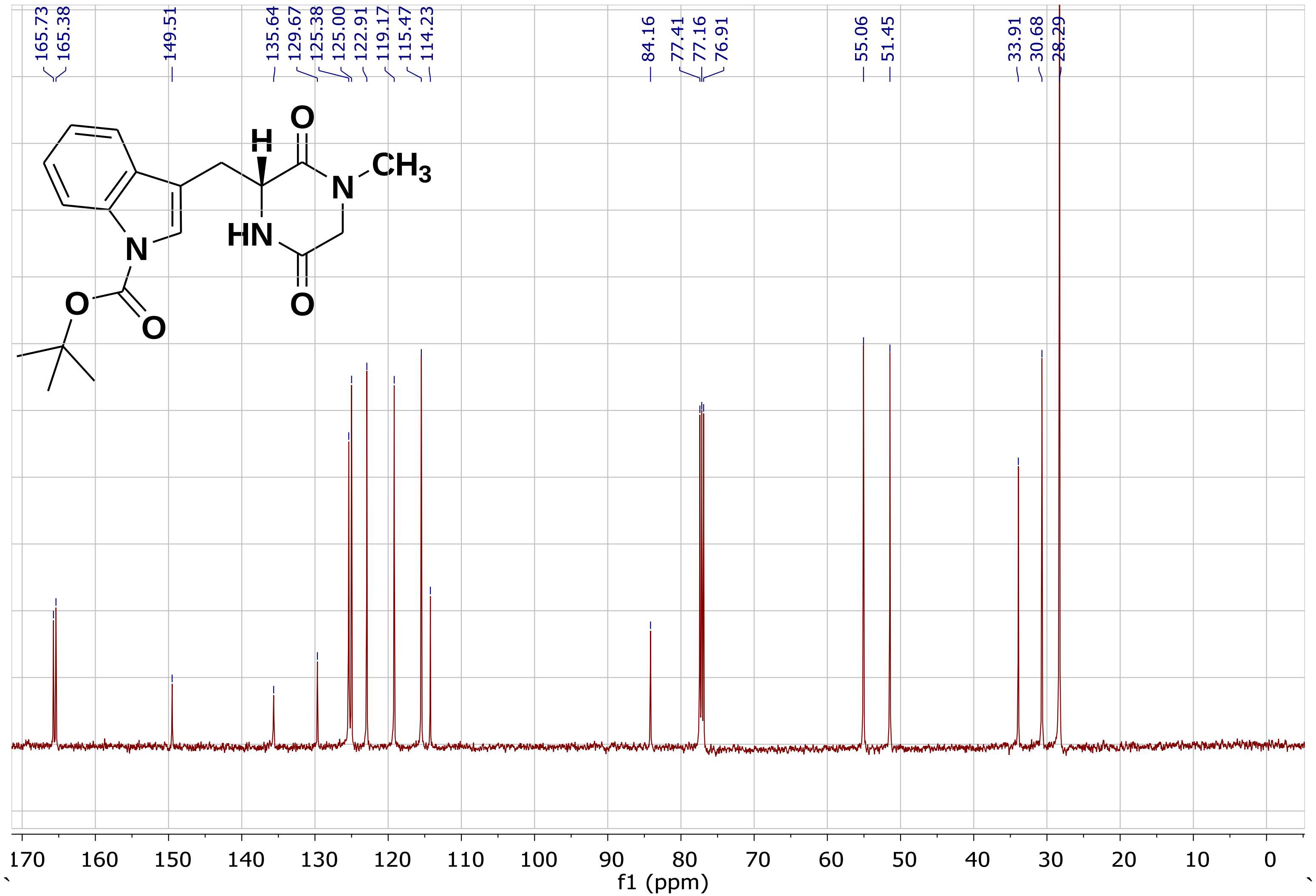

Figure S11. 13-NMR spectrum for **11a**.

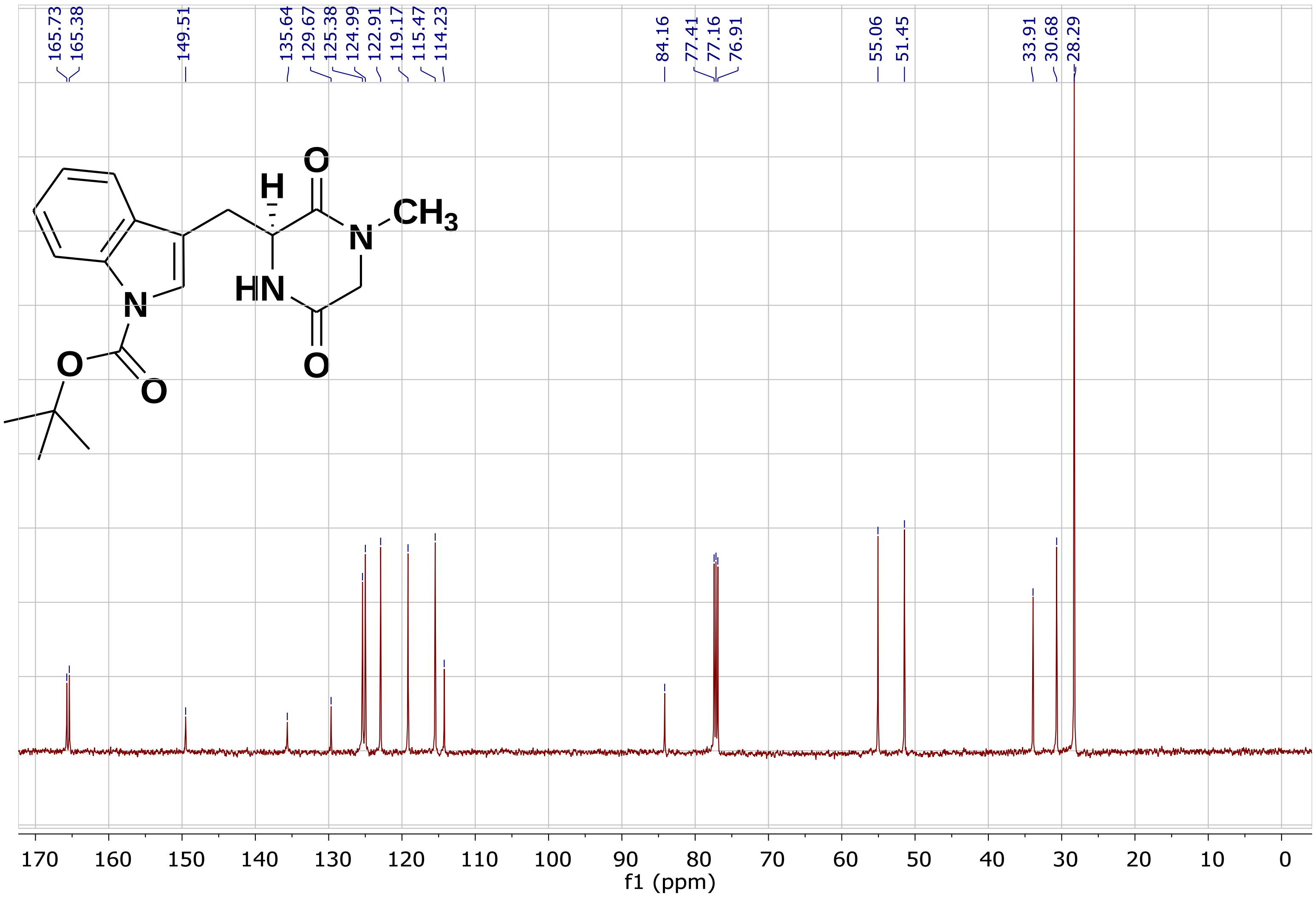

Figure S12. 13C-NMR spectrum for **11b**.

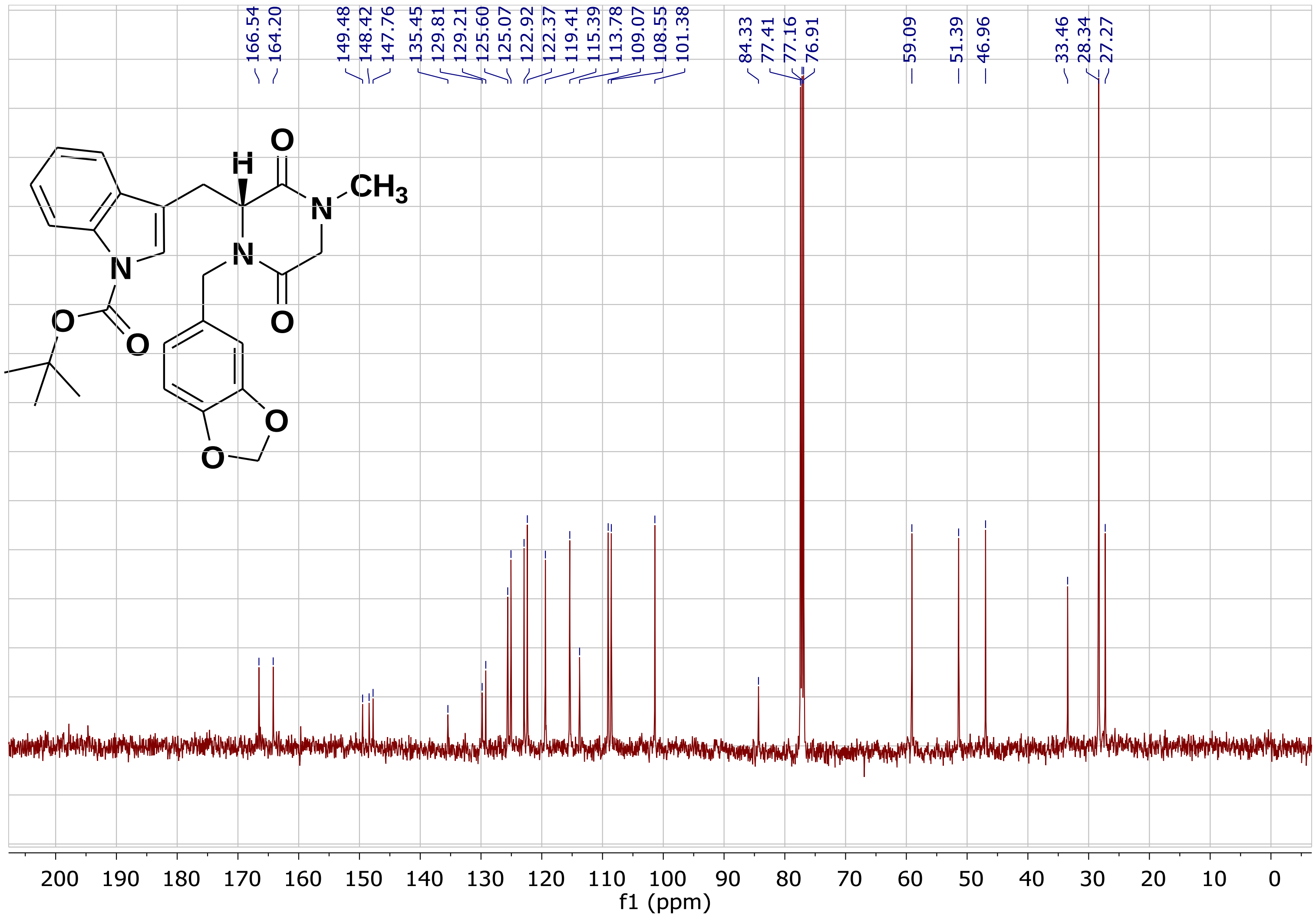

Figure S13. 13C-NMR spectrum for **12a**.

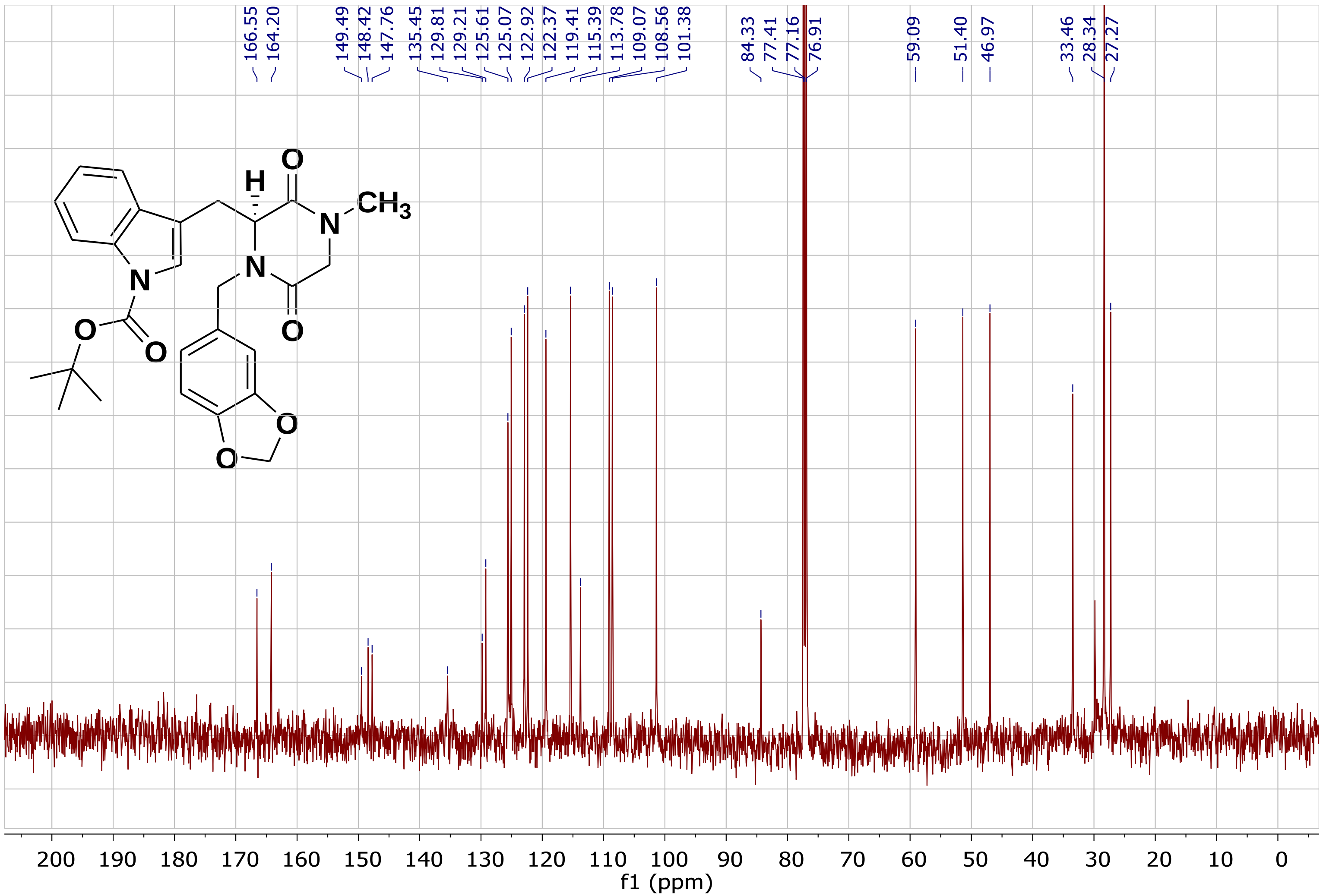

Figure S14. 13C-NMR spectrum for **12b**.

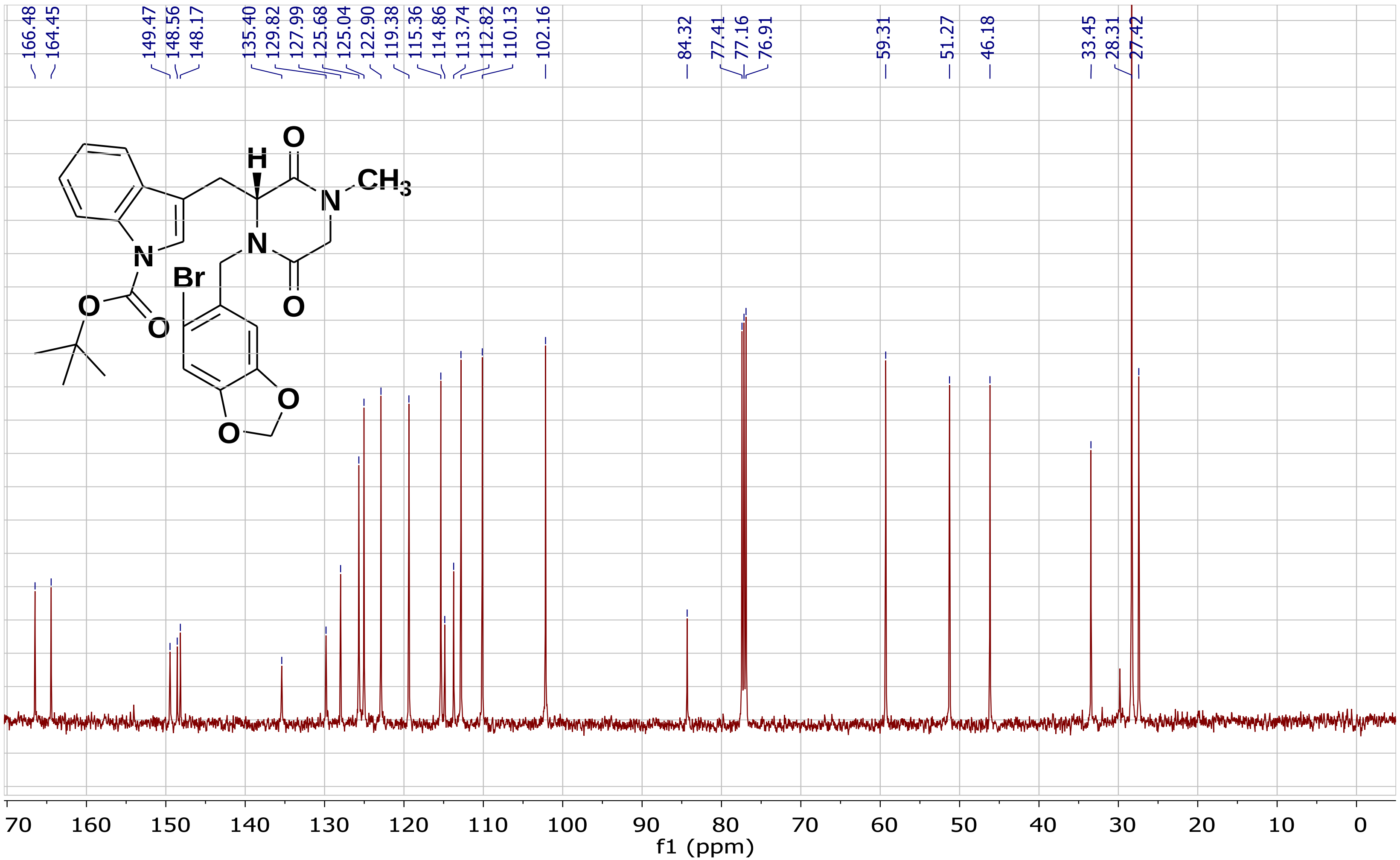

Figure S15. 13C-NMR spectrum for **13a**.

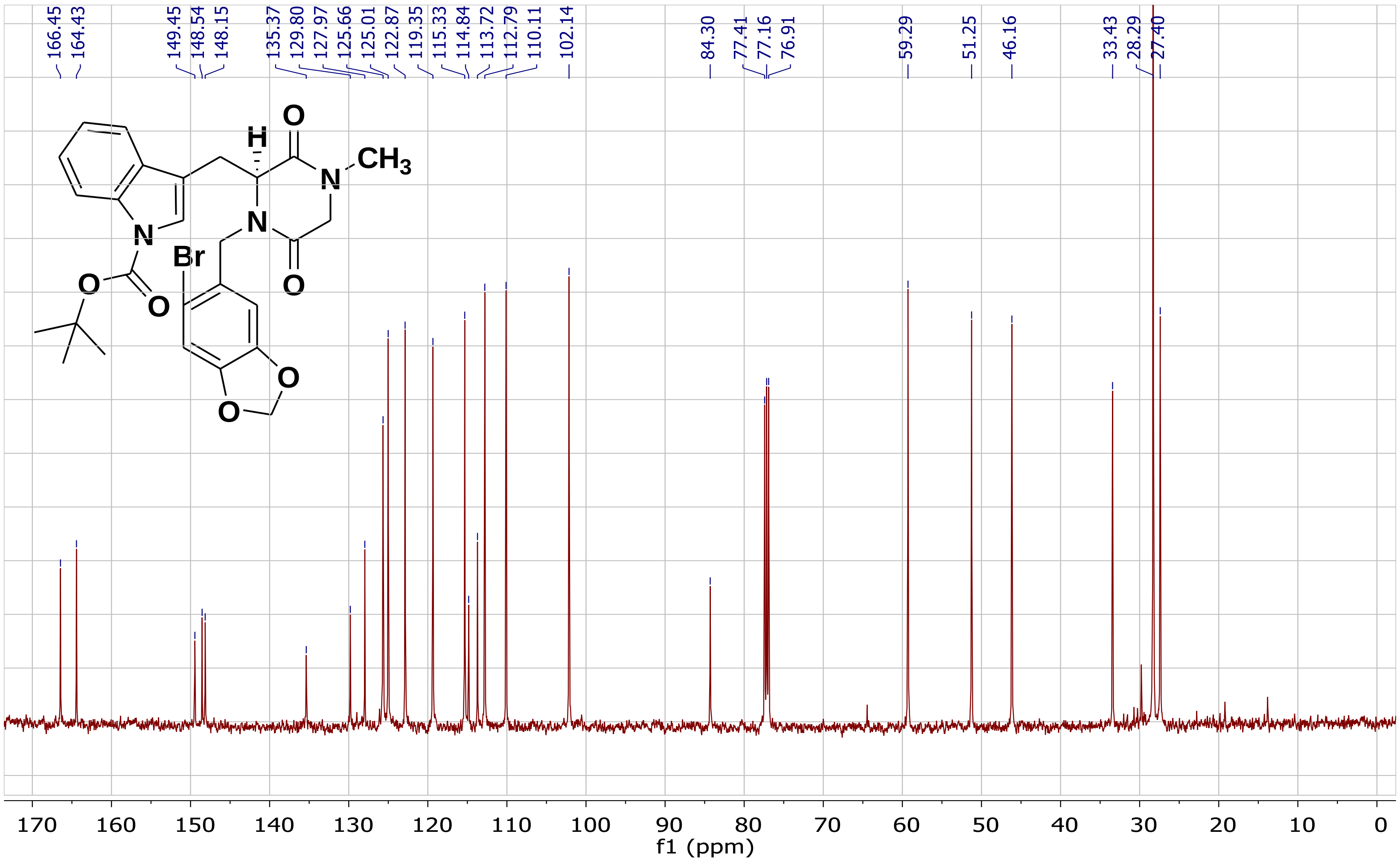

Figure S16. 13C-NMR spectrum for **13b**.

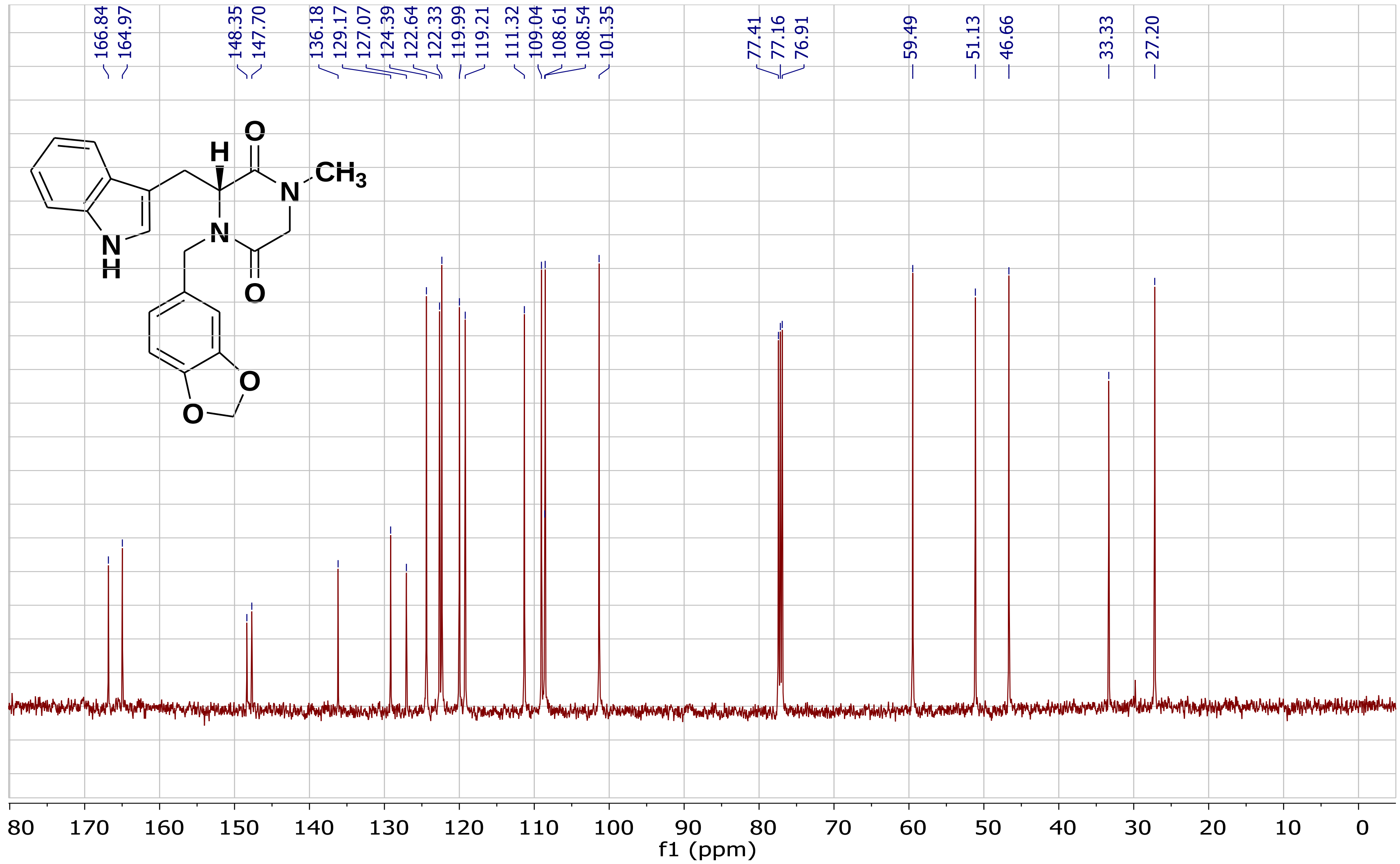

Figure S17. 13C-NMR spectrum for **6a**.

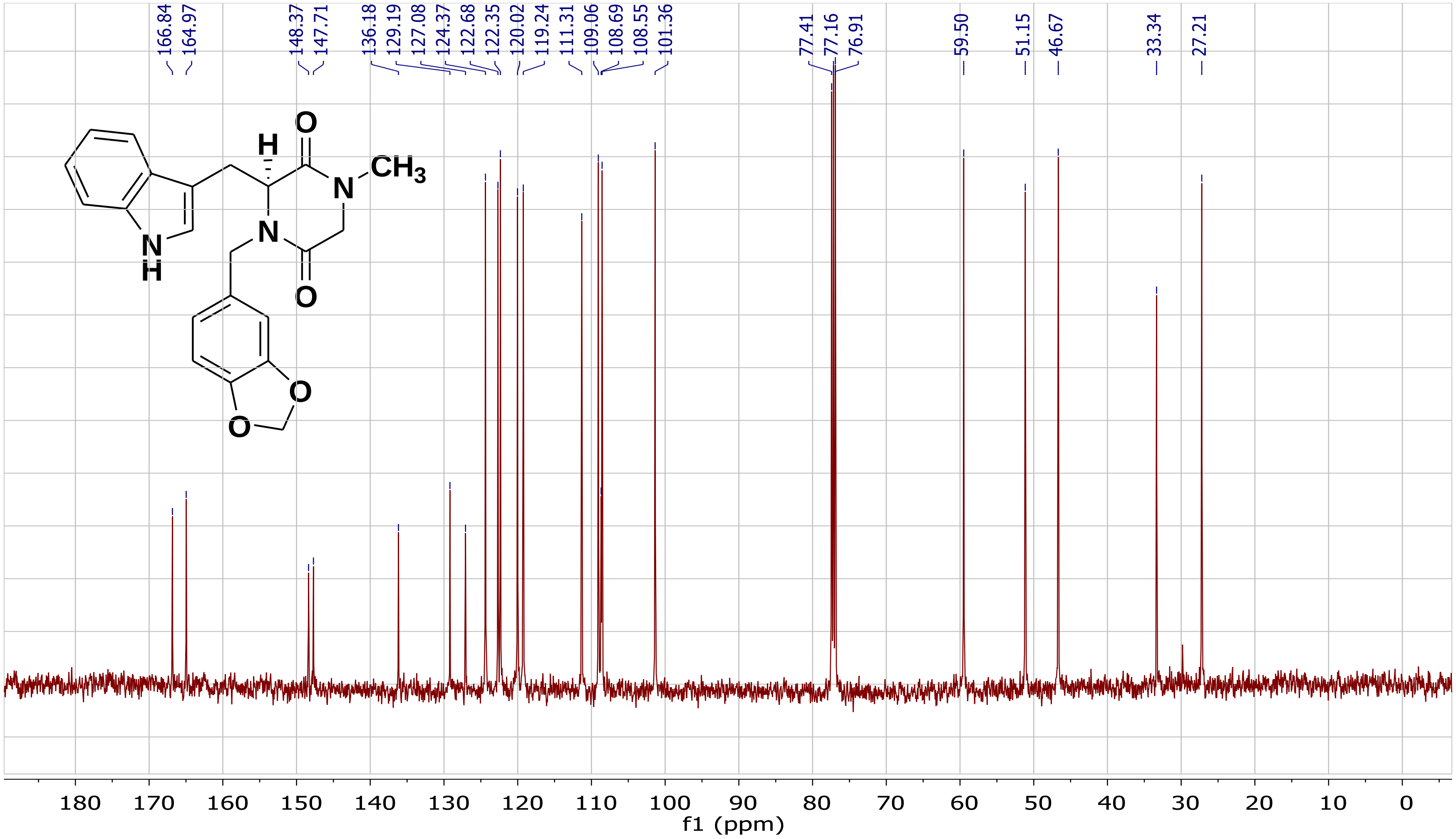

Figure S18. 13C-NMR spectrum for **6b.**

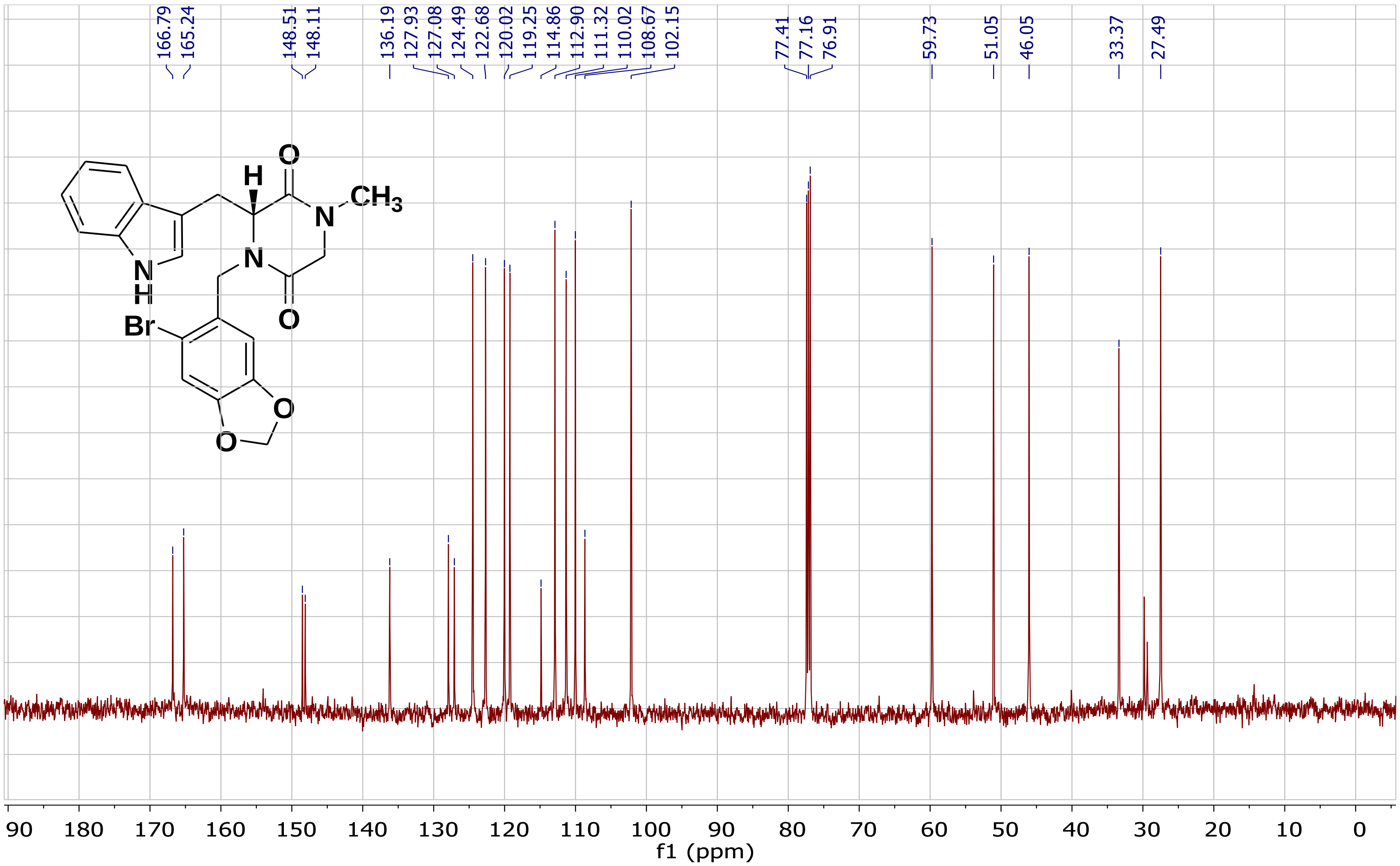

Figure S19. 13C-NMR spectrum for **7a**.

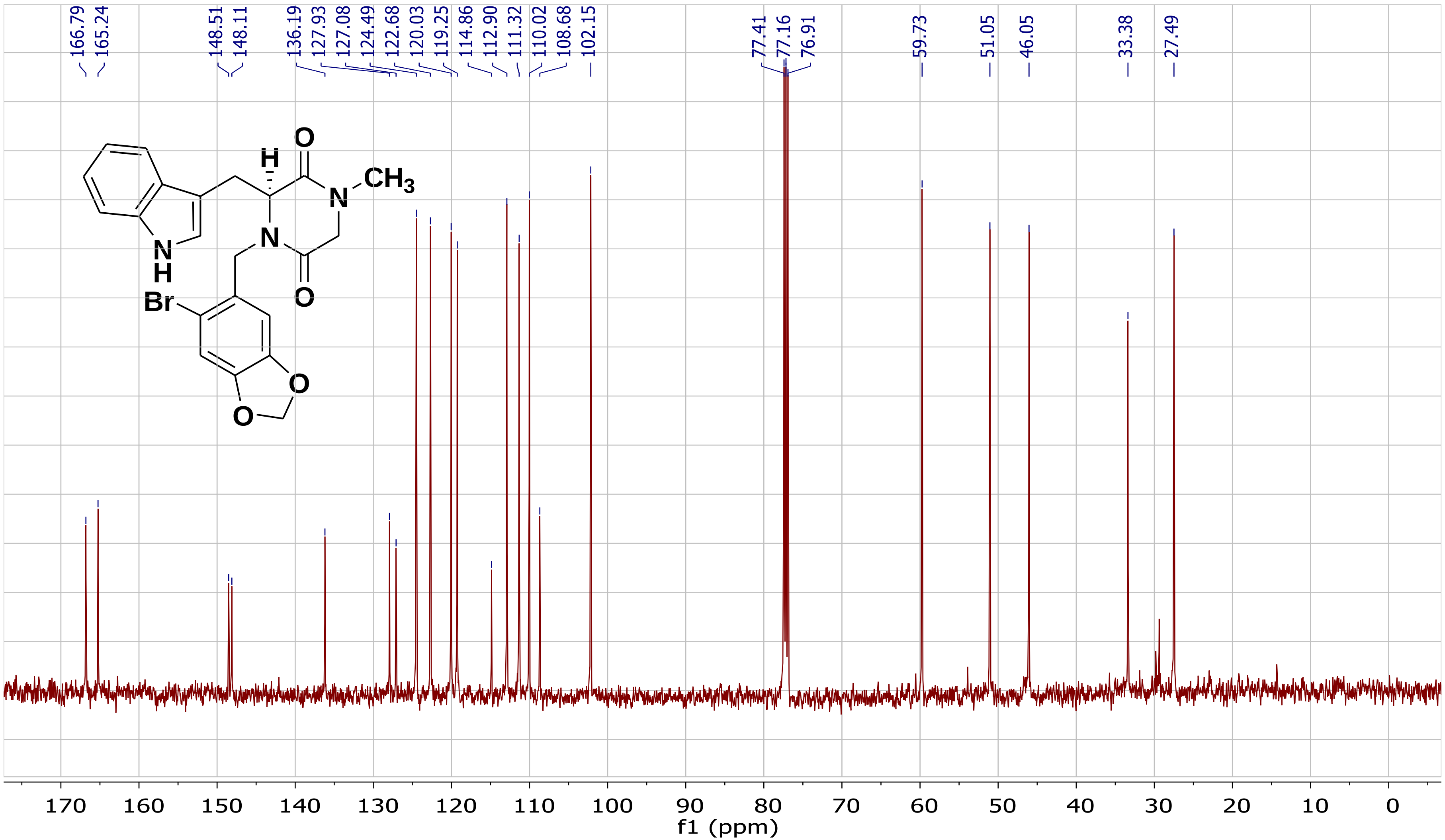

Figure S20. 13C-NMR spectrum for **7b**.

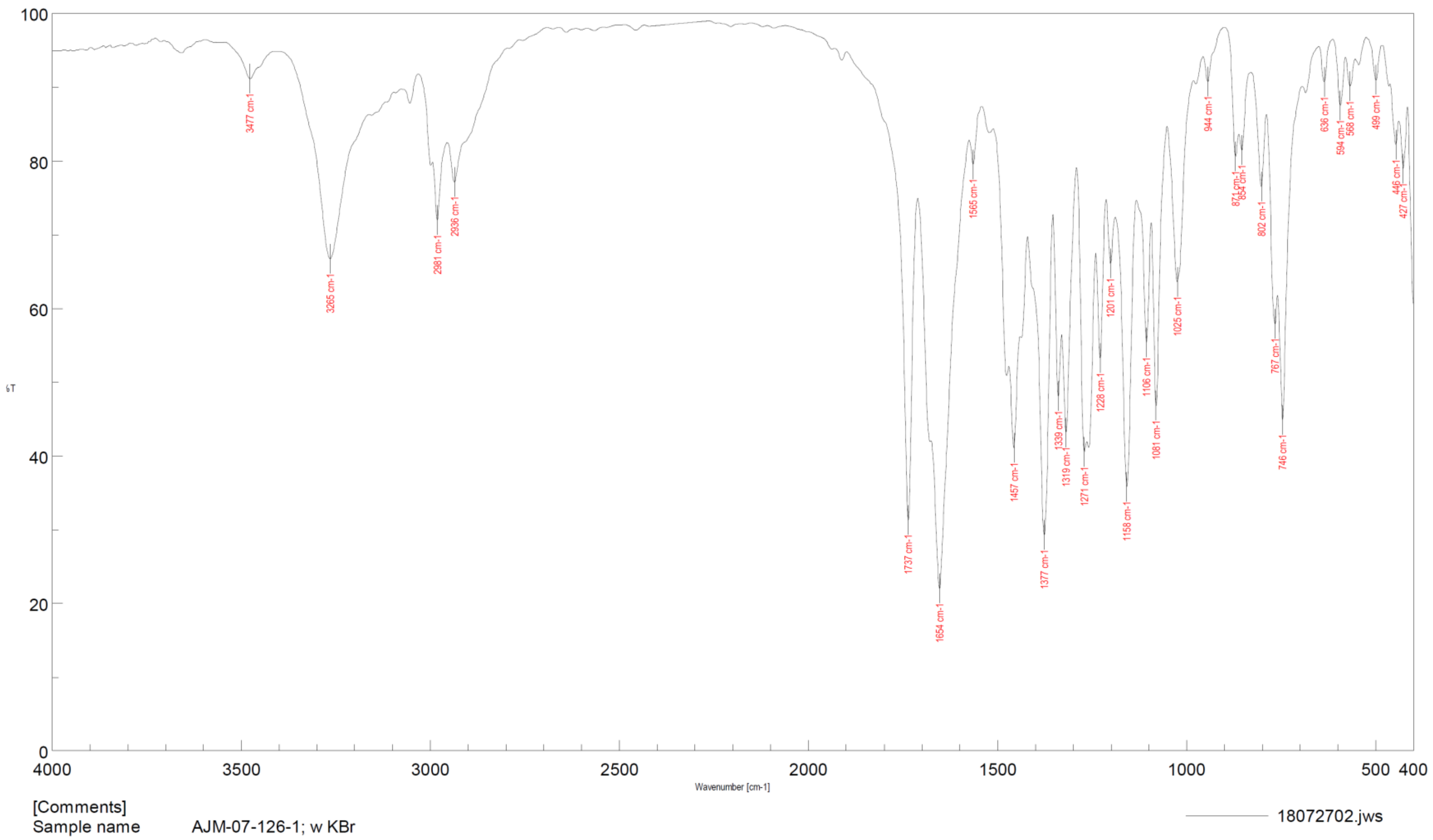

Figure S21. IR spectrum for **11a**.

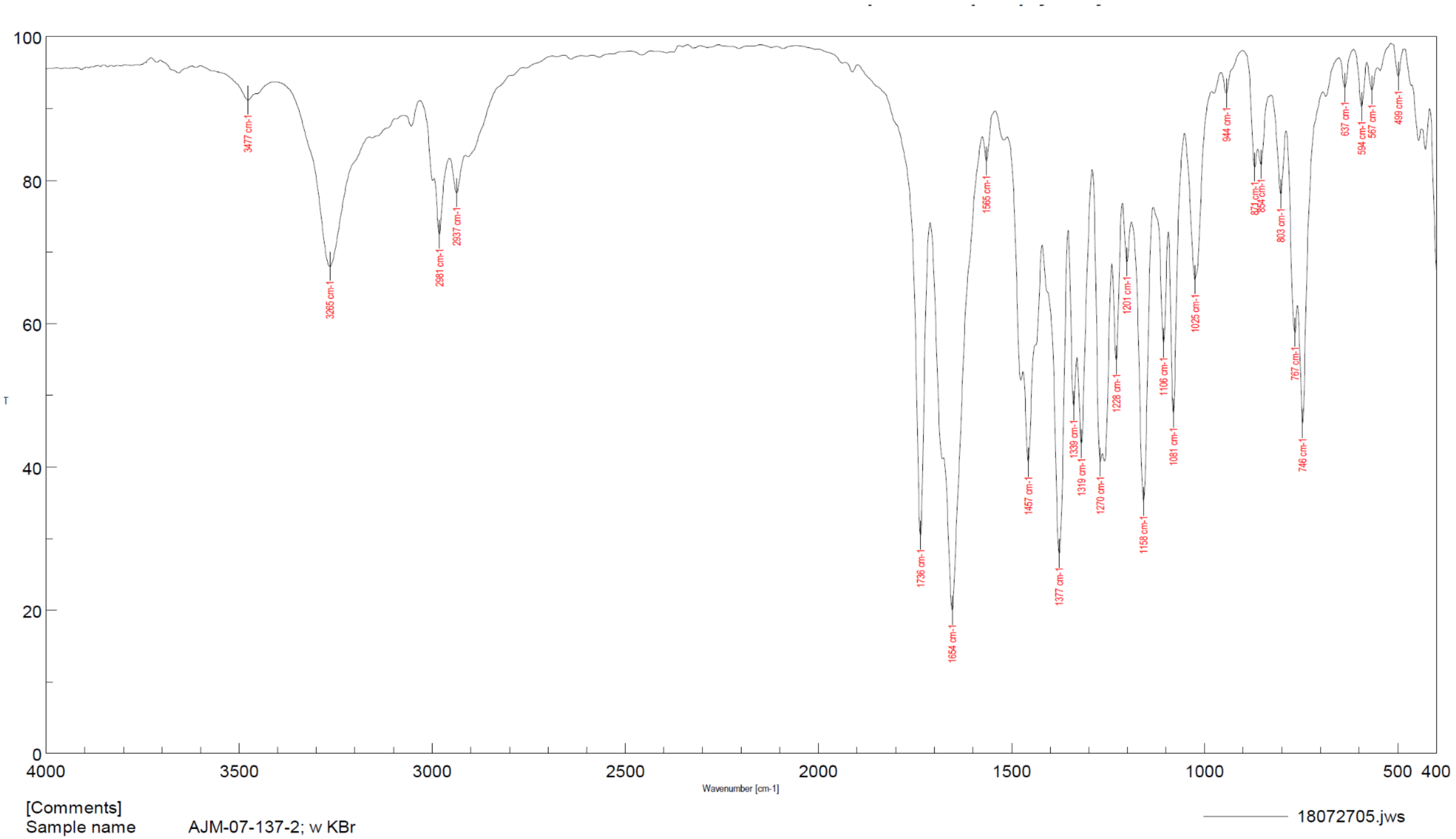

Figure S22. IR spectrum for **11b**.

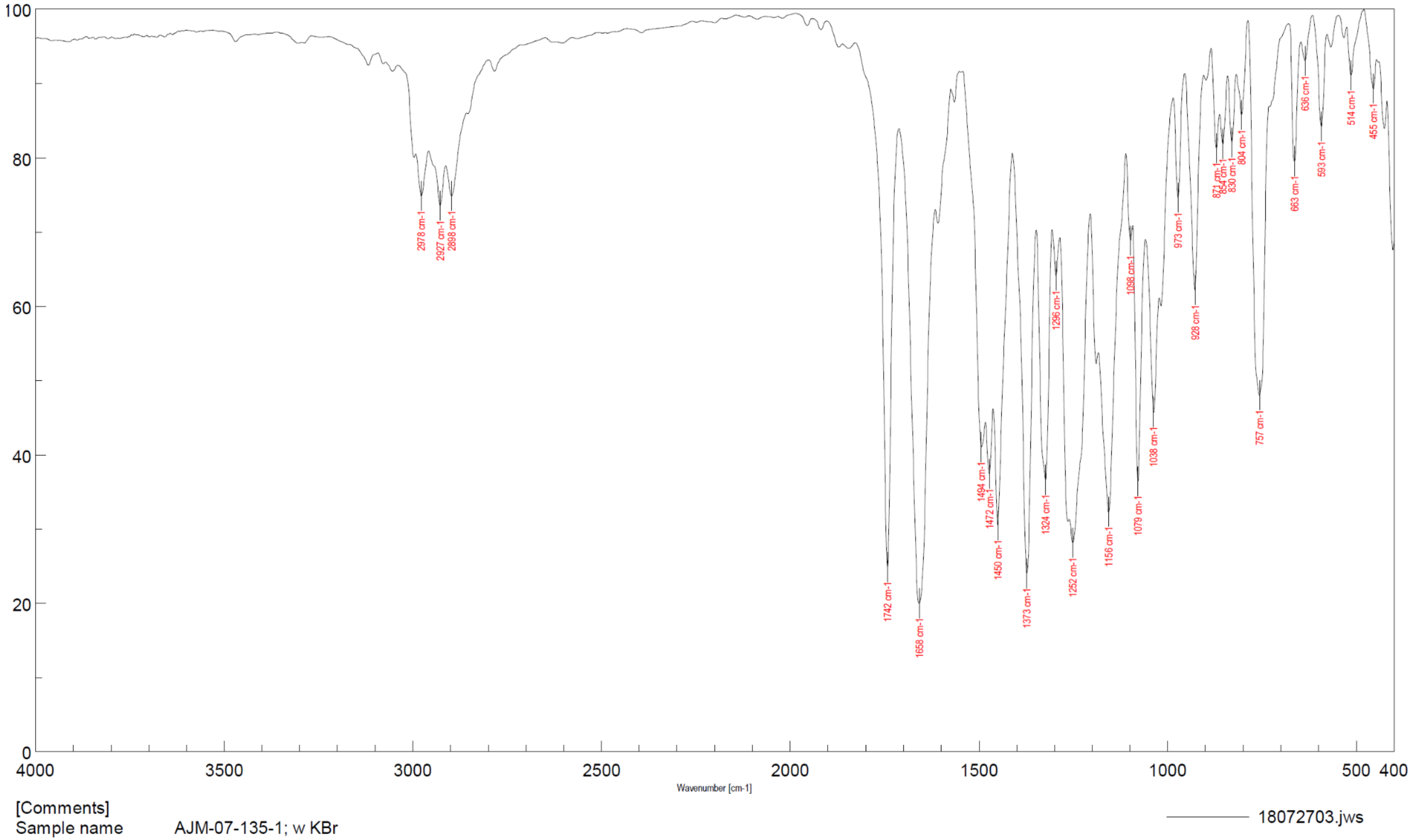

Figure S23. IR spectrum for **12a**.

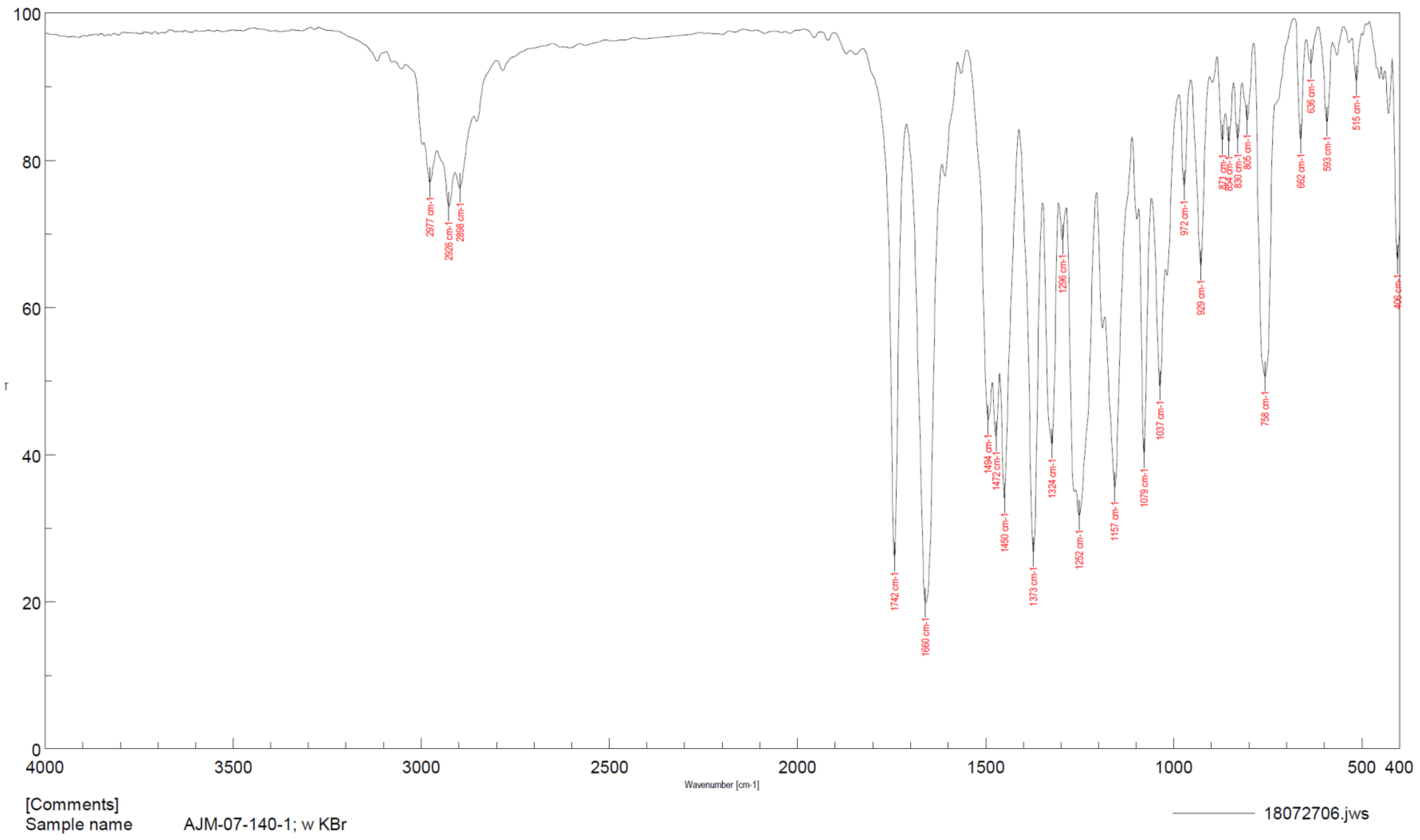

Figure S24. IR spectrum for **12b**.

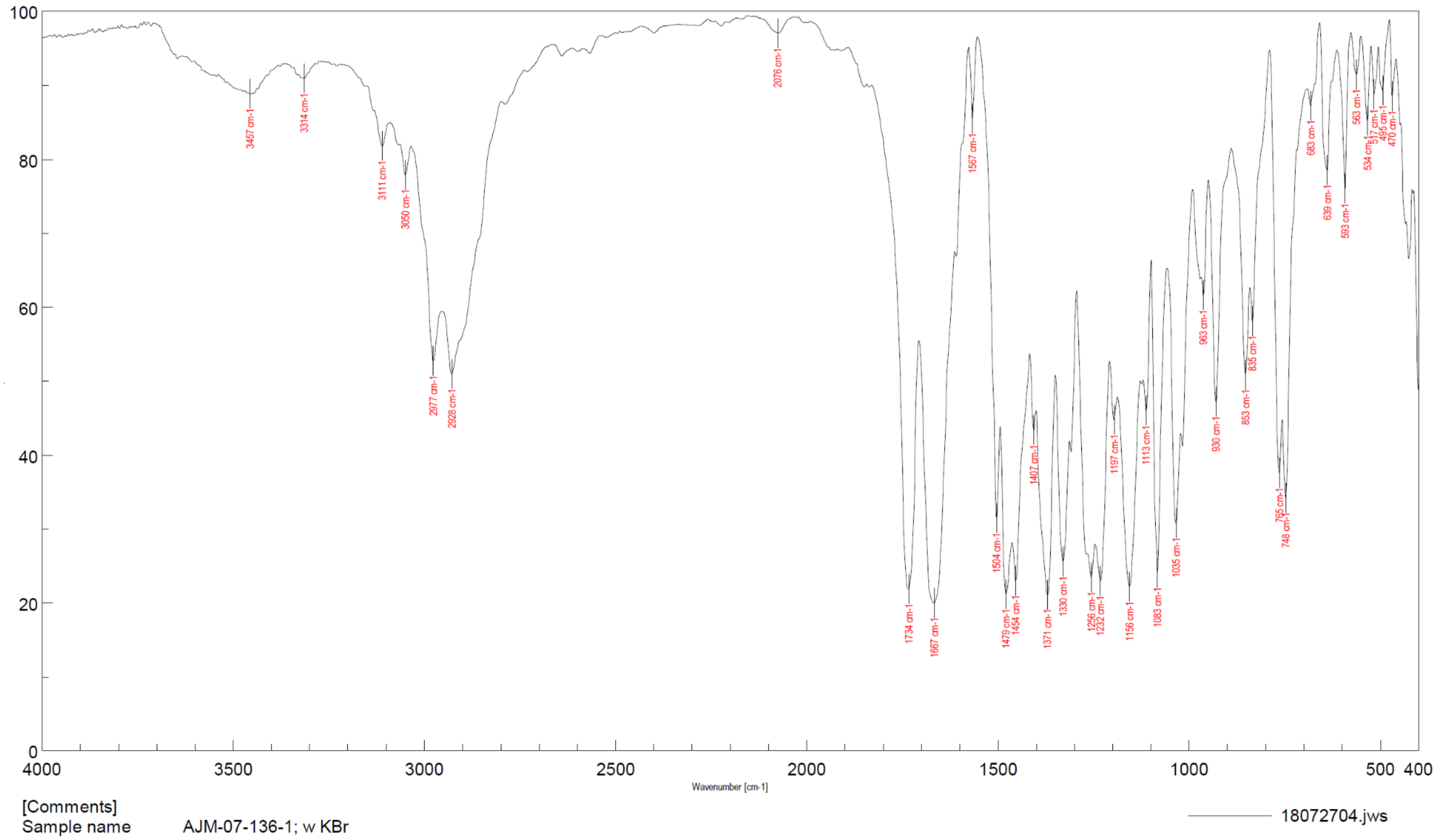

Figure S25. IR spectrum for **13a**.

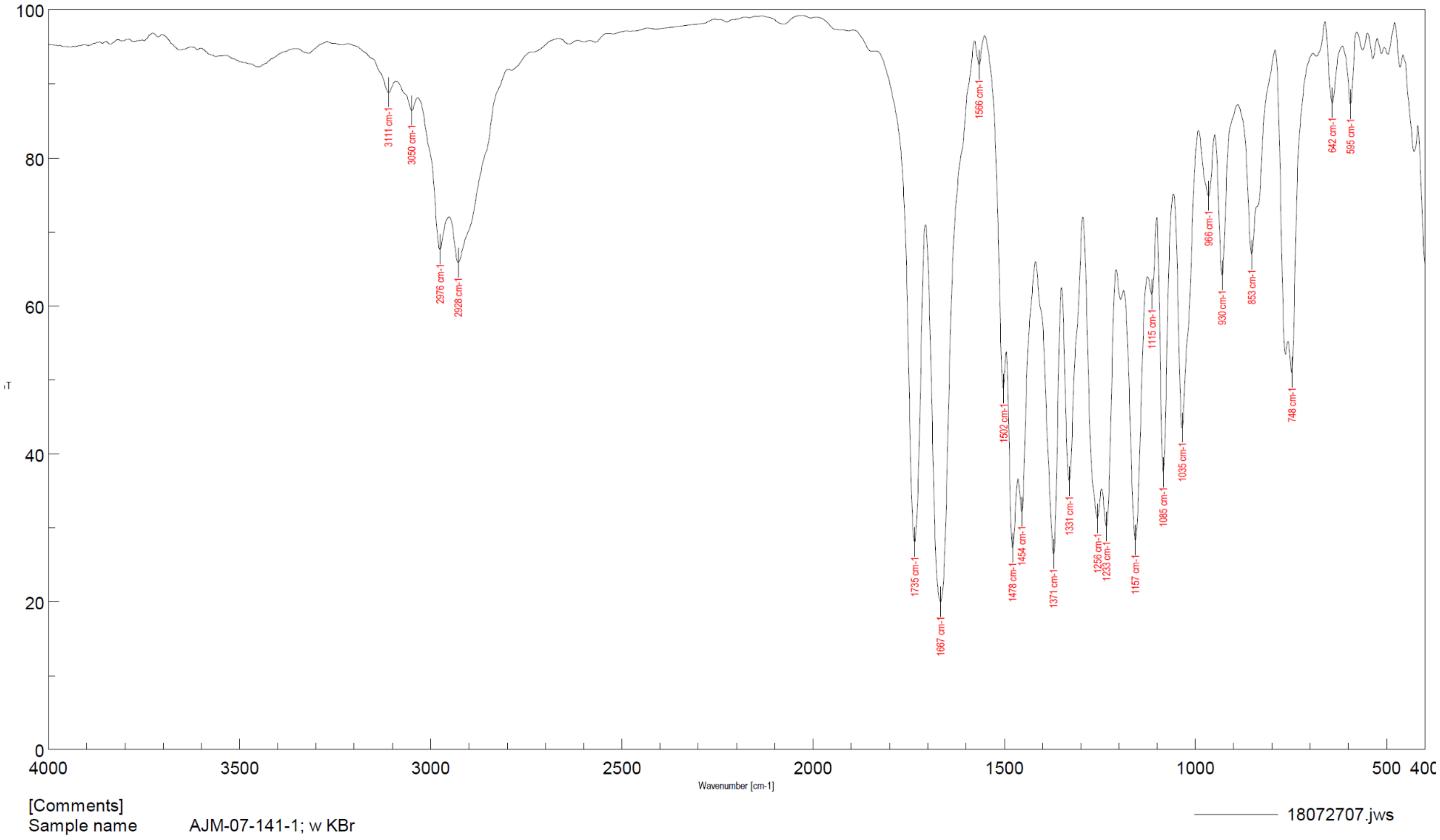

Figure S26. IR spectrum for **13b**.

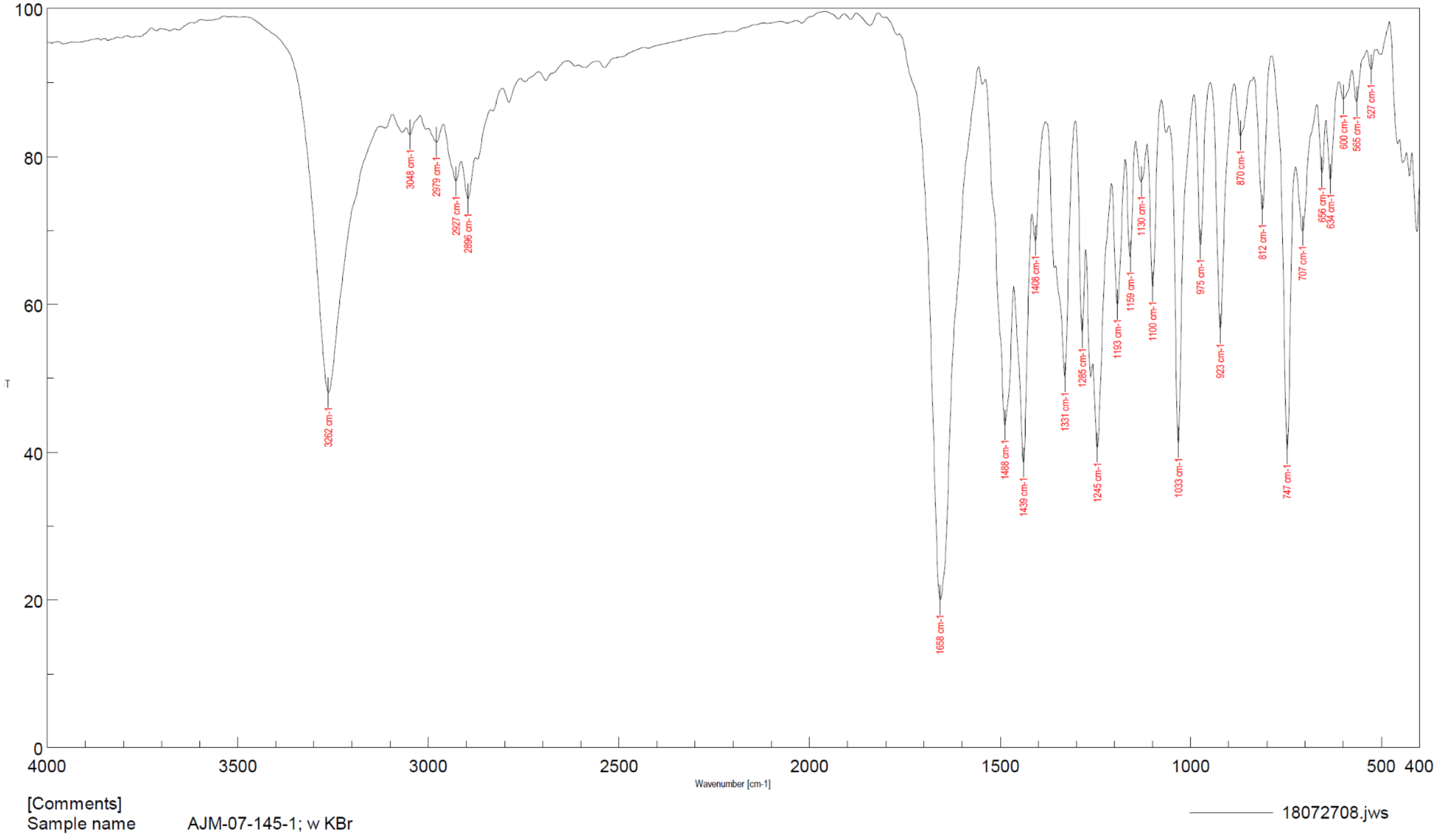

Figure S27. IR spectrum for **6a**.

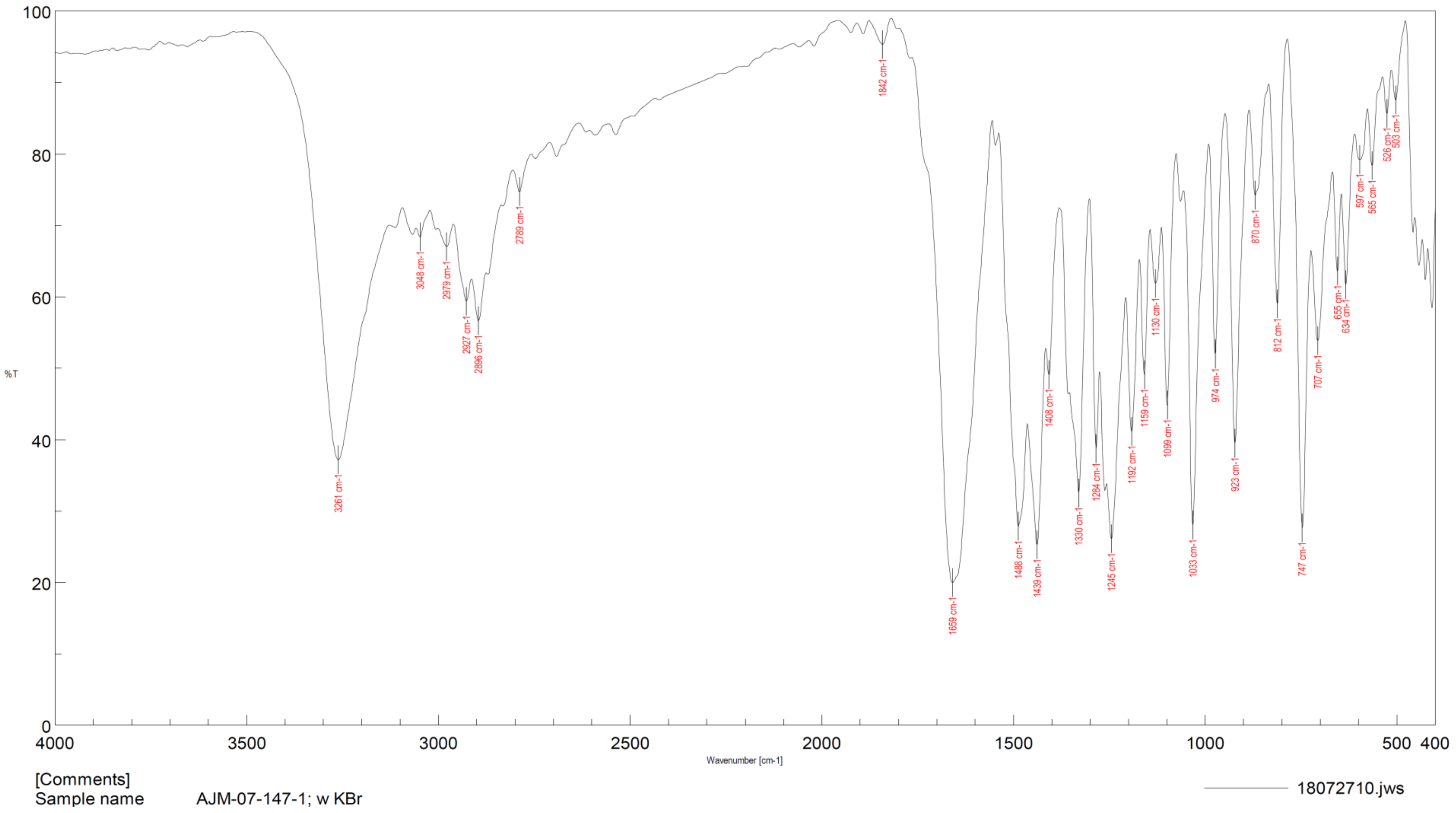

Figure S28. IR spectrum for **6b**.

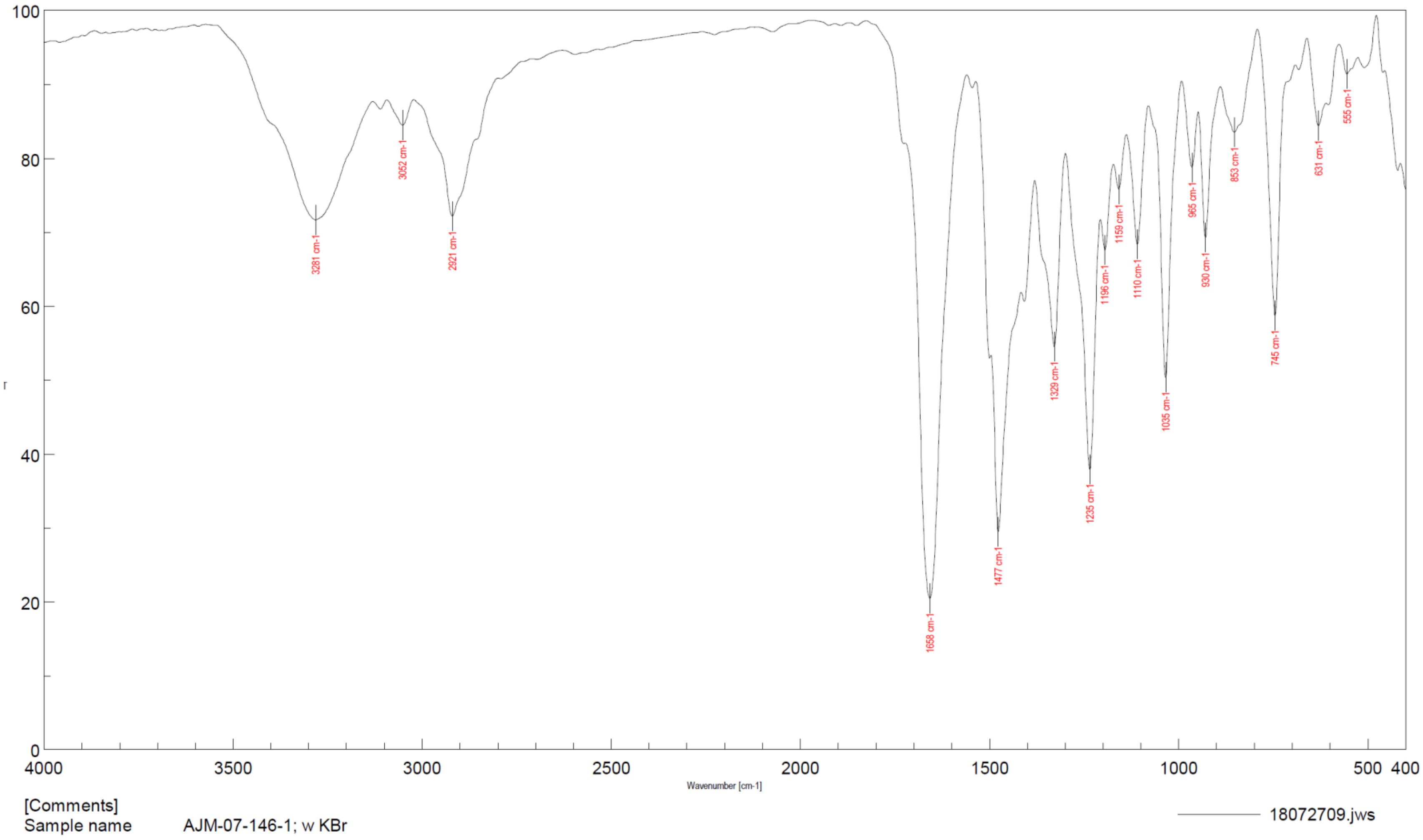

Figure S29. IR spectrum for **7a**.

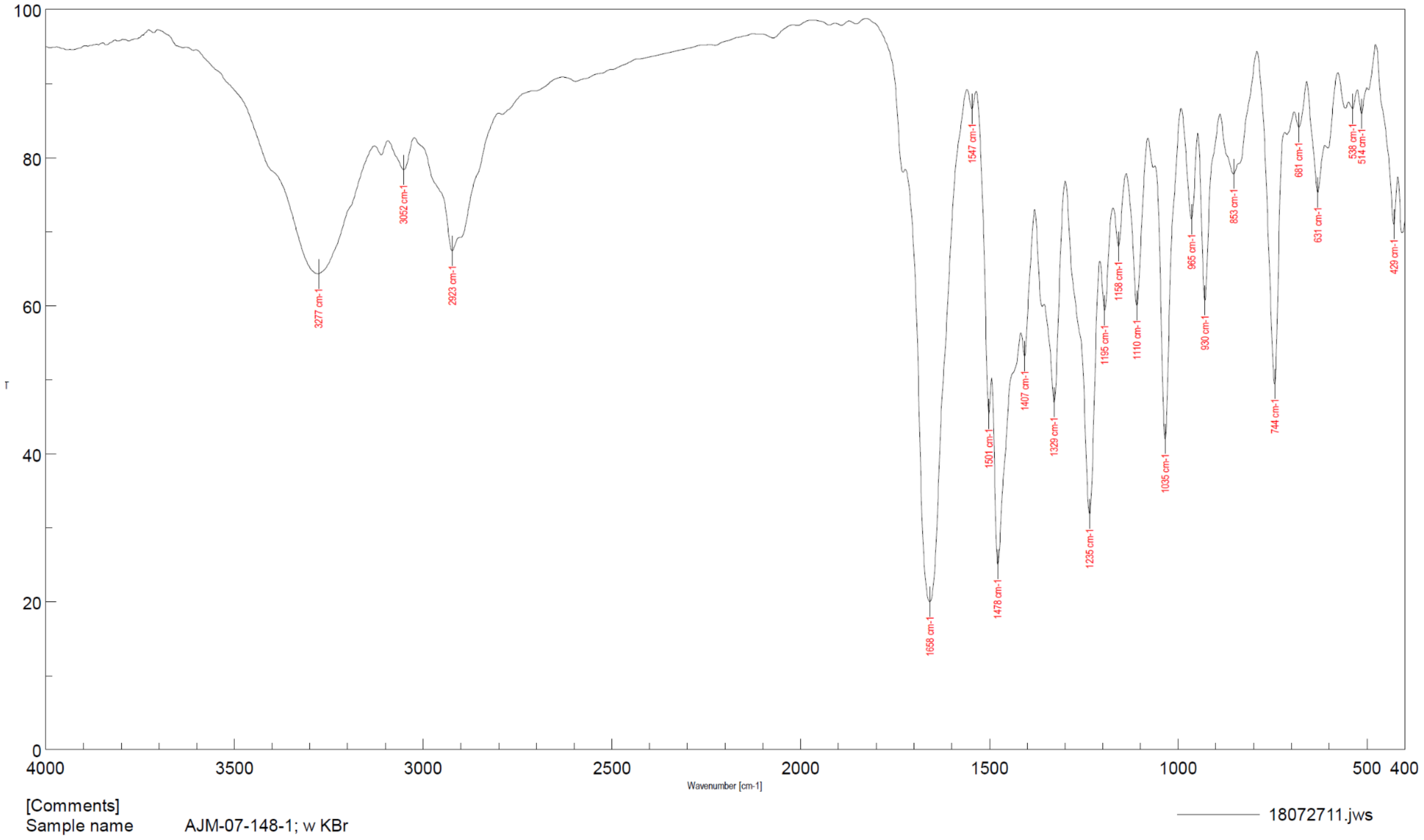

Figure S30. IR spectrum for **7b**.

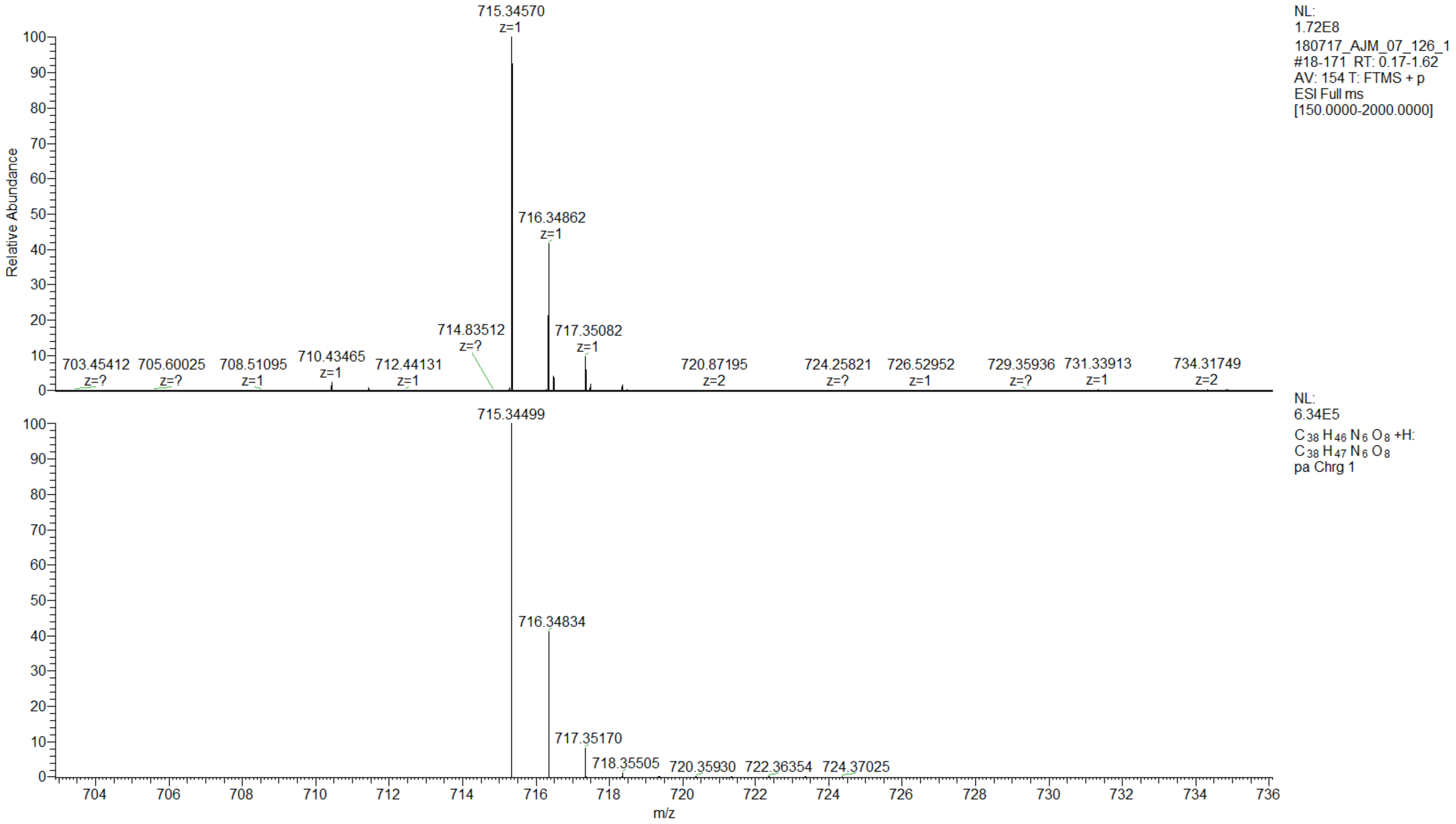

Figure S31. HRMS spectrum for **11a**.

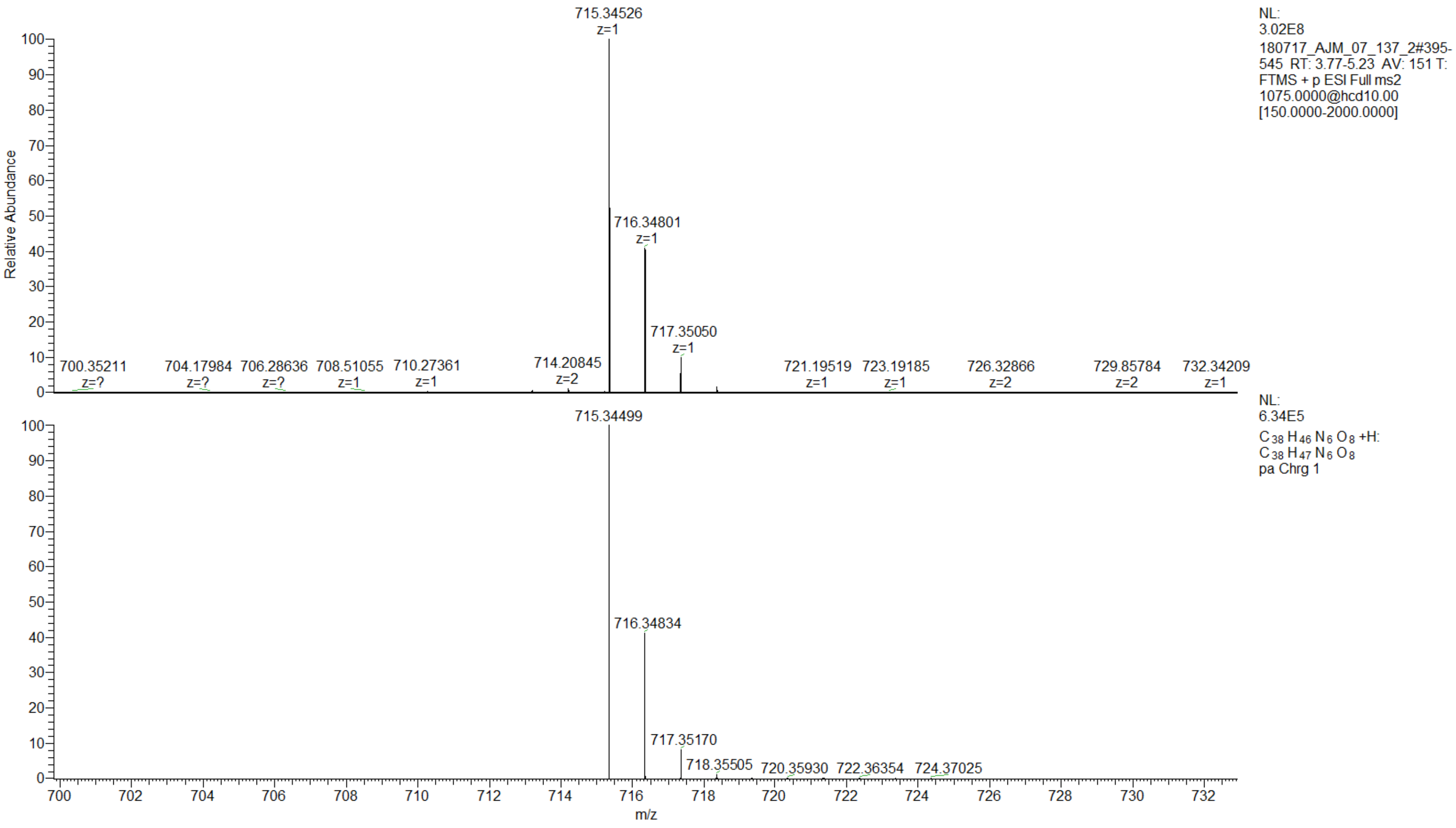

Figure S32. HRMS spectrum for **11b**.

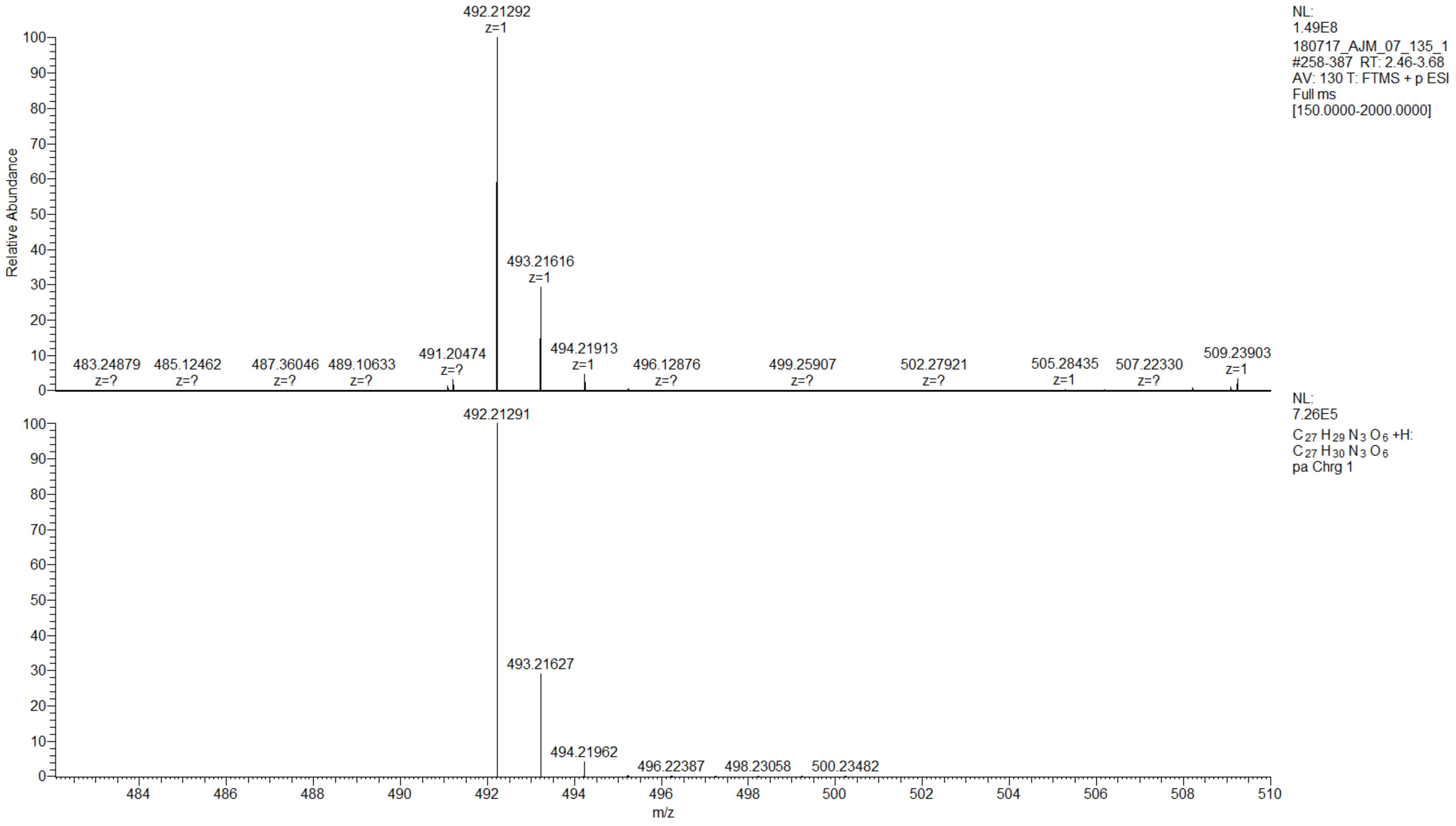

Figure S33. HRMS spectrum for **12a**.

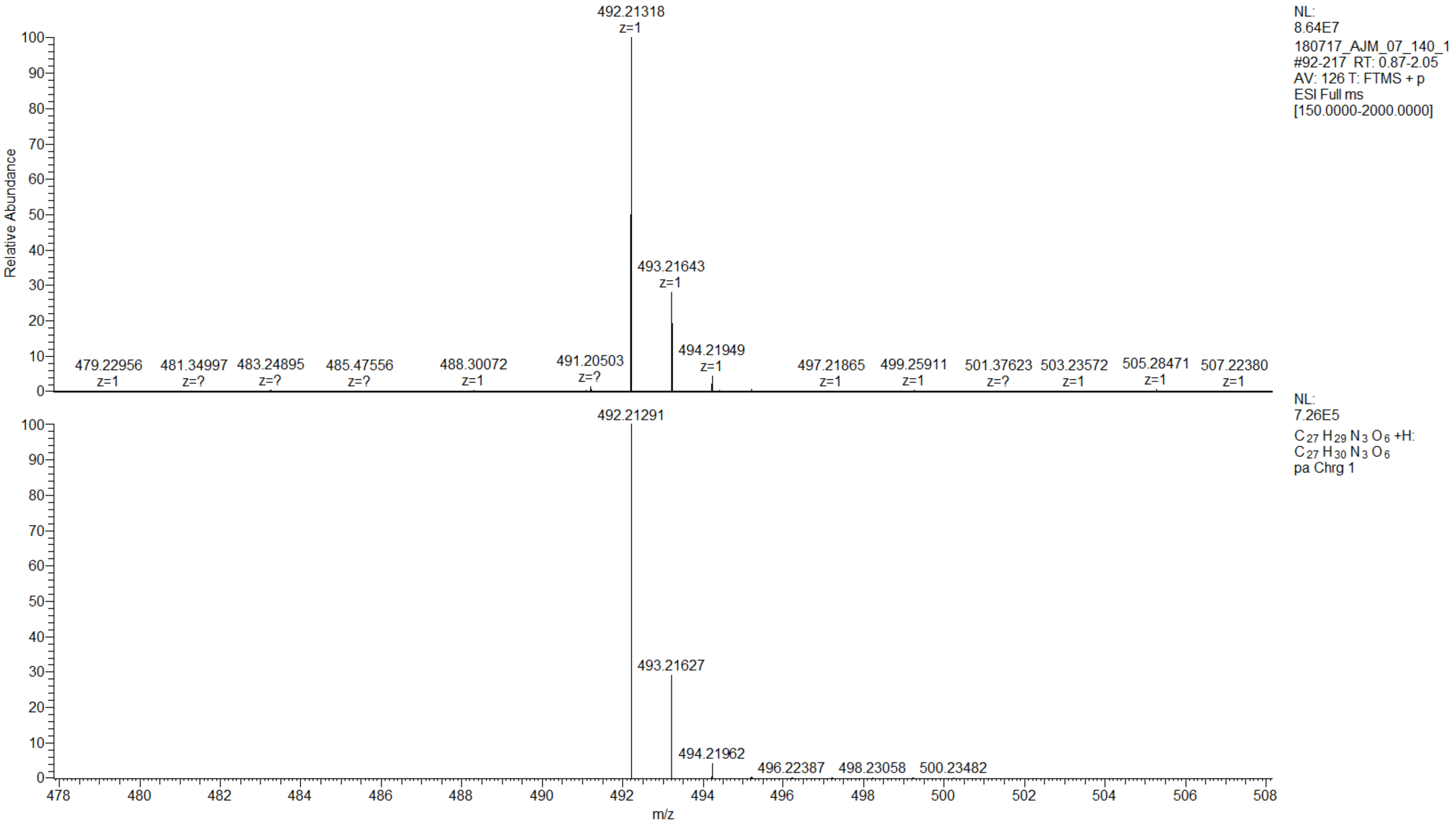

Figure S34. HRMS spectrum for **12b**.

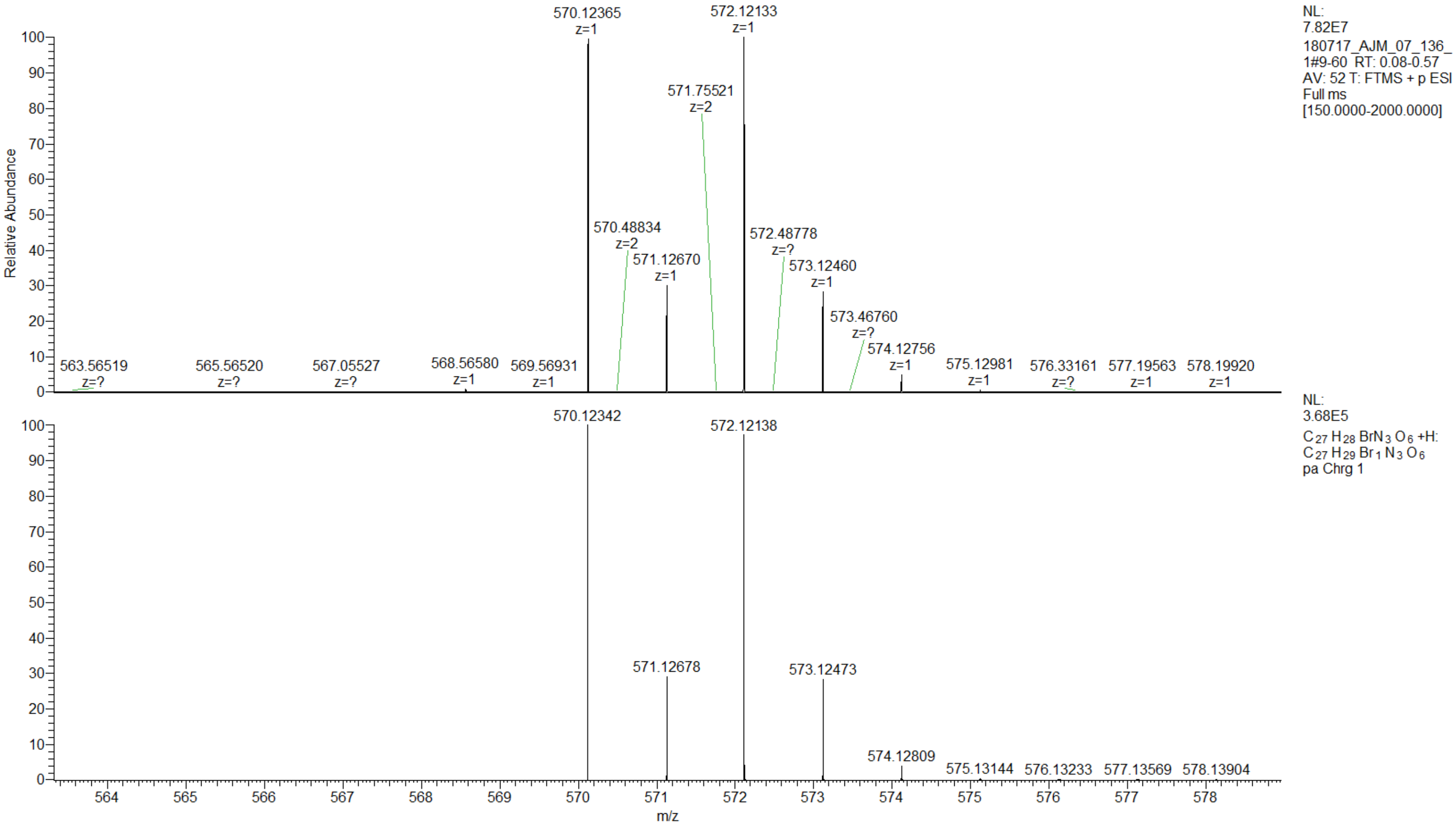

Figure S35. HRMS spectrum for **13a**.

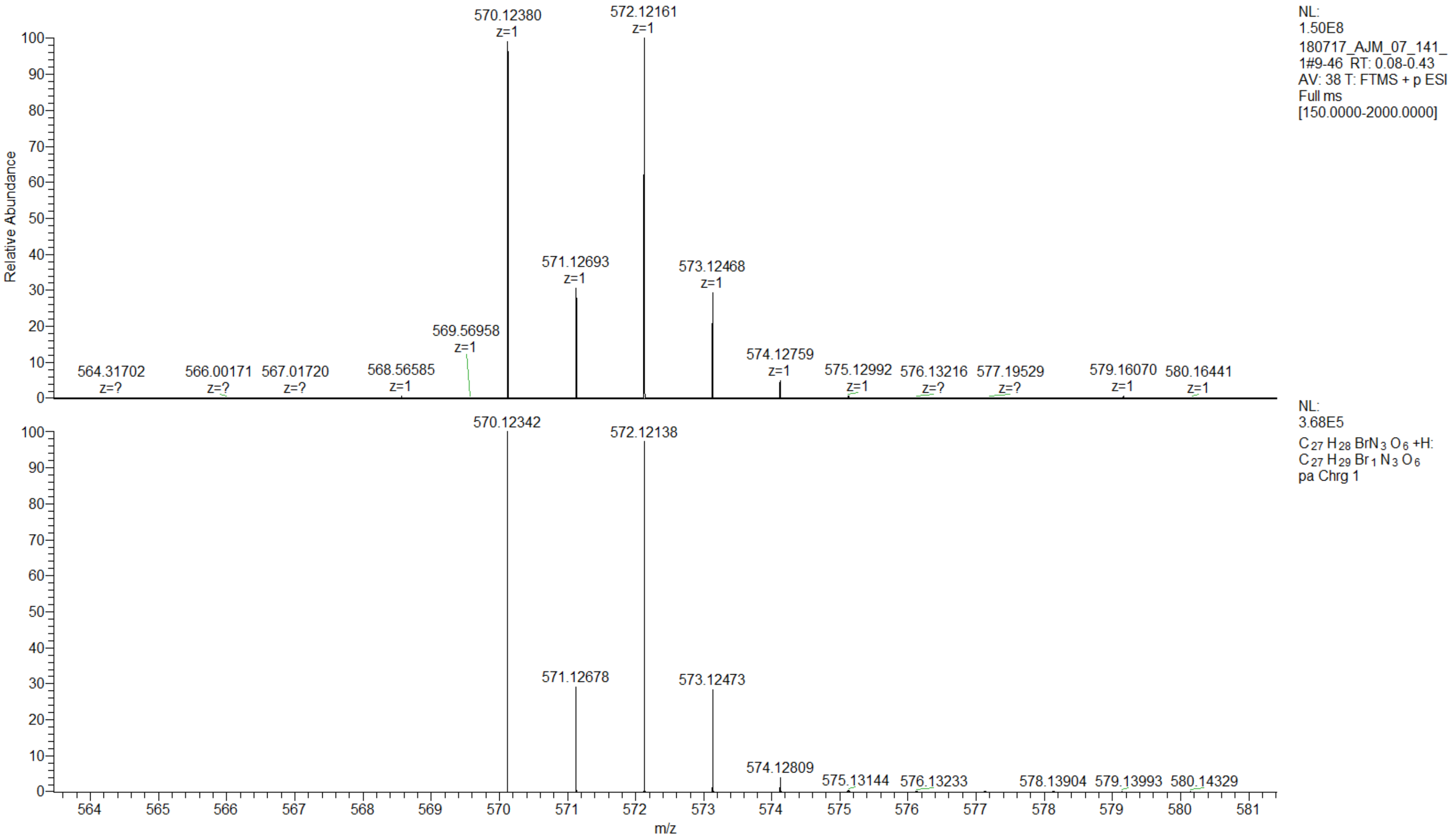

Figure S36. HRMS spectrum for **13b**.

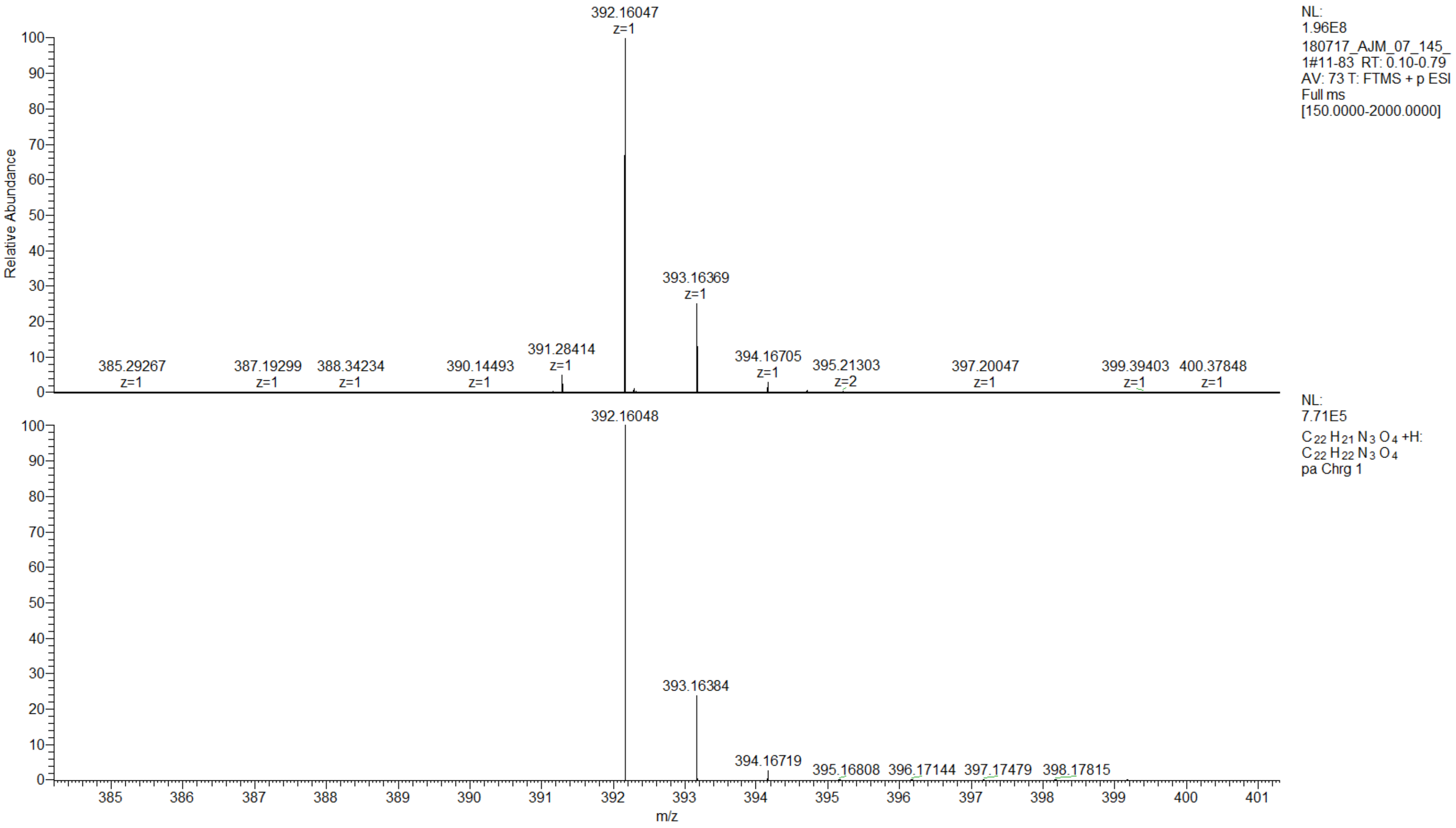

Figure S37. HRMS spectrum for **6a.**

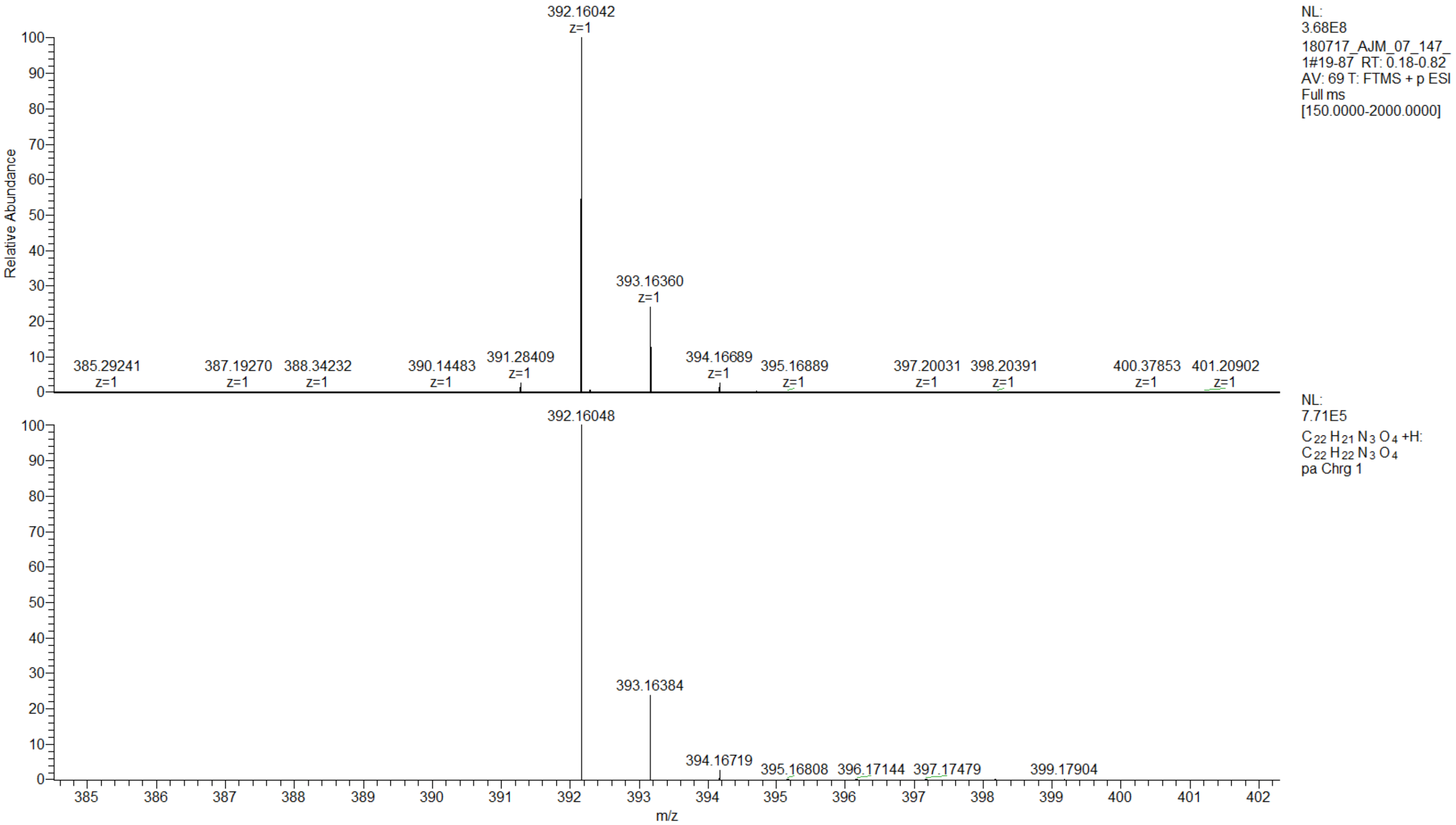

Figure S38. HRMS spectrum for **6b**.

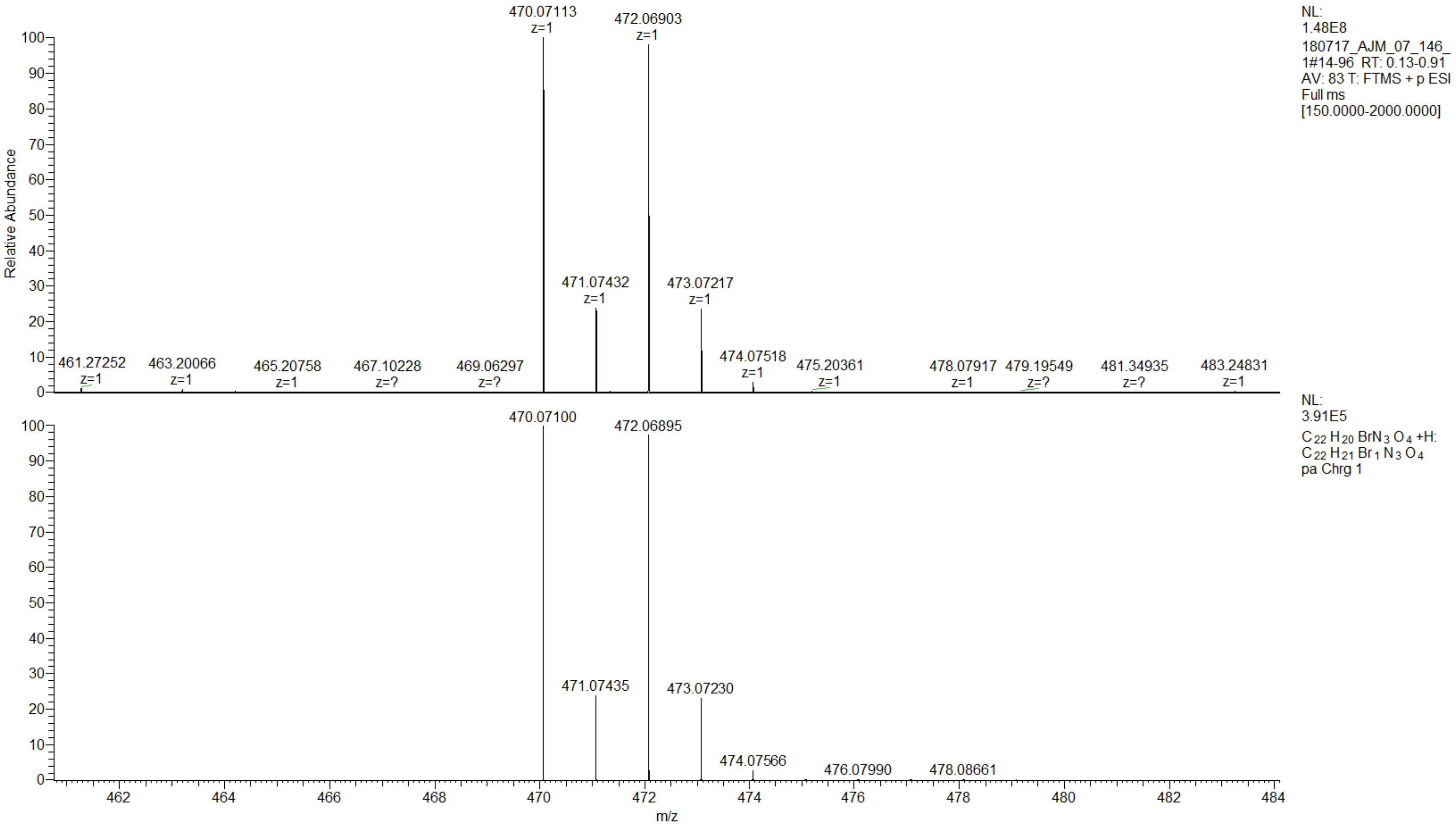

Figure S39. HRMS spectrum for **7a**.

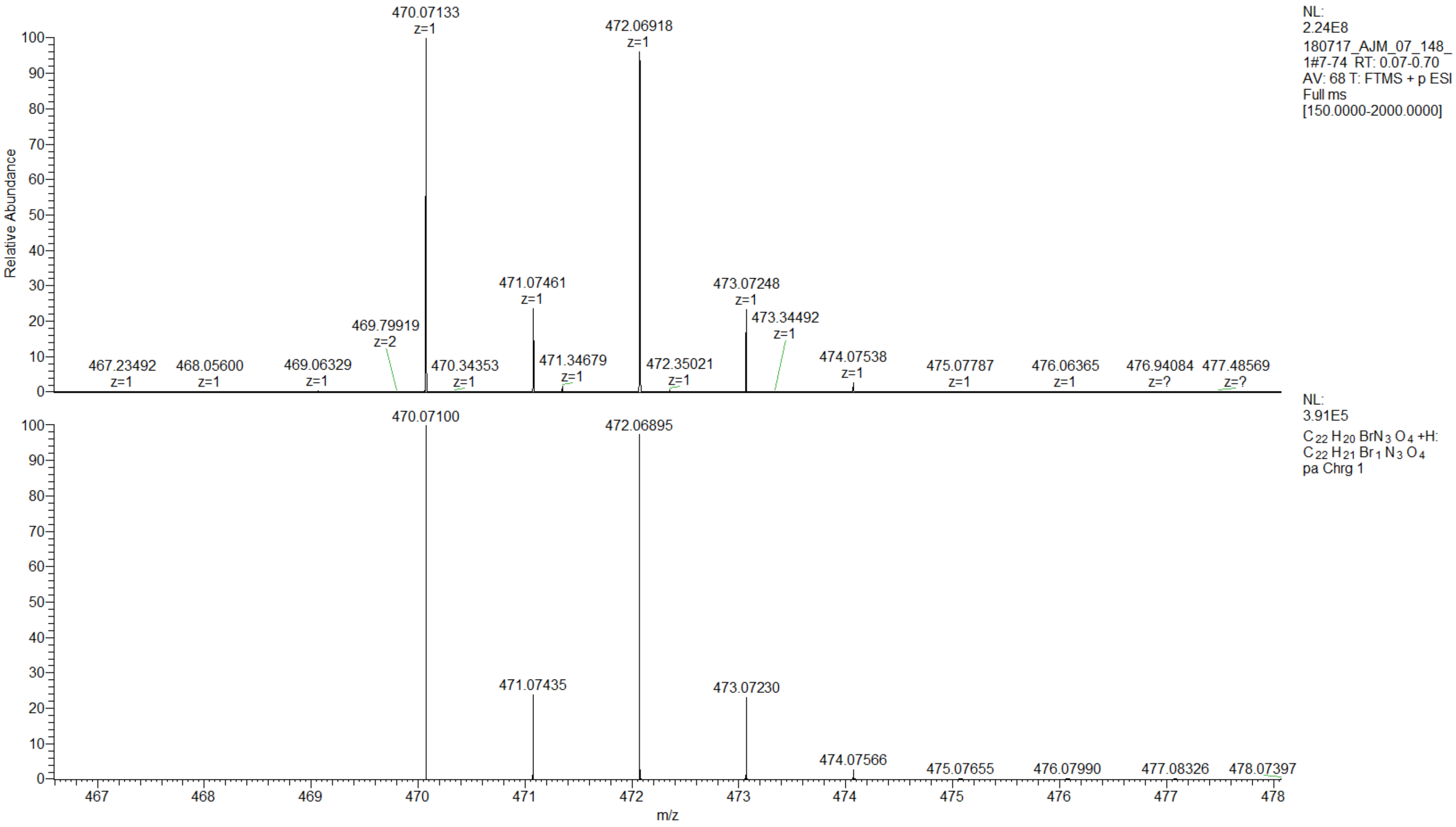

Figure S40. HRMS spectrum for **7b**.

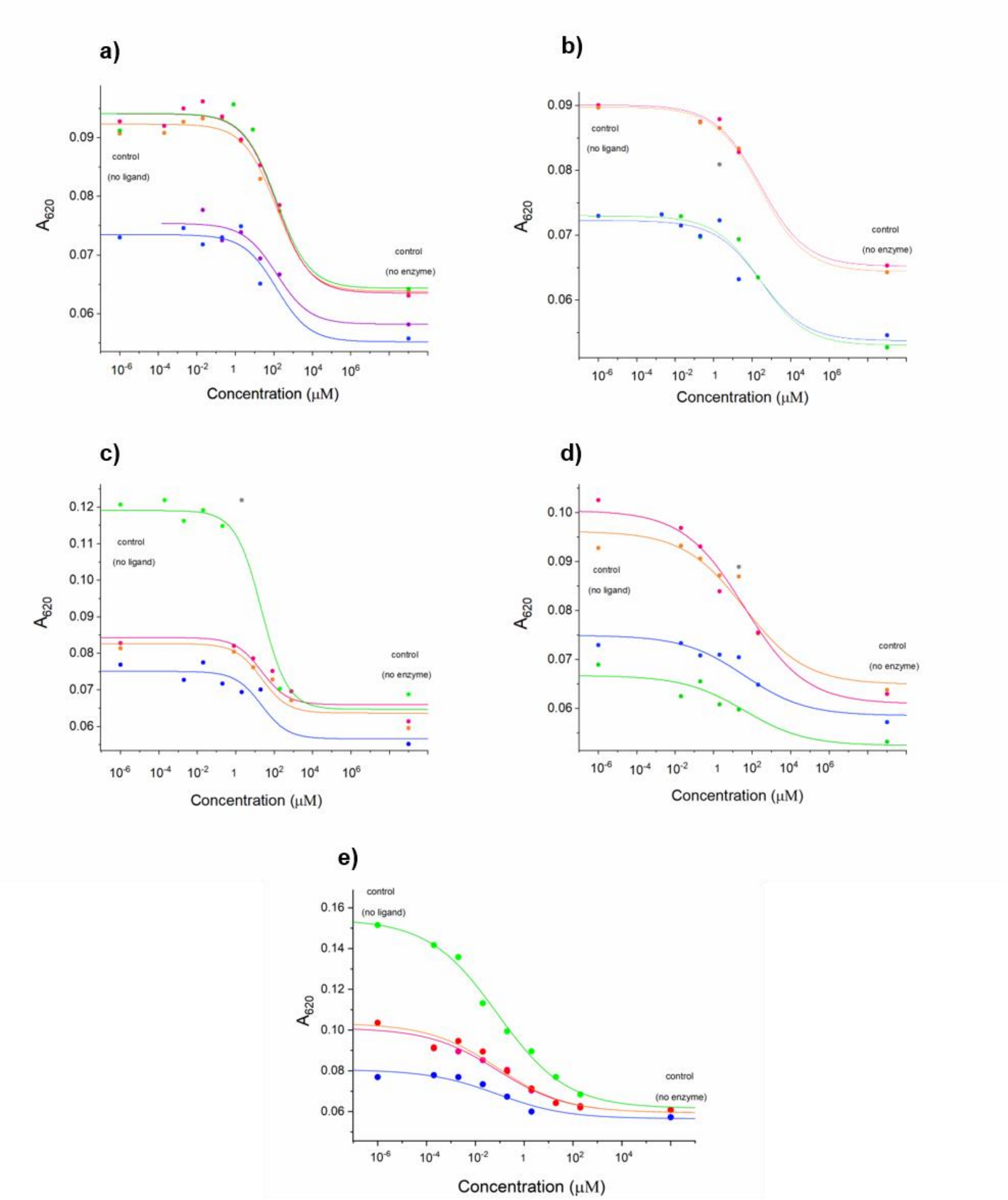

Figure S41. Absorbance at 620 nm used for IC_{50} value determinations of (a) **6a**, (b) **6b**, (c) **7a**, (d) **7b** and (e) tadalafil against PDE5. Both control data points are marked on graphs.
